# Supplementary material for: Integrating community health workers into the formal health system to improve performance: a qualitative study on the role of on-site supervision in the South African programme
Source: BMJ Open. 2019 Feb 27;9(2):e022186. doi: 10.1136/bmjopen-2018-022186 (PMC6398712; doi:10.1136/bmjopen-2018-022186)
Supplement: Supplementary file 1 [file bmjopen-2018-022186supp001.pdf]

Case site summary
Site: A Author(s): JG Date:22 02 2017

| | |
|---|---|
| **Model** | Clinic based team, although doesn't perform all standard clinic services (e.g. TB)<br>One PN, two ENs |
| **PN** – background/ experience, age, role, actual responsibilities | Professional nurse, midwifery, community health worker, at X for 6 years then Y hospital, retired at X, then went to the UK for X years .. started to work in Y at 62 .. now 72 years<br><br>**PNO:** once CHW have gone into the field, and have explained several things to the FW, spends an hour seeing patients.. and then leaves (so no meeting after CHW return)<br><br>**PNO_NM:** interview with FW, patient consultations for 2 hours; after 11.20 she reads patient files / notebooks; She also see family planning patients; (FW-PN doesn't seem to have a lot of work to do) |
| **EN**– background/ experience, age, role, actual responsibilities | <u>**EN01**</u> **- responsible for ward X**<br>**X** years old, became a EN in X, worked in private clinic before here, not from the area<br>FW comment: the EN seems passive.. maybe due to helplessness around CHW.. she struggles to maintain basic level of supervison by checking and doing CHW stats. Not clear how much time she spends in the clinic<br>*JG: from first meeting we know this EN doesn't like going out into the community .. it may be that she prefers to work in the clinic. When it comes to observations, she didn't actually do the stats because EN02 had given it to the data capturer to do because EN02 was going on training .. so sounds as if EN02 does most of the data reporting etc.*<br>**Observation day 1:**<br>Private meeting between PN and EN<br>Check on crèche that children have been immunized – no really confirmation of this<br>HH1: EN manages interaction with hh – follow up<br>Check on a pregnant woman at the crèche: registration done by CHW<br>HH2: patient there, but says that gate is locked;<br>HH3: patient not there<br>HH4: EN leads the interaction – follow up<br>FW: thinks EN went into the field because FW was there to observe .. but not real need for her to be there.<br>**Observation day2:**<br>Works in clinic as they are short staffed – so can't be observed; lunch and then knocks off as ½ day;<br>Next week she is on training so will ask receptionist to help with the stats. She will sign books to show she has checked them<br><br><u>**EN02**</u> used to work in critical care unit in private hospital<br>Not sure if I am a nurse any more .. but I feel more useful<br>Much more active, tries to solve problems, has set up whats app group, able to provide supervision<br>**Observation day 1:**<br>Creche campaign; EN is leading activities<br>**Observation day 2:**<br>Works in clinic as short-staffed; sorting patients and then doing child immunisations;<br>Comes out to check on CHW to allocate them to FW<br>HH1 CHW leads<br>HH2 CHW checks patient, but EN worried so asks questions about stomach<br>HH3: EN leads<br>HH4: EN asks about granny; in hospital; EN is shocked because here only 2 weeks ago, checks whether she has enough medication etc *(suggests that EN02 does go regularly to the field)*<br>HH5: jointly doing activities<br>HH6: CHW leads, didn't ask about child<br><u>HH7</u>: Jointly leading<br>Return to clinic at about 12 noon<br>*FW comment: EN is very supportive of CHW*<br>**Observation day 3:**<br><u>HH1</u>: on way to support group visited a lady who had given birth to twins- just checked on babies<br><u>Support group</u>: EN sits at a distance, CHW know how to do their work; she only intervened when a BP was too high, and when difficult TB case arrives (HH interview: CHW bring sputum bottle, but patient fails to provide) ; not 12 noon so there is time to see a few households |

| | |
|---|---|
| | <u>HH2</u>: not allocated to a CHW, but she checks on him because he is sick with TB; EN leads (as she hasn't been before); EN says they will come and do registration even though not in demarked area<br><u>HH3</u>: car accident victim and HIV<br><u>HH4</u>: EN quiet, CHW leads<br>Back on counsellors place by 1pm after 30 mins walk<br>**JG:** *EN seems works hard, happy to go and find extra households, seems to know some households – evidence that goes into the field regularly*<br>**Observation day 4**<br>Discussion about working on Fridays they do stats – cHW said we don't; EN said that is only the last Friday of the month .. so today they are going into the field; Later EN says later .. eish we are working on a Friday .. this is slavery<br><u>HH1</u>; came to give referral letter for NGO.. which is now closed for the year;<br>CHw says I will take you to the patient you didn't manage to see on Monday<br><u>HH2</u>: patient discharged from hospital in pain; EN tried to help – offered to call an ambulance, but woman said no because nobody to go with her; suggested clinic on Monday – ok, daughter will take me; asked for daugther's number; can't get it out of the phone<br><u>HH3</u>: checking on patient that they referred two days ago; she hadn't gone ; doesn't take her treatment;child without immunization; EN quite upset<br><u>HH3</u>: healthy baby; EN happy ☺ |
| **CHWs** – average details across team (Number, age, gender, years' experience, education) | X CHWs in FGD, (X reported to part of team) , all female, (another X waiting to be registered. PN wants them back)<br>Average age 39 (range25-49)<br>64% of have some secondary education;<br>29% have matric<br>7 years on average as a CHW (range 3-16)<br>Average number of households=262 (248-317) (only Sicelo and Biopatong gave specific numbers per CHW)<br>25 mins walk from home to clinic (range 2-50)<br>18 mins walk from clinic to households (2-45)<br>93% have phase 1; 21% have phase 2 |
| Community profile (eventually draw in survey data) | **PN:** Ward X contains a large informal settlement **(FDG SM?)**<br>Ward 10 predominately a township **(FDG AS?)**<br><br>**PN:** We started going from house to house, counting people during the registration.. according to where the CHWstayed.. initial plan was 250 households, but didn't work .. either 190 or 200 (if medical aid we skip it)<br><br>People report to us if somebody is sick, they will even break down the door<br>**ENO2** (ward X) – area is huge - shortage of CHW<br>3 areas within ward X<br>Ext X and X informal settlements / shacks – 5 is too big<br>Ext X – RDP house<br>People are moving to Rdp houses, go back 3 days later and people have moved..<br>Lots of Zim patients, but language not a barrier<br>Informal settlement, lots of crime, didn't feel safe at beginning,, but not easy for CHW to be robbed because most of them are well known in community |
| Challenges? (Not general) | **FM:** Storage is a problem – for files,<br>Safety .. too many break ins in the clinic<br>CHW need more training, and then more CHW will hopefully come later<br>**FDG-SM**<br><u>Equipment</u> – one CHW has take the BP machines that side .. so you call her to ask her to bring this side .. call from counsellor .. and we don't have machines<br><u>Behaviour of nurses</u> – some patients refuse to come to the clinic because of the bad treatment. However we encourage them for the sake of their health to ignore the nurses attitude P11<br><u>Queuing</u> – it irritates the patients .. so it gives us a problem when we try to encourage the patients to go to the clinic ..<br><u>Clocking in</u> WBOT promised we would work around where we stay.. they promised a clock in Ward 8 .. we have found a place for it .. it is tiring to have to come to ward 10 to clock in .. go back to ward 8 and then come back to the clinic .. it is a distrance and it is hot or raining!<br>Stipend, job security, career development, smart purse<br>**FDG_AS**: as above plus job safety, training, recognition and appreciation<br>We can't even go to the bank with this card, only to shoprite .. otherwise they charge us |

| | |
|---|---|
| | **EN02 Obs:** currently leave is pause .. because smart purse says 13 days, contract says 21 days – hey have sent a letter to smart purse – EN really interested in solving problems .. but sometimes above her |
| Planning (of the day) | **PN:** I have paired them, an intelligent one with a less intelligent one |
| Weekly pattern of activity (what do they do when?)<br>- overtime | **FM:** one of their strengths is tracing .. they will find the person p18<br>CHW say that they do overtime<br>**FM:** says they don't<br>**FM:** may be ENs help out in the clinic 1 day out of 5<br>Did two campaign days during FW<br>**EN01:**<br>Run support group for elderly patients on Wednesday<br><br>**FDG-SM:**<br>We follow up on defaulters<br>Household registrations each day, - it is not possible to do registrations at one time of the year<br>Home-based care – we don't do much of this ..<br>we refer to social services<br>*JG: not convinced there is a day for this, and a day for that .. rather that they do all of the activities when needed.*<br><br>**FDG-AS:**<br>With Kgatelopele the first week I deliver medication, the second I will check if they are taking it properly by counting.. if it doesn't balance .. then I will ask her why .. then I will report to the clinic to the cinic that I am following up on this person . |
| Daily pattern of activity | 6 hh per day (for two CHW) (average is 5)<br>13 mins average per household (average is 12)<br>10 mins average travelling between households (average is 14)<br>5 hours work per day (average is 4.40)<br><br>5% of activities are meeting with supervisors (not time)<br>20% of activities are HIV as condition; (TB 5.5%)<br>52.75% of activities are other chronic<br>5.49% as mother and child<br>53% of recipients are elderly<br><br>Between 8-9 am do training<br>**EN02**: training and gym until 9am.. e.g. with new machines I take two CHW and show them so that they know what to do in the field<br>You can see a big difference between those who have attended the trainings..<br><br>On a Tuesday they are taking medication to patients, and from there to check on what ever<br>So if we have gone to 2 households, taking treatment, then I pick one house, any house that we haven't been to before ..and do a registration ..<br>Cos you find that their focus is on the patients that they know, the patients that they know<br>But with me I am always squeezing in just one house that has never been registered .. so I don't totally change their routine .. just one HH and you will find problems<br><br>**EN01:** 8am clock in and wait outside until 9am when go to households (no mention of training)<br>CHW work in pairs must see 6 households 3, 3, lunch break is between 12-1<br>They don't just to go their patients, they also visit other households after a longer break when no new patient is identified. People in the area also inform the CHW when there is somebody who is sick;<br><br>**FGD-SM:** 6 households per day each and households registrations is too much .. and we have to be back here by 13.30 .. our lunch is at 12 noon. We discuss issues we have with the nurse and pack medication to deliver (next day or on way home)?)<br>We write everything in our note book .. and then we note in our diary when we have to return to a household<br><br>**FDG-AS:** planning seems to be more about which of your houses and which of mine<br>• Last time they told us we had to see 6 patients a day per CHW .. now they have changed it to 4<br>• If you are doing household registrations .. you can't do 6 per day p13 |

| | |
|---|---|
| | • Sometimes we do home-based care, if the patient is living on her own and she is on treatment . .if ask if she has eaten .. if she has't I cook for her;<br>• We refer them .. the provider needs 8rand<br>• Sometimes we teach the family how to bath them |
| Record keeping & Stats – how & when<br>- How notes/info are kept<br>- How they are consolidated<br>- Patient files<br>- Follow ups | **FM:** Has improved since two ENs arrived<br>WE don't have email and our transport is irregular;<br>Now doing it once a week .. we used to do it once a month .. but then it was late<br>Two ENs are taking responsibility and they forward to the data capturer and then we come back and chat about it (Sister Tenqui and FM?)<br>**PN:** they have notebooks that they write in .. not more than 5/6 households per notebook .. (*so does that mean that they have 40 notebooks?)* I read those books every time I get a chance<br>**EN02:** she does monthly stats, she checks individual stats, talks to CHW saying why do you say this, or why haven't you write x, gives training to CHW.<br>In training we were told it would be easier if we did it weekly so I said to CHW lets do it on Fridays .. lets not go ot households but do stats instead .. now we are doing it monthly because we are faster now<br>From CHW to EN to facility manager<br>*JG: this is why they don't work on Fridays.*<br>**FDG-SM** we do stats daily .. at lunchtime we record evertying (some confusion about recording number of children)<br>**FDG-AS:** the OTL takes time to check them<br>I do them in my lunch hour, or at home, or when we are seeing my partner's patients |
| Referrals<br>- Forms/documentation<br>- Queueing<br>- Files | **FM** :Patient meant to show form at door and then goes to see Sister X, who then completes the form and gives back to the carer (if another nurses see patient they also complete the form)<br>**EN02:** Report number of referrals in stats as well<br>**FDG-SM**<br>"The referral form is causing trouble with the patients. The patients are supposed not to follow the queue, they should go straight to the PN but the patients are been shouted, told to go and follow the queue. Now the patients are fighting with us because we told them that with the referral they don't have to queue."p 15 |
| Medication delivery | **FM:** ENs can't come issue medication; so tengiwe does it . and if she is not here, they come to me with the vital signs data and the pill count, the condition of the patient, and the next clinic data. If the ENs are there the CHW will do this<br>**FDG-AS:** when you count their medication, sometimes they start shouting at you . you try to tell them that I am tyring to help you .. |
| Relationships Clinic – WBOT team | Other sisters will give medication out if necessary<br>CHW refer patients to specific sisters eg. For TB<br>**EN02** We do in-service training .. Sister X is doing EPS .. she is very helpful .. she is not WBOTS but she is very helpful - she is a staff nurse, but she has got more xperience … she attended severe malnutrition training .. she called all the CHW and patients .. shw as doing a presentation for everybody .. so it is not only us doing that .. She is also helping when we need something There is support from the clinic.<br>We engage with the FM for everything, problems we have, reporting .. she is part of the community, we include her in everything<br>District coordinator – I have only seen her once, when she came with those two ladies from wits !<br>Training person at X– if I have aproblem a message the OTL group, or even phone her .. she is giving support.<br>**FDG-SM**<br>"At the clinic we are treated as if we are not part of the clinic, they talk to us as if we are children. They shout at us in front of the patient that you brought for help. We don't have support at all. Another thing, they don't want us to eat breakfast but they eat when they come to the clinic. We consult TB patients on empty stomach. I did raise that to the PN and she said we should eat at home. Then I asked why she is not also eating at home in the morning and see if she can do it. In the morning we prepare our children to go to school, others have husbands to take care of before we leave at 7:00am, and we have to walk. We don't have time to have breakfast at our houses in the morning. We only have breakfast and lunch together at 12:00pm. There is no support at all." P15<br>**FDG-AS:**<br>"the sister in charge and our OTLs are treating us like.. I don't know .. as you can see we have no place to stay, no chairs, no table, nothing .. we came with the idea of starting the day with a prayer like in hospitasl.. but the sister in charge says we won't do such a thing here .. so we stopped because we are powerless' p14<br><br>**Any conflict with OTL or clinic staff** |

| | |
|---|---|
| | "We are all going to talk neh .. add to what I say .. we have our sister in charge, but we have another one .. the CHWs know who I am talking abut .. this sisteris controlling us more than our OTL" .. P21<br>"she even shouts at us infront of the patients. We are adults, mothers and we have our own houses and when we are here she treats us like children. She does not respect us." P21<br>"And sometimes we walk in, she shouts and says 'hey you is that a uniform, that is not the way we dress here.' Where are we going to get the new navy pant? That pant costs R190-R200.00. All that shouting she does it in front of everyone at the clinic. That is not okay and when you talk back to her, she will say that she is not our OTL and does report to us."<br>**Participant**: "We want her to be out of the WBOT team. We want to report to our OTL only. She is responsible for other nurses not us."<br><br>"My challenge as a CHW is that when I do home visit, some households do not have food to eat and the patient is on treatment. So I have to empathise with the patient, contribute something so that the patient can eat. When I come to the clinic and ask for this porridge, the clinic staffs tells me that I have to come with the patient so that they can see the patient. That is a big problem because the patient is sick and has no transport money to go to the clinic. So yah it hurts." P20 |
| Relationship EN & PN | **EN02:** if I need clarity I will phone here |
| Supervision of CHWs (including training) | **PN:** I read their books whenever I can; if they have difficult cases they bring them straight to me .. and I take the car and drive to see the issue ..<br>**EN01:** Most of the CHW are older than her.. they don't always listen to the EN. When they only have one household to visit they have all sorts of stories to get away. She reported this to the FM who said she would sort it out.<br>Two CHW communicate on behalf of the rest ..<br>They report to the EN when they have found a defaulter, they then revisit and then report<br>Weekly or bi-weekly she checks the activities of the CHW<br>FM told CHW not to come to work during strike<br><br>**EN02:** supervises 8 CHW, 5 have not been registered so they are using the book. If she knows CHW have much work to do, she leaves with them at 8 rather than 9<br>Different types of CHW – counsellor, peer educators, rest are CHW; one CHW is the leader whose job it is to forward complaints to her<br>If I have been on training I will ask how did it go with that patient, we have an whatspp group so we can communicate that weay .. if I am not around, they will send me a whatsapp and I will say do this or that.<br>I spend the whole day with them, .. and then I give them a lift home<br><br>Since the strike they have not been the same .. some they understand, some they are aggressive … so I said if you need something, or clarity, sit as CHW, write a memo /list and let your head CHW delivery it .. according to the hierarchy – this really helps, otherwise we have too many complaining and nothing will be sorted out<br>e.g. bags – why only 10 bags; so they wrote it down, I took it to my PN, and the facility manager who was able to sort it out; we discussed how they are going to use the equipment<br>*JG – example of sorting things out*<br><br>It goes back to the in-service training … when I am compiling the stats I am able to see this one is struggling .. when I do supervision I can see this one didn't do it right … I keep quiet in the house,, but immediately we step outside .. as we are walking I do spot training<br><br>Both FM and EN01 had stories of truancy (going home and coming back to clock out);<br>EN02 uses her car to drive them back to the facility if they need to clock out, or allows them to sign out tomorrow if they are working late<br>**EN 02** Sometimes they come back early because of the distance.. because it can be an hours walk … you can waste 2 hours a day walking backwards and forwards<br>**FDG-SM** do you work overtime? If we do household registration, and we don't find anybody who can has the information, we go back in the afternoon or the weekend .. .. Sometimes we deliver medication in the afternoon.. sometimes we are do vital signs at 7.45 |

| | |
|---|---|
| | **FDG-AS:** if some people are very sick we monitor them over the weekend<br>Our team leader is supportive .. if the patient wants to know something .. I ask her |
| Supervision of ENs | **FM:** do induction and appraisals of EN; provide training – HIV (going on Monday), TB (one has gone the other will go in 2017), EVP<br>**EN01:** if sister X is not around she goes to the FM and she provides a solution<br>They help out between 7.30 and 12.00 at the clinic when the clinic is full. When the clinic needs them they pick two CHW to stay and help the EN .. but they are free to say no<br>**EN02:** The facility manager makes sure we attend regular training to uplift ourselves She organizes workshops with CHW to train them on what she has learnt<br>Now I can lead campaigns – cos I understand everything about the campaign – so you see there really is development here..<br>My job is with WBOTs. 70% in the field, 30% in WBOT admin .. if I am not busy I help with vital signs stations.. it is not part of my job but I do it anyway .. I refer my patients and the nurses see them .. So there is teamwork .. The support systemis great so I never felt pressure to leave my wBOT duties<br>**FDG-SM**<br>"And sometimes when you go with the OTL, the patients are feeling uncomfortable because she shouts at them. And we are unable to tell her that what you are doing is wrong, you are not supposed to talk like that with patients. Now the patients are telling us that we are not welcomed in their houses if we come with the OTL."<br>*JG- not clear which OTL is this. .. although know it is ward X* |
| Level of knowledge of CHWs | **FM:** Depends on education of the individual - some will do as you ask, others you need to dig deeper ..<br>**PN**: they are done some of the training (phase 1 and 2)<br>Are they able to implement it ? some are .. others are dragging, but they are trying p7<br>**FDG-SM – what cases aren't you able to deal with?**<br>Sick patient with no food >refer<br>Elderly patients who needs to be in old age home |
| Travel (time, mode, particular features/challenges) | |
| Resources Available | |
| Resources Needed | Have to buy batteries, transport, airtime (although do call-backs), forms, uniform out of own money (although towards the end, the CHW were wearing pink shirts)<br>Don't have office space, or masks for working with TB patients<br>**PN:** forms .. yes, one day a teacher was here from the primary school.. and I asked him to make copies<br>*No photocopier*<br>They need a permanent structure so that they can do maternity<br>We are using our own transport - that is what is irritating<br>**EN02:** Shortage of bags<br>**EN02:** I ma suing my own petrol, my own cellphone, chw use their own airtime.. sometimes they even call patients.<br>We need a place to meet as CHW /OTLS; patient files are stored in PN's room<br>We need our own social worker<br>**FDG_SM – description of the bag**<br>We are meant to screen everybody over 25 years for diabetes.. but we don't because the strips are not enough<br>The machine is already broken .. it is not strong<br>We don't have an office, airtime, batteries (we have to buy out of our money)<br>**FDG-SA:** we were given 10 bags for 17 of us; my machine isn't working.. I reported it .. it is embarrassing when it doesn't work; the strips are finished; we report to the sister who says that the patient must come to the clinic<br>You use your own airtime to call an ambulance;<br>If you are tracing, if you don't find that person at the address, you call them using your own airtime .. otherwise how are going to find them them<br>What makes us not to achieve more is that when we come to the clinic and ask for some more equipment, we are told that that they don't have' |
| Community relationships | **FM:** Initially not accepted, were chased away .. now they are accepted. ..have built rapport<br>community unhappy when CHW on strike (they were saying nurses were on strike p6 |

| | |
|---|---|
| | can do dressings which patients appreciate<br>**PN**<br>• The relationship between the community and the CHW .. 'one day we were out walking with Dr Y and Dr X.. and somebody asked the CHW how come you are walking with so many men' .. she answered they are doctors we are working together ' Dr X asked theperson .. how do you know my nurse? .. she brings me medication every month P6 .. Dr X was very excited that they were well known in the community p6<br>• That is why we allocate houses to them in their area.. because then people can call them over the weekend . and then they give us a report and say .. a patient was giving birth .. and so we called an ambulance etc .. it is better if the CHW is in the area<br>• We don't have a clinic committee .. but we have a very good relationship with the ward councilor (ward X?) (EN02 as well – if there is somebody who is sick he calls me, so we work toghether)<br>• I want to start a support group for young people with HIV<br>**EN02**<br>The community is aware of us .. they are coming to us. Even the referral forms its good<br>People are taking treatment now. I think there is lesser tracing on ARV patients, most defaulters you will find are TB. Our stats are going up<br>**Reason for success**<br>Employing us as staff nurses .. cos if the CHW see you as supporting them, trying to understand where they are coming from, especially with this employer thing is mixed up … That is why they refused to go on strike today<br>**EN02**<br>When they were on strike for 3 months .. the patients were complaining .. where have you been, we have been waiting for you .. they decided not to go to the clinic but wait until the sisters were back .. so they really rely on them<br>**FGD-SM**<br>We get the list of defaulters .. but now there are few because of our work .. people have now accepted their illness .. people do appreciate us.. just come and disclose to us .. and later that person will come and thank you for the work you have done p5<br><br>Iyhoo, I'm so proud about my patients, they get better. Others I found them bedridden but because of us they have recovered. Even if they don't give me any material thing, but when I pass them on the street and she/he tells others that she/he is walking because of me, I feel satisfied because at least I have made other people better." P15<br><br>"In ward 8 we don't have any conflict with the community because the ward councilor had introduced us during the public meeting. They know us and they trust us in a way that when they need help even if it is during the night, they wake us. People got that belief that when the CHWs call the ambulance, the ambulance arrives quickly. So we are don't mind because we know the community rely on us."<br><br>We already said, they are able to come to us. Like no the nurse at the clinic is unapproachable, she talks to the patients the way she likes and the patient keep quiet but when they see us, they tell us what happened with the nurse at the clinic. So it is easy for them to talk to us than to the nurses. Other patients said if the nurse comes to their houses and speaks the way she speaks when she is at the clinic, we are going to hit her. Then we try to calm them down." P15<br><br>If my neighbour is sick I ask my partner to attend to her.. cos I don't want the neighbour to say that I talk about her ..<br><br>"The community things we are their helper, they love us. In everything that we do, they stand with us." P25<br><br>"To honest the patients trust us and they appreciate us. I have a patient who is HIV and she doesn't want to come to the clinic and I don't want my patient to default. So I promised her that I will assist. Then I came to the clinic and talk to the sister. I said sister my patient is working at the farms so please pack medication for her, here is her clinic card. I'll ask her to come next month. The sister scolded me because the patient did not come to the clinic on her last appointment. So I begged the sister and I promised the sister that I will bring the patient myself. Then it was sorted, the patient came to the clinic with me and she got her medication." P18 |
| Relationships with other NGOs, | **EN02**<br>We work with policing forum, ward counsellor, we have their numbers .. we do campaigns together .. community and health campaigns (e.g.creches) |

| | |
|---|---|
| networking, knowledge of context<br>(draw on what exists & links clients) | Household registration for municipality .. although you ask these questions about income.. they think you are bringing food .. so they make you feel like you are making empty promises)<br>NGO A – I have referred too many people there .. but there results are not good.. if people don't have an ID they come back with nothing. We need our own social worker |
| Overall impression<br>- Incl do CHWs feel valued?<br>- Other notes | During the strike some of the CHW tried to work .. but the EN and the FM told them not to<br>**EN02:** strike started again today but CHW refuse to be part of strike (strike is for better employment issues, high bank card deductions (20R), lack of communication from smart purse, uniform); eventually they had to accepted it .. so not joining strike today<br><br>**Can the programme be led by staff nurses ?**<br>**EN02** I am saying this because with staff nurses in the community … I can deal with almost 90% of the problems .. but as a staff nurse I am not allowed to give out treatment.. if I pre-pack medication .. it has to be checked by the professional nurse .. also I can't consult |
| Interesting points | • FM is involved, and knows how the WBOT programme runs, clinic was set up and the same time as the WBOT<br>• EN02 know there is a reciprocal relationship between WBOT and clinic and need to maintain that ..(helps when clinic is busy and they see her patients)<br>• If clinic is not welcoming to patients .. difficulty in persuading patients to go to the clinic .. So success of WBOT is dependent on quality of care in clinics<br>• In EN only model – a PN has to be available to dispense the medication (or check it?)<br>• It seems that EN02 who goes out into the community has a better relationship with the CHW, the trust each other more .. a lot of the problems are about lack of trust<br>• EN 01 is a lot more passive and prefers to work in the clinic;<br>• PN sees patients but doesn't seem to meet with CHW when they return .. EN02 knows households and really works with CHW |
