# Supplementary material for: Integrating community health workers into the formal health system to improve performance: a qualitative study on the role of on-site supervision in the South African programme
Source: BMJ Open. 2019 Feb 27;9(2):e022186. doi: 10.1136/bmjopen-2018-022186 (PMC6398712; doi:10.1136/bmjopen-2018-022186)
Supplement: Author's manuscript [file bmjopen-2018-022186.draft_revisions.pdf]

BMJ Open is committed to open peer review. As part of this commitment we make the peer review history of every article we publish publicly available.

When an article is published we post the peer reviewers' comments and the authors' responses online. We also post the versions of the paper that were used during peer review. These are the versions that the peer review comments apply to.

The versions of the paper that follow are the versions that were submitted during the peer review process. They are not the versions of record or the final published versions. They should not be cited or distributed as the published version of this manuscript.

BMJ Open is an open access journal and the full, final, typeset and author-corrected version of record of the manuscript is available on our site with no access controls, subscription charges or pay-per-view fees (http://bmjopen.bmj.com).

If you have any questions on BMJ Open's open peer review process please email info.bmjopen@bmj.com

BMJ Open

# The effect of full time supervisors on the performance of a South African community health worker programme: An observation study

| | |
|---|---|
| Journal: | *BMJ Open* |
| Manuscript ID | bmjopen-2018-022186 |
| Article Type: | Research |
| Date Submitted by the Author: | 07-Feb-2018 |
| Complete List of Authors: | Tseng, Yu-hwei; University of the Witwatersrand School of Public Health, Centre for Health Policy<br>Griffiths, Frances; University of Warwick Warwick Medical School<br>de Kadt, Julia; Gauteng City-Region Observatory, a partnership of the University of Johannesburg, the University of the Witwatersrand, Johannesburg, Gauteng Provincial Government and organised local government<br>Nxumalo, Nonhlanhla; University of the Witwatersrand School of Public Health, Centre for Health Policy<br>Rwafa, Teurai; University of the Witwatersrand School of Public Health, Centre for Health Policy<br>Malatji, Hlologelo; University of the Witwatersrand School of Public Health, Centre for Health Policy<br>Goudge, Jane; University of the Witwatersrand School of Public Health, Centre for Health Policy |
| Keywords: | Human resource management < HEALTH SERVICES ADMINISTRATION & MANAGEMENT, Organisation of health services < HEALTH SERVICES ADMINISTRATION & MANAGEMENT, QUALITATIVE RESEARCH, PUBLIC HEALTH, Health policy < HEALTH SERVICES ADMINISTRATION & MANAGEMENT |
| | |

SCHOLARONE™
Manuscripts

# The effect of full time supervisors on the performance of a South African community health worker programme: An observation study

Yu-hwei Tseng,[1] Frances E Griffiths,[1,2] Julia De Kadt,[3] Nonhlanhla Nxumalo,[1] Teurai Rwafa,[1] Hlologelo Malatji,[1] Jane Goudge[1],

**Affiliations**
[1] Centre for Health Policy, School of Public Health, University of the Witwatersrand, Johannesburg, South Africa;
[2]Division of Health Sciences, Warwick Medical School, University of Warwick, Coventry, United Kingdom
[3]Gauteng City-Region Observatory, a partnership of the University of Johannesburg, the University of the Witwatersrand, Johannesburg, Gauteng Provincial Government and organised local government, Johannesburg, South Africa

**Address correspondence to:**
Jane Goudge
Centre for Health Policy (CHP)
School of Public Health, Faculty of Health Sciences, University of the Witwatersrand
Private Bag X3 Wits 2050, Johannesburg, South Africa
Tel: +27 11 717 3425; Email: jane.goudge@gmail.com

**Keywords:** community health worker, supervision, South Africa

**Abstract word count:** 252

**Text word count:** 4,896

**Tables:** 2

**Number of references:** 29



For peer review only - http://bmjopen.bmj.com/site/about/guidelines.xhtml

## ABSTRACT

### INTRODUCTION

Community health worker (CHW) programmes have the potential to improve access to primary health care and so assist in achieving universal health coverage. However, supervision in CHW programmes is often weak. South Africa is implementing a national CHW programme with full-time supervisors. In this paper, we report on an observation study in which we compared the supervision of six CHW teams in Sedibeng District to describe different supervisory practices and to explain how they affected CHWs' performance.

### METHODS

In the first part of a 3-year mixed method intervention study, we employed qualitative methods to study CHW teams with three different configurations of supervision: 1) clinic-based team supervised by a senior nurse and a junior nurse; 2) health post-based team supervised by a senior nurse and a junior nurse; and 3) clinic-based team supervised by a junior nurse only. We conducted CHW and supervisor observations (126 days), focus group discussions (12), key informant interviews (43), and client household interviews (74).

### RESULTS

Effective supervisors supported CHWs through household visits, on-the-job training, debriefing, review of CHWs' daily logs, assistance with compiling reports and administrative matters. CHW teams led by senior nurses were better motivated and performed a greater range of tasks. Those based in a clinic were more able to ensure a continuity of care for their patients. Junior nurses were more able to fulfil their role when supported by senior nurses.

### CONCLUSION

Full-time senior nurse supervisors provided on-going training, facilitated cooperation of facility staff, liaised with the community, and so improved CHW performance.




For peer review only - http://bmjopen.bmj.com/site/about/guidelines.xhtml

**Strengths and limitations of this study**

- To the best of our knowledge, this is the first study to describe dedicated, full time supervisory practices in a CHW programme.
- Policy implications concerning optimizing the effectiveness of CHW programmes are evident, e.g. supportive supervision, and development of new generation supervisors.
- The research employed multiple qualitative methods to collect a substantial amount of data, capturing different stakeholder perspectives and their behaviours.
- Major limitations to the study include the observer effect during data collection and representativeness of the sample.

For peer review only



For peer review only - http://bmjopen.bmj.com/site/about/guidelines.xhtml

## INTRODUCTION

Community health workers (CHWs) play an important role in improving health care coverage and access in low and middle income countries.[1-3] However, optimizing the contribution of CHWs continues to be a global challenge. CHWs, with no professional qualification, perform primary health care related functions in the community.[1, 4, 5] They work on the periphery of the health system and often in under-served communities where needs are immense and multifaceted. As a result CHWs require frequent and supportive supervision.[3, 6]

Supervision of health care workers requires: *"defining objectives, training, monitoring performance, solving problems, motivating and supporting health care workers to improve their performance."*[7] Good supervision improves coverage, quality of care, motivation and retention.[8-13] It is integral to sustaining and scaling of CHW programmes,[14, 15] and to building strong relationships with the community.

However, supervision is often one of the weakest elements in CHW programmes.[3, 16, 17] Existing literature includes limited descriptions of supervision - its availability, frequency and location. [18] In many instances supervision is no more than periodic interactions by facility staff or officials, who may not understand the context and role of CHWs. This often results in bureaucratic inspection rather than support.[1, 6, 18-21] South Africa is in the process of developing a national CHW programme, with full-time supervisors, whose responsibility is to manage, train, mentor, monitor CHWs, promote teamwork and to facilitate links with the health system and the community. This study examines CHW teams with 3 existing


For peer review only - http://bmjopen.bmj.com/site/about/guidelines.xhtml
For peer review only

configurations of supervisors and locations – 1) clinic-based CHW team supervised by a senior nurse (SN) and a junior nurse (JN); 2) health post-based team supervised by an SN and a JN; 3) clinic-based team supervised by a JN only – in Sedibeng District of Gauteng Province to illustrate how they affect CHWs' performance.

## METHODS

We report on an observation study that examined the operation of six CHW teams by adopting qualitative methods, as the first part of a 3-year intervention study.

### Study site

In South Africa, until recently, CHWs were employed by non-governmental organizations (NGOs) often providing services focused on a single condition. In some provinces, CHWs have now been absorbed into a government programme in an attempt to shift from the patchwork of services to a programme with a national scope. CHW training covers ante-natal and post-natal care, monitoring adherence among patients with chronic diseases, immunisation for under 5s, screening for malnutrition, TB, gender based violence, and making referrals to health, social and other services.

While Sedibeng district is relatively affluent by South African standards, over 20% of its residents fall below the food poverty line. Outside well-developed urban areas, disadvantaged communities, with inadequate shelter, food insecurity and high disease burdens, have limited access to services such as clinics, transport, water and electricity.



For peer review only - http://bmjopen.bmj.com/site/about/guidelines.xhtml

Two years before the introduction of the national CHW policy, Sedibeng adopted Cuban-style health posts in a few areas in order to build a closer link between a clinic and the community. Each health post was staffed by a CHW team, and a professional nurse who could supervise CHWs, and consult patients with minor illness and prescribe medication. When the national programme took effect in 2011, it also incorporated these health posts. [22] At the time of the study there were 39 CHW teams in 37 of the district's 72 wards (geopolitical subdivisions of municipalities in South Africa). Sixteen teams were based in health posts and the remaining 23 were clinic-based, with the same responsibilities. The teams, with between 6 and 20 CHWs, provide services covered by the national training. In addition, they register households to identify health needs, deliver medication to elderly or disabled chronically ill patients, trace non-adherent patients, follow up on those newly discharged from hospital, and provide some home-based care.

In Sedibeng, as in other parts of South Africa, CHWs operate in challenging conditions. The CHWs are not formally employed by the government, and as an outsourced workforce, their remuneration, benefits, job security and career path are poor. Often CHWs based in facilities are seen as outsiders by facility staff. An electronic system requiring CHWs to clock in/out at the clinic prevents them from reaching outlying areas as they travel between the clinic and these areas on foot. CHWs struggle to contact the private payroll company responsible for monitoring attendance and paying stipends, when they experience problems. Two months prior to the start of our fieldwork CHWs went on strike over these issues. CHWs commonly work without necessary resources, including equipment, stationery, and funds for transport or communication. A team of 20 CHWs may share 1 or 2 blood



For peer review only

For peer review only - http://bmjopen.bmj.com/site/about/guidelines.xhtml

pressure machines borrowed from the facility. Even when equipment, such as a glucometer, is provided, the associated consumables such as glucose strips are often not replenished. Notebooks CHWs use to record daily activities are purchased out of their own meagre stipend. As clinic buildings are often small, a lack of space to hold meetings, complete paper work, or store files also hampers their work.

**Selection of CHW teams**

The research team consulted with district officials, categorizing the CHW teams into model 1 – clinic-based team supervised by a senior nurse (SN) and a junior nurse (JN); model 2 – health post-based team supervised by an SN and a JN; model 3 – clinic-based team supervised by a JN only. Two teams of each type of model were included in the study. Table 1 describes the characteristics of different teams and their catchment areas.

**Table 1. Supervision configurations of CHW teams and their catchment areas**

| Model | Supervisor | Based in | Site Code | Description of catchment area | Max. distance CHW base to border |
|---|---|---|---|---|---|
| 1 | Senior Nurse[1] & Junior Nurse[2] | Clinic | 1A | Township[3] with relatively well-off households and apartheid-era housing | 2 km |
| | | | 1B | Township with RDP[3] housing and informal settlement[4] | 3 km |
| 2 | Senior Nurse & Junior Nurse | Health post | 2A | Densely populated semi-urban area | 1 km |
| | | | 2B | Large informal settlement, with many migrants | 1 km |
| 3 | Junior Nurse only | Clinic | 3A | Rural, informal settlement, also with RDP housing and farm plots | 6 km |
| | | | 3B | Rural, township and farm plots | 25 km |

[1] A senior nurse supervisor has a professional nurse qualification; [2]A junior nurse supervisor has an enrolled nurse qualification; [3] Townships in South Africa usually refer to urban areas built on the peripheries of towns and cities for non-whites; [4]RDP: Reconstruction & Development Programme, a policy under which the construction of low-cost housing units started, post 1994; [4] informal settlements: areas where impoverished population build shacks on vacant land.

As Table 1 shows, sites included formal townships, informal settlements and rural areas. The distance from the periphery of the area served to the clinic or health post

For peer review only


For peer review only - http://bmjopen.bmj.com/site/about/guidelines.xhtml

ranged from 1-25km. Each health post was a 2-room temporary structure without electricity, and one of them had irregular water supply.

**Data collection**

Trained field workers observed pairs of CHWs and their supervisors, and wrote detailed field notes guided by a template. Among a total of 126 person-days, each subject was observed for 3-5 days to capture their daily routine. Between September 2016 and January 2017, field workers conducted 12 focus group discussions with CHWs and 43 in-depth interviews with key informants including supervisors, facility managers and community representatives. These interviews gathered data on CHW responsibilities, challenges, achievement, support, and community engagement. Seventy-four household members, who received referral advice from CHWs during observed home visits, were interviewed a month later, to understand their experience of the CHW service, whether the referral advice was acted upon, and whether the follow up service was effective. Data collection took place between September 2016 and January 2017.

**Data analysis**

We used thematic content analysis involving both deductive and inductive approaches to code data and identify themes. Each co-author studied all the data from one or two sites and generated a site-specific summary, based on a template, drawing together all data for that site. An iterative process continued throughout data analysis and manuscript writing, as revisiting the summarised and raw data was necessary to confirm descriptions of the context, enrich the content, provide clarity, and check emergent ideas. All data were analysed manually.



For peer review only - http://bmjopen.bmj.com/site/about/guidelines.xhtml

In terms of quality assurance, the authors generated and compared duplicate summaries to ensure inter-coder reliability. Multiple data sources, as well as events documented at times by multiple field workers, allowed triangulation, which increased our confidence in the validity of the findings.

**Ethics**

The study protocol and instruments were approved by the University of the Witwatersrand's Human Research Ethics Committee (Medical) (M160354), the Gauteng Provincial Health Research Committee in South Africa, and the Biomedical & Scientific Research Ethics Committee (BSREC) at Warwick University (REGO-2016- 1825), United Kingdom. All participants gave informed consent.

For peer review only



For peer review only - http://bmjopen.bmj.com/site/about/guidelines.xhtml

## RESULTS

### Who provides supervision?

Senior nurse supervisors (SN) were those with professional nurse qualifications able to diagnose patients, prescribe treatment and dispense medication. More importantly, they were also trained in primary health care and community nursing. As rehired retirees, they each had more than 30 years of experience as a nurse before they joined the programme (Table 2). Junior nurse supervisors (JN) were those with enrolled nurse qualifications who could provide nursing care under supervision after completing a 2-year nursing course. The length of the JNs' work experience ranged from 6 months to 14 years and the longest any JN had been in their current role was seven months. Most of the JNs were younger and less experienced in community work than the CHWs they supervised.

### What happens during supervision?

Supervised home visits

Across sites, JNs routinely accompanied different pairs of CHWs on home visits several days a week. On these trips, some JNs demonstrated sensitivity in correcting CHW practice. *"When I can see this one didn't do it right, I keep quiet in the house, but immediately after we step outside as we are walking I do on-the-spot training" (JN, 1B)*. Other JNs, particularly those working without an SN supervisor, seemed uninvolved during the home visits, and did not initiate any discussion about the household afterwards. One JN spent 4 days out in the community, but her CHWs did not acknowledge her as contributing as a supervisor, *"When we get there she's doing the same job that I normally do when I am alone." (CHW-FGD, 3A)* Consequently, her CHWs were not keen to report problems they encountered in the field, such as



For peer review only - http://bmjopen.bmj.com/site/about/guidelines.xhtml

suspected TB patients or being denied entry to households, as they had little trust in her problem solving capability.

**Table 2. Supervision of CHWs – who, what and when**

| MODEL | SN / JN clinic-based | | SN / JN health post-based | | JN clinic-based | |
|---|---|---|---|---|---|---|
| SITE | 1A | 1B | 2A | 2B | 3A | 3B |
| Who supervises and is supervised | | | | | | |
| **SN supervisor** | | | | | | |
| Age (years) | 65 | 72 | 66 | 59 | n.a. | n.a. |
| Years as nurse | 36 | >30 | 38 | 39 | n.a. | n.a. |
| Years in programme | 4 | 4 | 4 | 4 | n.a. | n.a. |
| **JN supervisor** | | | | | | |
| Number of JN in team | 2 | 2 | 1 | 1 | 1 | 1 |
| Mean age (years) | 43 | 29 | 28 | 25 | 36 | 31 |
| Mean years as nurse | 14 | 2.5 | 0.5 | 3 | 5 | 2 |
| Years in programme | 0.6 | 0.6 | 0.6 | 0.6 | 0.3 | 0.3 |
| **CHW** (supervisee) | | | | | | |
| Number of CHW in team | 16 | 17 | 9 | 12 | 14 | 20 |
| Mean age in years (range) | 38 (26-53) | 39 (25-47) | 34 (27-45) | 37 (26-51) | 42 (23-58) | 33 (23-54) |
| Mean years (range) as CHW | 7 (4-12) | 7 (3-16) | 7 (6-9) | 6 (3-12) | 10 (3-9) | 6 (5-17) |
| **Formal training of CHWs** | | | | | | |
| % CHW received phase 1 training | 91% | 93% | 100% | 90% | 5.6% | 16.7% |
| % CHW received phase 2 training | 91% | 21% | 100% | 46% | 0% | 0% |
| Supervision activities | | | | | | |
| **Supervised home visits (day/week)** | | | | | | |
| By senior nurse | n.o. | n.o. | 1 | Occasional | n.a. | n.a. |
| By junior nurse | 3 | 4-5 | 4-5 | s.s. | 4 | On request |
| **On-the-job training** | Yes | Yes | Yes | Yes | n.o. | s.s. |
| **Regular debriefing** | Daily | n.o. | Daily | Weekly | s.s. | s.s. |
| **Examining daily logs and registers** | Yes | Yes | n.o. | Yes | n.o. | n.o. |
| **Assisting in reporting** | Yes | Yes | n.o. | Yes | n.o. | n.o. |
| Frequency of preparing reports | Weekly | Monthly | Monthly | Weekly | Weekly | Monthly |
| **Resolving administrative matters** | Yes | Yes | Yes | n.o. | n.o. | n.o. |

Notes: n.a.: not applicable; n.o.: not observed & not indicated in other information sources; s.s.: the activity was mentioned by a single source other than CHWs, but was not indicated in other data sources.

Formal and informal (on-the-job) training

In four of the six sites, 90~100% of the CHWs had completed the first phase of the national training programme, but completion of Phase 2 was only high in two sites



For peer review only - http://bmjopen.bmj.com/site/about/guidelines.xhtml

(Table 2). Phase 1 is a written test asking participants to describe the purpose and structure of the CHW programme. Phase 2 involves a practical assessment of the skills of the CHW in actual household visits; success in this assessment requires comprehensive and on-going training. One SN placed considerable emphasis on ensuring that her CHWs had received all the appropriate training. *"When SN came, she checked who had done what course and organized training for those who needed it."* (*CHW-FGD, 1A*). This SN also used daily morning meetings as training sessions, *"our SN is teaching us … every day in the morning." (CHW-FGD, 1A).* Given the volume of information that CHW were required to retain, these regular sessions were important. This SN involved the two JNs in the training: *"Sister teaches us like nobody's business" (JN, 1A)*, enabling the JNs to take up the supervisory role.

At the JN-only sites, supervisors were unable to arrange formal training for the CHWs, and did not provide informal training themselves. The CHWs also did not receive informal training from other facility staff. *"It's been a while since we had training. We used to have training while we were working under the NGO. Trainings are very important because they upgrade the information we have." (CHW-FGD, 3B)*

Regular debriefing

One health post-based SN/JN team (2A) held daily debriefing sessions, similar to case reviews, at which CHWs described the households visited, problems encountered and actions taken. The supervisors, and sometimes CHWs, would give comments, suggestions or further instructions. The sessions strengthened CHWs' knowledge base and problem-solving abilities, and provided a platform for collective supervision, enabling the CHWs to learn from each other's experience. On the other hand, as



For peer review only - http://bmjopen.bmj.com/site/about/guidelines.xhtml

CHWs had to do the task organisation themselves, the practice of daily debriefing did not seem to involve much prospective planning.

One team (1A) held debriefings in conjunction with other activities such as in-service training or medication preparation, which diluted the effects of debriefing. In other teams, feedback was said to happen once a month, but we were unable to validate this during the period of data collection, and it was unlikely the CHWs would be able to discuss visits made a month ago. Those teams that had no place to meet inside the facility tended not to discuss serious matters for reasons of confidentiality. *"Sometimes we stand outside to hold meetings… patients and the security personnel will be listening to what we are discussing." (CHW-FGD, 3A)*

Examination of CHWs' daily logs and registers

The CHWs used notebooks to document their daily activities and record information about patients. Through reviewing these notes, experienced supervisors could discuss the visits with the CHWs, ask questions and provide support, as observed in teams 1A, 1B and 2B. In every team there were CHWs that took poor notes, but this was better contained by supervisors willing to spend time reviewing records, and discussing problems with the relevant CHW. It sent a clear message that documentation of their activities was central to their work.

Assisting in reporting

SNs of two teams (1A & 2B) required CHWs to compile their statistics every Friday, as doing it on a weekly basis (rather than monthly) reduced recall error and thus enhanced quality. One SN verified the weekly report of each CHW against their



For peer review only - http://bmjopen.bmj.com/site/about/guidelines.xhtml

notebooks, correcting mistakes and making sure that the CHWs understood the meaning of each data category and how notes were translated into figures. The practice strengthened the ability of CHWs to record their work, and trained them to be accountable for what they did. To make sure that CHWs reported faithfully, another sophisticated supervisor would make unsolicited household visits, *"Sometimes I would just go out, and when they talk about households, I would say, oh that one who lives in that shack? And they would say, 'Sister you went there?' I would say, yes, it is necessary."(SN, 2B)* In contrast, other supervisors simply received whatever reports the CHW handed over, with no discussion or interrogation of the statistics produced.

Resolving administrative matters

Many of the CHW teams did not have sufficient space to meet, to complete paper work and to store their files; often files were kept at home. One supervisor negotiated a meeting room for her CHWs, which immediately increased their job satisfaction. *"Before she (SN) came, we were like orphans, we had no space inside the clinic. We were doing everything outside. She came with a lot of changes around here…SN had played an important role in our lives…I even enjoy my work now." (CHW-FGD, 1A)* Supervisors in the health post-based SN/JN team (2A) addressed administrative issues, such as leave and absenteeism, at regular morning meetings. The JN supervisor (1B) addressed CHWs' frustration over their employment by suggesting that they prepare a list of issues they wanted to raise in a meeting. She also tried to assist them in their challenges with the payroll administrator. Although challenges remained, the CHWs appreciated the sincerity their supervisor demonstrated.

**The consequences of supervision**


For peer review only - http://bmjopen.bmj.com/site/about/guidelines.xhtml

Performance

The scope of work of the CHWs varied across the teams. CHWs with engaged and skilled supervisors carried out a fuller range of activities, and used their discretion to make decisions, for example: *"A patient had an elevated blood glucose level. However, it was too late for the patient to go to the clinic, so rather than writing a referral letter, she visited the patient again with glucometer after work, by which time the glucose level had dropped by 50%. The next morning, she went for a third time and found that sugar level had returned to normal." (Observation notes, 1A*) This CHW, from a clinic-based SN/JN model, was able to interpret the glucometer readings, and re-checked the patient to avoid making an unnecessary referral.

The dedication of some CHWs to their work is evident in the following interview: *"If you send a message to a CHW, it does not matter what time it is, or what the problem is, they will come and help you. Whatever problem you have, you can talk to them, and you are not worried that they will be talking about your problem around the area. I like the work they do very much." (Patient-BT, 2A)* Another CHW highlighted the effect of work ethic on CHW reputation, *"It's not good if the patient comes here and when one of the clinic staff checks the card finds out the medication delivery is long overdue. It affects all of us in the eyes of the clinic staff and the SN." (CHW-FGD, 1A)* In the JN-only model where supervision was the weakest and support from facility staff was negligible, CHW activities were minimal, with often CHWs simply delivering medication to the elderly, and not reporting situations they encountered, such as suspected TB patients or being denied entry to households.

Motivation, job satisfaction and engagement



For peer review only - http://bmjopen.bmj.com/site/about/guidelines.xhtml

Job satisfaction, professional confidence and motivation were more evident in teams with effective supervision. Two JNs mentored by SNs, explained how they found meaning in their work: *"At first it was challenging for me leaving the hospital and working in the community… maybe I'm not a nurse anymore, but now I feel more useful." (JN, 1B) "I'm a street nurse…but you know what, when I reach the households, this is very enjoyable." (JN, 1A)* One CHW commented that since the SN arrived: *"we started to feel that we are now CHWs and human beings." (CHW-FGD, 1A)* When CHWs were on a strike over issues with their contract in 2016, some of the CHWs at the clinic-based SN/JN site (1B) tried to carry on, not wanting to let their patients down or damage their relationship with supervisors and the facility. Their supervisor interpreted their actions: *"they see that you, as their leader, are supporting them and trying to understand where they come from, especially with this employer thing being mixed up. That is why I am saying, hence, they refused to go on strike today." (JN, 1B)*

However, in the absence of supportive supervision, the frustrations over their working conditions and the system continued, with some CHWs deciding to work fewer hours as a passive protest. Insufficient training constrained CHWs ability to assist patients, and as a result, demotivated them. Teamwork was also affected, as one recalled an incident where most CHWs ignored a patient who came seeking care, *"What hurt me most, was that we cannot look after each other's patients. If I was not around that day, the patient was going to die in front of them (CHWs), because that was a patient of another CHW who was absent." (CHW-FGD, 3A)*

Negotiating acceptance and building the community's trust


For peer review only - http://bmjopen.bmj.com/site/about/guidelines.xhtml

CHWs depended on the supervisor to liaise with key community members: *"We established good relations with the ward councillor so whenever there was a problem I would call her. We had a meeting with the parents at the local school about the programme." (SN, 2B)* The presence of the senior supervisor increased CHWs' credibility in the community: *"If CHWs found that I am not well, they report to the SN, and the SN would come and see me. I have noticed that when I follow the SN's advice, my health improves. We are alive today because of them." (Patient-BT, 2A)*

Support from the facility staff

The resulting collaboration, after the supervisor (1A) successfully arranged a meeting (albeit small) room inside the clinic for her CHWs, had considerable benefits. The facility manager was able to discuss about CHWs' reports presented by the SN, and used the data to confirm whether defaulters returned once they had been traced by CHWs. At site 1B,*"We engage with facility manager, she helps with problems that we have... we include her in everything" (JN, 1B).* Other staff willingly accepted referrals from CHWs, and included the CHWs in training sessions: *"Sister X is not (in the CHW team), but she is very helpful. She attended severe malnutrition training and she called all the CHWs and did a presentation for everybody.*" *(JN, 1B).* In such environments, CHWs were able to negotiate with other less willing staff: *"I have a patient who doesn't want to come to the clinic and I don't want my patient to default on her HIV treatment. I said to the sister 'my patient is working at the farms so please pack medication for her. I'll ask her to come next month.' The sister scolded me because the patient did not come on her last appointment. So I begged the sister and promised to bring the patient myself. Then it was sorted, the patient got her medication and she came to the clinic with me." (CHW-FGD, 1B)*



For peer review only - http://bmjopen.bmj.com/site/about/guidelines.xhtml

Support of clinic staff empowered CHWs to build stronger relationships with their clients. Box 1 recounts the engagement of one CHW with two different households, for which she received very different support from the facility. These two cases illustrate how different levels of support from the clinic profoundly influenced the strength of the relationship with the client. This variation was more prevalent at sites with weaker supervision, as nobody was around to look after the CHWs needs, and by extension those of the patients.

**Box 1: Support from clinic facilitates a CHW to build patient's trust**

Case 1 : A lack of backup from the clinic leads to the client not trusting the CHW

The patient, a woman on ARV with a spinal cord problem, had moved recently. She was no longer able to collect ARVs and go for physio appointment at the clinic near her previous home as she had no money for transport. She asked for a referral to a closer clinic. The nurse refused as ARV patients tended to move clinics, so patient stole the file.

The CHW took her file to the closer clinic to ask if she could collect medication for the patient. The CHW was told that a new file had to be opened and the patient had to wait for CD4 results to come back. As a result, when we interviewed her, the woman had been defaulting for the past 3 weeks.

In the interview the patient said the CHW had not followed up on her requests for food parcels as they had no food, for a physio appointment, or for her husband to be tested for HIV. *"The CHW doesn't follow up on me to see how I am doing; the only thing that she is good at is to make empty promises." (Patient-AA, 3A)*

Case 2: Backup from the social worker leads the client to trust the CHW



For peer review only - http://bmjopen.bmj.com/site/about/guidelines.xhtml

The patient was a woman whose child was knocked down and killed by a car in front of her and her children. The family were traumatised. CHW wrote a referral letter to a social worker. The patient went to the social worker and then started attending a support group. CHW also arranged for the social worker to see her husband and children at home.

Patient said she trusted CHW because *"she is kind, open and she knows how to speak to people even if you are in a difficult situation. She does not gossip about her patients on the street." (Patient-Z, 3A)*

Located at a distance from the main clinics, health post teams received little supervisory support from clinic staff. Other than supplying medication, there was little managerial oversight by the clinic. *"I am not directly supervising them except for sometimes when they are there. You can only supervise something that you can see. With the CHW team it is just via the phone and then it ends there." (Facility Manager, 2A)* At the same time, opportunities for engagement with facility staff were limited. The CHWs experienced greater challenges in facilitating their patients' access to care at the clinic.

Without an SN supervisor to build collaborative relationships, the social distance between the clinic staff and CHWs led to dismissive attitudes towards the CHWs: *"the peer educators, HIV/AIDS counselor we all go together to sign the same contract but they are treated as if they are more educated than us. They call us street maids." (CHW-FGD, 3B)* Even where there were relatively good relationships, facility staff were sometimes insensitive to the challenges the CHWs faced: *"She shouts at us in front of everybody at the clinic. For example she shouted: 'Hey you is that a uniform,*



For peer review only - http://bmjopen.bmj.com/site/about/guidelines.xhtml

*that is not the way we dress here.' That is not okay. Where are we going to get new navy pants that cost R200." (CHW-FGD, 1B)*

Building the next generation of supervisors

The clinic-based JNs in the SN/JN model (1B) and JN-only model (3B) were similar in age and experience, but the outcomes of their work differed. The support and on-the-job coaching from SNs improved JNs' credibility among CHWs. They conducted frontline tasks such as traveling with CHWs, doing health campaigns in the community, and on the days when they stayed in the clinic, they observed how SNs built within-facility relations. The accounts of the mentored JNs redefining their role as a community nurse indicate a commitment to their work. Without SNs to help them bridge the social distance, JNs struggled to manage CHWs, build working relationships with clinic staff, or to liaise with community stakeholders, and their supervision yielded unsatisfactory results.

For peer review only



For peer review only - http://bmjopen.bmj.com/site/about/guidelines.xhtml

## DISCUSSION

In this paper, we explored three different supervision configurations in the CHW programme in Sedibeng District, and described the activities of full-time supervisors and their impact on CHW performance. Our findings show that experienced supervisors employed a range of strategies to monitor CHW performance and improve the quality of their work (including supervised home visits, on-the-job training, frequent debriefing, reviewing daily logs, assisted reporting, and administrative support). Clinic-based teams led by senior nurses were best able to draw on available human and material resources, and were more likely to facilitate an effective referral to the clinic. The teams in stand-alone health posts found it difficult to build relationships with the facility, and the JN-headed teams lacked adequate supervision and consequently showed minimal performance. Supervisors who built skills of the CHW and supported them in their work, including administrative issues, were rewarded with motivated CHW. Consistent findings were presented by another South African research that nurse supervisors were unable to support CHWs in meaningful ways due to their unfamiliarity with community-based work.[5] Supervision is often described by its frequency, but clearly insufficient to explain its outcomes; a recent review indicates that the process and quality of supervision have greater impact than frequency,[21] as suggested by our study.

Previous studies have indicated that if health care workers acknowledge CHWs' contribution, and CHWs and their co-workers work as a team, the likelihood of success of a CHW programme is increased.[18, 23-27] Our research suggests that physical attachment to a facility provides a basis for the initial recognition, but that an able facilitator is required to build relationships between the different cadres. The SN



For peer review only - http://bmjopen.bmj.com/site/about/guidelines.xhtml

was a shared human resource for the CHWs and facility, which placed her in a strategic position to bridge the divide. By securing a place for the CHWs in the facility, the supervisor obtained a position – physical and symbolic – for them in the health system. Facility staff and CHWs had to work alongside each other. Once a reciprocal relationship was established, it fostered a positive cycle that drew in other staff to participate in the supervision of CHWs, further raising the skills of the CHWs and hence their legitimacy among clinic staff. However, although supervision by facility staff may enhance connectedness to the health system and legitimize CHWs' work, facility staff alone are unlikely to have sufficient time to build community relationships, to provide hands-on supervision during household visits, or regular on-the-job training.[19]

While senior nurse supervisors in our study fulfilled the majority of the essential functions, they provided little support in work planning. Prospective planning is considered a supportive practice.[28, 29] CHWs and their supervisors in Tanzania reported that discussing planning was productive as it created opportunities for feedback and support.[19] In our study, we did not observe explicit planning at any site. CHWs had a multitude of tasks, but on most days they worked in a seemingly impromptu manner and did not always make optimal use of their time. We did not observe the supervisors helping to maximize their efficiency.

Our study has several limitations. First, observation of supervisors and CHWs at work was likely to have some influence on their behaviour. Despite asking CHWs to carry out their duties as they normally did, many appeared to make extra effort to reach a greater number of households than they normally did. We followed the same CHWs



For peer review only - http://bmjopen.bmj.com/site/about/guidelines.xhtml

for 3-5 days to mitigate the bias, and the number of household visits did fall, as the CHWs became used to the presence of an observer. Second, it is worth mentioning that we assessed performance based on what we learned from our subjects rather than an objective indicator of outcomes, although our objective was to document as comprehensively as possible in this process evaluation. Third, we purposively selected the sites to ensure different supervisory configurations were included. However, we do not know to what extent the included CHW teams are representative of all the CHW teams in the District. The study demonstrates noteworthy strengths. As far as we know, this is the first study to describe dedicated, full time supervisors' practice in a CHW programme. We collected a substantial amount of qualitative data capturing different stakeholder perspectives, and we used these narratives to represent some of the untold practices of supervision.

**CONCLUSION**

With high expectations of the CHW programme in improving access to care in South Africa and other similar settings, we found that full-time supervision can make an important contribution in enabling CHW programmes to achieve their potential. In the context of an overstretched health system, which struggles to provide necessary resources and to carry out the administrative tasks associated with the CHW programme, supportive supervision can be critical in ensuring that CHWs do not become demotivated with insufficient skills to help their clinics, as a result of being marginalised at the periphery of the system.



For peer review only - http://bmjopen.bmj.com/site/about/guidelines.xhtml

**Contributorship**

TYH interpreted data, wrote and revised the manuscript, and approved the final manuscript as submitted.

GFE and GJ conceptualized and designed the study, analysed data, critically revised the manuscript and approved the final manuscript as submitted.

DKJ carried out data collection process, managed data, analysed data, critically revised the manuscript, and approved the final manuscript as submitted.

NN analysed data, revised the manuscript and approved the final manuscript as submitted.

RT and MH analysed data and approved the final manuscript as submitted.

**Acknowledgements**

The authors would like to acknowledge the following individuals for their invaluable contribution: the field workers, the respondents, the CHW teams, Mrs Salamina Hlahane, district manager at Sedibeng, and Mrs Bridget Lefhoedi, community-based services coordinator. Input from Prof. Margaret Thorogood throughout the study is highly appreciated. We also thank these organizations for their generous support: Department of Health Sedibeng District, Gauteng Department of Health, the UK Medical Research Council (MRC) and Taiwan Ministry of Science and Technology.

**Competing Interest**

None.

**Data sharing**

No additional data available.

**Funding**

MRC-UK under Health Systems Research Initiative (MR/N015908/1)
Taiwan Ministry of Science and Technology (105-2917-I-564-006)



For peer review only - http://bmjopen.bmj.com/site/about/guidelines.xhtml

**REFERENCES**


1. Perry HB, Zulliger R, Rogers MM. Community health workers in low-, middle-, and high-income countries: an overview of their history, recent evolution, and current effectiveness. *Annu Rev Public Health* 2014;35:399-421.
2. Datiko DG, Yassin MA, Theobald SJ, et al. Health extension workers improve tuberculosis case finding and treatment outcome in Ethiopia: a large-scale implementation study. *BMJ Glob Health* 2007;2**:**(4)e000390.
3. Lehmann U, Sanders D. Community health workers: what do we know about them. The state of the evidence on programmes, activities, costs and impact on health outcomes of using community health workers. Geneva: World Health Organization 2007:1–42.
4. Lewin S, Munabi Babigumira S, Glenton C, et al. Lay health workers in primary and community health care for maternal and child health and the management of infectious diseases. *The Cochrane Library* 2010.
5. van Ginneken N, Lewin S, Berridge V. The emergence of community health worker programmes in the late apartheid era in South Africa: An historical analysis. *Soc Sci Med* 2010;71:1110-8.
6. Barnett ML, Gonzalez A, Miranda J, Chavira DA, Lau AS. Mobilizing Community Health Workers to Address Mental Health Disparities for Underserved Populations: A Systematic Review. *Adm Policy Ment Health* 2017;1-17.
7. Marquez L, Kean L. Making supervision supportive and sustainable: new approaches to old problems. Paper no.4. Suplement to Population Reports, Vol.XXX, Maximizing Access and Quality (MAQ) Initiative. Washington, DC: Management Sciences for Health 2002:1-28.
8. Greenspan JA, McMahon SA, Chebet JJ, et al. Sources of community health worker motivation: a qualitative study in Morogoro Region, Tanzania. *Hum Resour Health* 2013;11:52.
9. Strachan DL, Källander K, ten Asbroek AH, et al. Interventions to improve motivation and retention of community health workers delivering integrated community case management (iCCM): stakeholder perceptions and priorities. *Am J Trop Med Hyg* 2012;87(Suppl 5):111-9.
10. Campbell C, Scott K. Retreat from Alma Ata? The WHO's report on task shifting to community health workers for AIDS care in poor countries. *Glob Public Health* 2011;6:125-38.
11. Celletti F, Wright A, Palen J, et al. Can the deployment of community health workers for the delivery of HIV services represent an effective and sustainable response to health workforce shortages? Results of a multicountry study. *AIDS* 2010;24:S45-S57.
12. Dawson P, Pradhan Y, Houston R, et al. From research to national expansion: 20 years' experience of community-based management of childhood pneumonia in Nepal. *Bull World Health Organ* 2008;86:339-43.
13. Rowe AK, de Savigny D, Lanata CF, et al. How can we achieve and maintain high-quality performance of health workers in low-resource settings? *Lancet* 2005;366:1026-35.




For peer review only - http://bmjopen.bmj.com/site/about/guidelines.xhtml

14. Pallas SW, Minhas D, Pérez-Escamilla R, et al. Community health workers in low-and middle-income countries: what do we know about scaling up and sustainability? *Am J Public Health* 2013;103:e74-e82.
15. Liu A, Sullivan S, Khan M, et al. Community health workers in global health: scale and scalability. *Mt Sinai J Med* 2011;78:419-35.
16. Baatiema L, Sumah AM, Tang PN, et al. Community health workers in Ghana: the need for greater policy attention. *BMJ Glob Health* 2016;1:e000141.
17. Jaskiewicz W, Tulenko K. Increasing community health worker productivity and effectiveness: a review of the influence of the work environment. *Hum Resour Health* 2012;10:38.
18. Kok MC, Dieleman M, Taegtmeyer M, et al. Which intervention design factors influence performance of community health workers in low-and middle-income countries? A systematic review. *Health Policy Plan* 2014;30:1207-27.
19. Roberton T, Applegate J, Lefevre AE, et al. Initial experiences and innovations in supervising community health workers for maternal, newborn, and child health in Morogoro region, Tanzania. *Hum Resour Health* 2015;13:19.
20. Das A, Friedman J, Kandpal E, et al. Strengthening malaria service delivery through supportive supervision and community mobilization in an endemic Indian setting: an evaluation of nested delivery models. *Malar J* 2014;13:482.
21. Hill Z, Dumbaugh M, Benton L, et al. Supervising community health workers in low-income countries–a review of impact and implementation issues. *Glob Health Action* 2014;7:24085.
22. Nxumalo N, Choonara S. A rapid assessment of ward-based PHC outreach teams in Gauteng Sedibeng district–emfuleni sub-district. Johannesburg: Centre for Health Policy 2014:1-30.
23. Gilson L, Walt G, Heggenhougen K, et al. National community health worker programs: how can they be strengthened? *J Public Health Policy* 1989;10:518-32.
24. Lehmann U, Friedman I, Sanders D. Review of the utilisation and effectiveness of community-based health workers in Africa. Global Health Trust, Joint Learning Initiative on Human Resources for Health and Development (JLI), JLI Working Paper 4-1. 2004.
25. Haines A, Sanders D, Lehmann U, et al. Achieving child survival goals: potential contribution of community health workers. *Lancet* 2007;369:2121-31.
26. Chang LW, Kagaayi J, Arem H, et al. Impact of a mHealth intervention for peer health workers on AIDS care in rural Uganda: a mixed methods evaluation of a cluster-randomized trial. *AIDS Behav* 2011;15:1776.
27. Teela KC, Mullany LC, Lee CI, et al. Community-based delivery of maternal care in conflict-affected areas of eastern Burma: perspectives from lay maternal health workers. *Soc Sci Med* 2009;68:1332-40.



For peer review only - http://bmjopen.bmj.com/site/about/guidelines.xhtml

28. Djibuti M, Gotsadze G, Zoidze A, et al. The role of supportive supervision on immunization program outcome-a randomized field trial from Georgia. *BMC Int Health Hum Rights* 2009;9:S11.
29. PATH. Guidelines for implementing supportive supervision: a step-by-step guide with tools to support immunization. http://www.path.org/publications/detail.php?i=1212 (accessed 20 Sep 2017)

For peer review only



For peer review only - http://bmjopen.bmj.com/site/about/guidelines.xhtml

Consolidated criteria for reporting qualitative studies (COREQ): 32-item checklist

| Item | | **Guide questions/description** | |
|---|---|---|---|
| **Domain 1: Research team and reflexivity** | | | |
| **Personal Characteristics** | | | |
| 1. | Interviewer/facilitator | Which author/s conducted the interview or focus group? | **Field workers trained by researchers collected data** |
| 2. | Credentials | What were the researcher's credentials? *E.g. PhD, MD* | **PhD, MD, MS and MA** |
| 3. | Occupation | What was their occupation at the time of the study? | **Professors, researchers, postdocs, and PhD fellows** |
| 4. | Gender | Was the researcher male or female? | **6 females + 1male** |
| 5. | Experience and training | What experience or training did the researcher have? | **Health policy, qualitative, health systems research, social work** |
| Relationship with participants | | | |
| 6. | Relationship established | Was a relationship established prior to study commencement? | **No** |
| 7. | Participant knowledge of the interviewer | What did the participants know about the researcher? e.g. *personal goals, reasons for doing the research* | **Researchers and fieldworkers explained to participants the reasons for doing the research** |
| 8. | Interviewer characteristics | What characteristics were reported about the interviewer/facilitator? e.g. *Bias, assumptions, reasons and interests in the research topic* | **Researchers explained the aims and objectives of the study to the participants.** |
| **Domain 2: study design** | | | |
| Theoretical framework | | | |
| 9. | Methodological orientation and Theory | What methodological orientation was stated to underpin the study? *e.g. grounded theory, discourse analysis, ethnography, phenomenology, content analysis* | **Content analysis** |
| Participant selection | | | |
| 10. | Sampling | How were participants selected? *e.g. purposive, convenience, consecutive, snowball* | **Purposive** |
| 11. | Method of approach | How were participants approached? e.g. *face-to-face, telephone, mail, email* | **Either telephonically, or face-to-face.** |
| 12. | Sample size | How many participants were in the study? | **201 individuals were being observed or/and interviewed.** |
| 13. | Non-participation | How many people refused to participate or dropped out? Reasons? | **Only one senior nurse supervisor. However, she didn't really refuse, but only rescheduled a number of times and we eventually took it as a reluctance to participate.** |
| Setting | | | |
| 14. | Setting of data collection | Where was the data collected? e.g. *home, clinic, workplace* | **Home for household members; workplace (incl. community and facility) for CHWs and supervisors** |

For peer review only

| | | | |
|---|---|---|---|
| 15. | Presence of non-participants | Was anyone else present besides the participants and researchers? | **Yes, during observations, where there were community members and clinic staff.**<br>**No, during focus group & interview.** |
| 16. | Description of sample | What are the important characteristics of the sample? *e.g. demographic data, date* | **Characteristics of the sample related to research topic are provided in the first part of Results, page 11.** |
| Data collection | | | |
| 17. | Interview guide | Were questions, prompts, guides provided by the authors? Was it pilot tested? | **Yes.**<br>**Pilot tested between researchers and fieldworkers (facilitators).** |
| 18. | Repeat interviews | Were repeat interviews carried out? If yes, how many? | **Follow-up focus group discussions at 4 of the clinic-based CHWs** |
| 19. | Audio/visual recording | Did the research use audio or visual recording to collect the data? | **Interviews and focus discussions were audio recorded for transcription, as they were done in local language.** |
| 20. | Field notes | Were field notes made during and/or after the interview or focus group? | **Yes, notes were made for every piece of data, both during data collection and afterwards.** |
| 21. | Duration | What was the duration of the interviews or focus group? | **5 months** |
| 22. | Data saturation | Was data saturation discussed? | **Yes** |
| 23. | Transcripts returned | Were transcripts returned to participants for comment and/or correction? | **No** |
| **Domain 3: analysis and findings** | | | |
| Data analysis | | | |
| 24. | Number of data coders | How many data coders coded the data? | **All 7 authors** |
| 25. | Description of the coding tree | Did authors provide a description of the coding tree? | **No** |
| 26. | Derivation of themes | Were themes identified in advance or derived from the data? | **Both** |
| 27. | Software | What software, if applicable, was used to manage the data? | **Done manually** |
| 28. | Participant checking | Did participants provide feedback on the findings? | **Yes, participants were invited to a workshop where researchers presented preliminary findings and received participant feedback.** |
| Reporting | | | |
| 29. | Quotations presented | Were participant quotations presented to illustrate the themes / findings? Was each quotation identified? e.g. *participant number* | **Yes** |
| 30. | Data and findings consistent | Was there consistency between the data presented and the findings? | **Yes** |
| 31. | Clarity of major themes | Were major themes clearly presented in the findings? | **Yes** |

For peer review only

| | | | |
|---|---|---|---|
| 32. | Clarity of minor themes | Is there a description of diverse cases or discussion of minor themes? | **Yes** |

For peer review only

# Integrating community health workers into the formal health system to improve performance: the role of on-site supervision in the South African CHW programme

| | |
|---|---|
| Journal: | *BMJ Open* |
| Manuscript ID | bmjopen-2018-022186.R1 |
| Article Type: | Research |
| Date Submitted by the Author: | 25-Jun-2018 |
| Complete List of Authors: | Tseng, Yu-hwei; University of the Witwatersrand School of Public Health, Centre for Health Policy<br>Griffiths, Frances; University of Warwick Warwick Medical School<br>de Kadt, Julia; University of the Witwatersrand School of Public Health, Centre for Health Policy Johannesburg, Gauteng, ZA<br>Nxumalo, Nonhlanhla; University of the Witwatersrand School of Public Health, Centre for Health Policy<br>Rwafa, Teurai; University of the Witwatersrand School of Public Health, Centre for Health Policy<br>Malatji, Hlologelo; University of the Witwatersrand School of Public Health, Centre for Health Policy<br>Goudge, Jane; University of the Witwatersrand School of Public Health, Centre for Health Policy |
| <b>Primary Subject Heading</b>: | Health services research |
| Secondary Subject Heading: | Qualitative research, Public health, Health policy |
| Keywords: | Human resource management < HEALTH SERVICES ADMINISTRATION & MANAGEMENT, Organisation of health services < HEALTH SERVICES ADMINISTRATION & MANAGEMENT, QUALITATIVE RESEARCH, PUBLIC HEALTH, Health policy < HEALTH SERVICES ADMINISTRATION & MANAGEMENT |
| | |

SCHOLARONE™
Manuscripts

# Integrating community health workers into the formal health system to improve performance: the role of on-site supervision in the South African CHW programme

Yu-hwei Tseng,[1] Frances E Griffiths,[1,2] Julia De Kadt,[1] Nonhlanhla Nxumalo,[1] Teurai Rwafa,[1] Hlologelo Malatji,[1] Jane Goudge[1],

**Affiliations**
[1] Centre for Health Policy, School of Public Health, University of the Witwatersrand, Johannesburg, South Africa;
[2]Division of Health Sciences, Warwick Medical School, University of Warwick, Coventry, United Kingdom

**Address correspondence to:**
Jane Goudge
Centre for Health Policy (CHP)
School of Public Health, Faculty of Health Sciences, University of the Witwatersrand
Private Bag X3 Wits 2050, Johannesburg, South Africa
Tel: +27 11 717 3425; Email: jane.goudge@gmail.com

**Keywords:** community health worker, supervision, South Africa, health system integration, health post

**Abstract word count: 300**

**Text word count: 5,595**

**Table: 5**

**Box: 1**

**Number of references: 29**



For peer review only - http://bmjopen.bmj.com/site/about/guidelines.xhtml

## ABSTRACT

### INTRODUCTION

Community health worker (CHW) programmes have the potential to improve access to primary health care and so assist in achieving universal health coverage. However, their effectiveness is often limited by inadequate supervision, and insufficient integration into the health system. South Africa is implementing a national CHW programme with on-site supervisors. In the observation study we compare the functioning of CHW teams reporting to a clinic-based nurse with teams based in the community with a nurse who doesn't have additional clinic duties. We also consider whether a junior nurse can provide adequate supervision, given the shortage of senior nurses.

### METHODS

In the initial observation phase of a 3-year intervention study, we studied six CHW teams with different configurations of supervision and location: either a junior nurse, or a junior with a senior nurse, and either facility- or community-based. We observed activities of CHWs and their supervisors (126 person-days), conducted focus group discussions (12), key informant interviews (43), and interviews with clients (74).

### RESULTS

Effective supervisors supported CHWs through household visits, on-the-job training, debriefing, review of CHWs' daily logs, and assistance with reporting. CHWs led by senior nurses were motivated and performed a greater range of tasks; junior nurses in these teams could better fulfil their role. Clinic-based teams with senior supervisors were better integrated into the health system and more able to ensure continuity of care. In contrast, teams with only junior supervision, or based in the community, had less engagement with the clinic staff, were less able to ensure necessary care for patients, resulting in less trust with their clients.

### CONCLUSION

Senior supervisors successfully negotiated a place for CHWs in the health system. Collaboration with clinic staff reduced the CHW's marginalisation, and enabled their contribution. Despite being clinic-based, teams without senior supervision were not integrated into the health system.




For peer review only - http://bmjopen.bmj.com/site/about/guidelines.xhtml

**Strengths and limitations of this study**

- To the best of our knowledge, this is the first study that examined the role of different levels of supervision and the location of the CHW team on the team's motivation, performance and integration into the health system.
- The case study approach allowed us to purposively select teams based on their different supervision/ location configurations, and to compare and contrast their functioning to understand what worked, and what did not.
- We employed multiple qualitative methods, including observation, interview and focus groups, to collect a substantial amount of data, enabling triangulation across data sources and sites.
- Limitations to the study include the observer effect during data collection, subjective rather than objective evaluations of CHW performance, and participants that were not representative of the population.

For peer review only



For peer review only - http://bmjopen.bmj.com/site/about/guidelines.xhtml

## INTRODUCTION

Community health workers (CHWs) play an important role in improving health care coverage and access in low and middle-income countries (LMICs).[1-3] However, optimizing the contribution of CHWs continues to be a global challenge. CHWs, with no professional qualification, perform primary health care (PHC) related functions in the community.[1, 4] They work on the periphery of the health system and often in under-served communities where needs are immense and multifaceted. For this reason, CHWs require frequent and supportive supervision that supplements their training and connects them with the health system.[3, 5, 6] Recent reviews suggest that adequate supervision, and integration with the health system, are essential components for programme effectiveness. [5, 7-9]

Effective integration into the health system requires government financing with a national training programme, and integration of the CHW scope of work, supervision, referral networks and supply chain into the hierarchy of the health system.[9] Without integration, there is unlikely to be a smooth patient pathway from CHW to clinic, limiting the effectiveness of the CHWs. There is a growing literature on supervision, with some evidence that supportive supervision improves motivation and performance. [10-14] However much of this evidence is from NGO



For peer review only - http://bmjopen.bmj.com/site/about/guidelines.xhtml

programmes (which are less integrated into the health system), or programmes in which the CHWs have a specific disease (e.g. HIV) or population group focus (e.g. maternal). With the focus on universal access to comprehensive, integrated care, it is important to understand the type and level of supervision that will enable CHWs to provide the full range of required tasks, as well as facilitate integration with the health system.[12-15]

Given the double burden of non-communicable and infectious diseases in LMICs, governments such as South Africa's are seeking to shift CHW initiatives from focusing on a single disease or population group, to a more comprehensive approach.[16] South Africa is implementing a national CHW programme (WBOTs; ward-based outreach teams) under a policy to strengthen primary health care [17]. The intention is to provide full-time on-site supervisors whose responsibility is to manage, train, mentor, monitor CHWs and to facilitate links with the health system and the community. However, given the shortage of health care workers, as in other LMIC settings, there is a limited number of nurses able to supervise CHWs. In this study, we ask whether CHW teams should be attached to a clinic and report to a clinic-based nurse who can facilitate referrals and access to supplies, and the CHWs' integration into the health system? Or should they be based in the



For peer review only - http://bmjopen.bmj.com/site/about/guidelines.xhtml

community in which they are working, with a dedicated nurse who doesn't have the additional demands of clinic duties? We also ask whether a junior (enrolled) nurse is able to provide adequate supervision, given the shortage of senior (professional) nurses.

In the initial observation phase (Sept 2016- Feb 2017) of a 3-year intervention study in Sedibeng Health District, we studied CHW teams with different configurations of supervisors and locations: 1) clinic-based teams supervised by a senior (professional nurse; PN) and a junior (enrolled nurse; EN); 2) health post (community)-based teams supervised by a PN and an EN; and, 3) clinic-based teams supervised by an EN only. In this paper, we report on the supervision practices and the team's integration with the health system, and its effect on the CHWs' motivation and performance.

**METHODS**

We used a case study approach to examine the operation of six CHW teams, with each team and their supervisors as a single case study.

**Study site**



For peer review only - http://bmjopen.bmj.com/site/about/guidelines.xhtml

In some South African provinces, CHWs, previously employed by non-governmental organizations (NGOs), have been absorbed into a government programme (called WBOTS; ward-based outreach teams) in an attempt to shift from a patchwork of services to a programme with a national scope.[15, 18] The CHWs' role is predominately health promotion, prevention, screening and referral during household visits. Standardized CHW training covers identification of the need for ante-natal and post-natal care, monitoring immunisation of under 5s, adherence among patients with chronic diseases, screening for malnutrition, TB, gendered based violence, and making resulting referrals to health, social and other services. WBOT teams are meant to be comprised of a professional nurse, six or more CHWs, one health promoter and one environmental officer.[17]

We selected Sedibeng health district (Gauteng Province) as our study site due to the variation in supervision and location of its CHW teams. The district is relatively affluent by South African standards, although over 20% of its residents fall below the food poverty line. Outside the urban areas, disadvantaged communities, with inadequate shelter, food insecurity and high disease burdens, have limited access to services such as clinics, transport, water and electricity.



For peer review only - http://bmjopen.bmj.com/site/about/guidelines.xhtml

Sedibeng District has established 16 health posts to provide community-orientated care, rather than the disease focused care provided by 28 primary health care clinics.[19] Some health posts were placed at some distance from a clinic, in order to improve access to basic services. A health post consists of one or two temporary structures (providing 3-6 rooms), without electricity and often with irregular water supply. It is managed by one or two professional nurses, who obtain medication and other resources via a 'mother' clinic. The nurses supervise the CHW team, and provide basic services such as chronic medication, immunisation and treating minor ailments.

At the time of the study there were 39 CHW teams (each with between 6-20 CHWs) in 37 of the district's 72 wards (smallest geopolitical area). Sixteen of the teams were based at a health post and the remaining 23 were clinic-based. In addition to the services outlined in the national training programme (see above), the CHWs delivered medication to elderly or disabled chronically ill patients.

**Selection of CHW teams**

The research team consulted with district officials, categorizing the CHW teams into three types (See Table 1). We selected two teams of each type with the requirement



For peer review only - http://bmjopen.bmj.com/site/about/guidelines.xhtml

that each pair of teams needed to be as similar as possible (important characteristics included urban or rural location, the size of the teams, and the type of community they served). (We needed to pool the data from the two teams to generate a sufficient sample for the analysis of the coverage data, reported elsewhere). Tables 1 and 3 describe the characteristics of different teams and their catchment areas. The two health post teams were closest to the community, while the rural teams had the largest geographical area to cover (Table 1).

**Table 1. Supervision configurations of CHW teams and their catchment areas**

| Type | Supervisor | Based in | Site Code | Description of urban/rural location and type of community served | Max. distance CHW base to furthest household |
|---|---|---|---|---|---|
| 1 | Professional & Enrolled nurse | Clinic | 1A | Township[1] with relatively well-off households and apartheid-era housing | 2 km |
| | | | 1B | Township with RDP[2] housing and informal settlement[3] | 3 km |
| 2 | Professional & Enrolled nurse | Health post | 2A | Densely populated semi-urban area | 1 km |
| | | | 2B | Large informal settlement, with many migrants | 1 km |
| 3 | Enrolled nurse only | Clinic | 3A | Rural, informal settlement, also with RDP housing and farm plots | 6 km |
| | | | 3B | Rural, township and farm plots | 25 km |

[1] Townships = urban areas built on the peripheries of towns and cities; [2]RDP housing= low-cost government built housing; [3] informal settlements=where impoverished population built shacks on vacant land.

## Data collection

Eight fieldworkers received two weeks of training, covering research methods and ethics, study tools, extensive role-play and observation practice, as well as an overview of training that CHWs receive. Between Sept 2016 and Feb 2017, the field



For peer review only - http://bmjopen.bmj.com/site/about/guidelines.xhtml

team collected interview and observation data on each teams' activities, resources, engagement with the clinic and community, successes and challenges, as well as respondent's views of the performance of the teams. (Further details of CHW performance using more objective indicators, such as coverage and the quality of care, will be reported elsewhere.) Data collection method and types of data are summarised in Table 2

**Table 2: Data collection method, participants and data collected**

| Method | Participants | Total number in 6 sites | Data collected |
|---|---|---|---|
| Observation | CHWs and supervisors while conducting their daily work | 126 days of observation | Descriptions of activities and interactions; descriptions of clients encountered; |
| FGD[1] | CHW teams | 12 FGDs (76 participants) | FGD: descriptions of activities and weekly & daily routines; resources available and needed; support from supervisors, peers, clinic and community; employment conditions; challenges of the programme<br><br>Self-administered questionnaire: age, years of training and service, |
| Interviews | Supervisors and facility managers<br><br>Community representatives | 43 key informant interviews | Background, training responsibilities, weekly & daily pattern of CHWs; resources; successes and challenges<br><br>Perceptions of the programme; acceptability to the community needs; |
| | Follow-up interviews with CHWs' clients who were referred to the clinic during observations of household visits | 74 household interviews | Client's perception of the service, and events subsequent to the referral |

[1] FGD: focus group discussion.

Fieldworkers observed CHWs and their supervisors throughout the workday, and wrote detailed daily field notes guided by a template. After each day spent in the field, the fieldworkers spent a day in the office to type up field notes in full. Each CHW or supervisor was observed for 3-5 consecutive days (interrupted by the office days) to allow for reduction in the Hawthorne effect. Participants were randomly



For peer review only
For peer review only - http://bmjopen.bmj.com/site/about/guidelines.xhtml

selected, although changes were sometimes required due to CHWs taking leave or being absent. Participants responded well to observation, and relaxed substantially during the course of the first day of observation, although there was some evidence the CHWs conducted more household visits early on in the observations.

All available CHWs participated in the FGDs, and all supervisors and facility managers (except one) were interviewed. Community representatives were purposively sampled.

Finally, 74 household members, who received referral advice from CHWs during observed home visits, were interviewed a month later, to understand their experience of the CHW service, whether referral advice was acted upon, and whether the follow up service was effective. Interviews were conducted in participant homes, in the participant's choice of language(s), and recorded with a digital recorder. Field workers used the recording to draft a summary of the interview in English.

**Data analysis**

Taking a case study approach, we drew data from the various sources to develop an explanatory description of each team, and then drew comparisons across teams.



For peer review only - http://bmjopen.bmj.com/site/about/guidelines.xhtml

This process involved several steps. Firstly, we extracted data from each interview or day's observation into a template, either by summarising descriptions of events, or extracting raw data such as useful quotations. This increased our familiarity with the data and allowed us to reduce its volume significantly. Initially, multiple team members extracted data from same sources, compared extracted data and modified extraction strategies until we were confident about inter-extractor reliability.

Secondly, the authors presented a brief summary of each site in a one-day workshop. Through this collective process we identified themes that revealed the similarities and differences across the sites, such as weekly and daily pattern of activities, resources available, record keeping, managing patient referrals to the clinic, engagement with clinic staff, relationship between CHWs and supervisors, relationship with patients, local NGOs, and key community stakeholders. We then generated a template into which we collated site-specific data under these themes, producing a summary for each site (see example in appendix).

In preparing the manuscript, we continued to revisit the raw and summarised data to confirm descriptions of the context, enrich the content, provide clarity, and check emergent ideas, throughout data interpretation and manuscript writing. The



For peer review only - http://bmjopen.bmj.com/site/about/guidelines.xhtml

multiple data sources, as well as events documented at times by multiple field workers, allowed triangulation that increased the validity of our findings.

**Ethics**

The study protocol and instruments were approved by the University of the Witwatersrand's Human Research Ethics Committee (Medical) (M160354), the Gauteng Provincial Health Research Committee in South Africa, and the Biomedical & Scientific Research Ethics Committee (BSREC) at Warwick University (REGO-2016- 1825), United Kingdom. All participants gave informed consent. When accompanying CHWs into the community, fieldworkers obtained verbal consent from household members, prior to entering any household. There were few refusals.

**Patient and Public Involvement**

Patients were not involved other than as respondents. Feedback has been given to provincial, district and facility management, as well as CHW supervisors and CHWs in the intervention sites in various forms, including facility level workshops.



For peer review only - http://bmjopen.bmj.com/site/about/guidelines.xhtml

## RESULTS

<u>CHW *conditions of work across districts*</u>

The CHWs are not formally employed by the government, but are an outsourced workforce, managed by a private payroll company, and paid a minimal stipend for 6 hours work a day. CHWs without a high school diploma voiced their fears about lack of job security. The opportunities for pursuing a career in nursing are limited. CHWs struggled to contact the private payroll company responsible for monitoring attendance and paying stipends. An electronic system requiring CHWs to clock in/out at the clinic limited their ability to reach outlying areas as they needed to travel between the clinic and these areas on foot. Two months prior to the start of our fieldwork CHWs had been on strike over their conditions of employment.

The CHWs often worked without necessary resources, including equipment, stationery, uniform, badges, or funds for transport or communication. For example, a team of 20 CHWs shared 1 or 2 blood pressure machines borrowed from the facility. When equipment, such as a glucometer, was provided, the associated consumables such as glucose strips were often not replenished. Notebooks CHWs use to record daily activities were purchased out of their own stipend. The general lack of resources demotivated the CHWs and generated resentment. While teams



For peer review only - http://bmjopen.bmj.com/site/about/guidelines.xhtml

based in health posts had some dedicated space, clinic-based teams did not have sufficient space to meet, to complete paper work and to store their files; often files were kept at home. One supervisor negotiated with the facility manager to use a meeting room for CHW meetings, which immediately increased their job satisfaction.

*"Before she (PN) came, we were like orphans, we had no space inside the clinic. We were doing everything outside. She came with a lot of changes around here…PN had played an important role in our lives…I even enjoy my work now." (CHW-FGD, 1A)*

**The supervisors**

The supervisors in our study sites were either senior (ie. professional) or junior (ie. enrolled) nurses. A professional nurse (PN), with a 4-year degree in nursing, is able to diagnose patients, prescribe treatment and dispense medication. More importantly, the PN supervisors in the study site were also trained in primary health care and community nursing, and had attended various other courses on TB, HIV, diabetes and hypertension, integrated management of childhood illnesses (IMCI), nursing management and leadership. Many were rehired retirees[1], who had more



For peer review only - http://bmjopen.bmj.com/site/about/guidelines.xhtml

For peer review only

than 30 years of experience as a nurse before they joined the programme (Table 3). Enrolled nurses (EN) have completed a 2 year course and can provide nursing care under supervision of a PN. All ENs except one (site 3B) received 1-week induction training on the WBOT programme before joining. Some of the EN supervisors in the site had also attended courses on early childhood development (ECD), TB, prevention of mother to child transmission (PMCT), and the expanded immunisation programme (EPI). Most of the ENs were younger and less experienced in community work than the CHWs they supervised. The PN supervisors reported to the facility manager, as did the EN supervisors, if there was no PN supervisor in the team.

**Table 3. Supervision of CHW – who, what and when**

| SUPERVISION AND LOCATION OF TEAMS | PN / EN clinic-based | | PN / EN health post-based | | EN clinic-based | |
|---|---|---|---|---|---|---|
| | 1A | 1B | 2A | 2B | 3A | 3B |
| <u>**Who supervises and is supervised**</u> | | | | | | |
| **PN supervisor** | | | | | | |
| Age (years) | 65 | 72 | 66 | 59 | n.a. | n.a. |
| Years as nurse | 36 | >30 | 38 | 39 | n.a. | n.a. |
| Years in programme | 4 | 4 | 4 | 4 | n.a. | n.a. |
| **EN supervisor** | | | | | | |
| Number of JN in team | 2 | 2 | 1 | 1 | 1 | 1 |
| Mean age (years) | 43 | 29 | 28 | 25 | 36 | 31 |
| Mean years as nurse | 14 | 2.5 | 0.5 | 3 | 5 | 2 |
| Years in programme | 0.6 | 0.6 | 0.6 | 0.6 | 0.3 | 0.3 |
| **CHW** (supervisee) | | | | | | |
| Number of CHW in team | 16 | 17 | 9 | 12 | 14 | 20 |
| Mean age in years (range) | 38 (26-53) | 39 (25-47) | 34 (27-45) | 37 (26-51) | 42 (23-58) | 33 (23-54) |
| Mean years (range) as CHW | 7 (4-12) | 7 (3-16) | 7 (6-9) | 6 (3-12) | 10 (3-9) | 6 (5-17) |
| **Formal training of CHWs** | | | | | | |
| % CHW received phase 1 training | 91% | 93% | 100% | 90% | 5.6% | 16.7% |
| % CHW received phase 2 training | 91% | 21% | 100% | 46% | 0% | 0% |
| <u>**Supervision activities**</u> | | | | | | |
| **Supervised home visits (day/week)** | | | | | | |
| By professional nurse | n.o. | n.o. | 1 | Occasional | n.a. | n.a. |
| By enrolled nurse | 3 | 4-5 | 4-5 | s.s. | 4 | On request |



For peer review only - http://bmjopen.bmj.com/site/about/guidelines.xhtml

| SUPERVISION AND LOCATION OF TEAMS | PN / EN clinic-based | | PN / EN health post-based | | EN clinic-based | |
|---|---|---|---|---|---|---|
| | 1A | 1B | 2A | 2B | 3A | 3B |
| **On-the-job training** | Yes | Yes | Yes | Yes | n.o. | s.s. |
| **Regular debriefing** | Daily | n.o. | Daily | Weekly | s.s. | s.s. |
| **Examining daily logs and registers** | Yes | Yes | n.o. | Yes | n.o. | n.o. |
| **Assisting in reporting** | Yes | Yes | n.o. | Yes | n.o. | n.o. |
| Frequency of preparing reports | Weekly | Monthly | Monthly | Weekly | Weekly | Monthly |
| **Resolving administrative matters** | Yes | Yes | Yes | n.o. | n.o. | n.o. |

Notes: n.a.: not applicable; n.o.: not observed & not indicated in other information sources; s.s.: the activity was mentioned by a single source other than CHWs, but was not indicated in other data sources.

## Supervisor activities

*Formal and informal (on-the-job) training*

In four of the six sites, 90~100% of the CHWs had completed the first phase of the national training programme, but only 3 sites (all with PN supervisors) had a significant number of CHWs who had passed the second phase of training (Table 2). Both phases require a written test, followed by a practical assessment conducted by the supervisor during household visits; success in this assessment requires a competent supervisor, and comprehensive and on-going training. One PN placed considerable emphasis on training. *"When PN came, she checked who had done what course and organized training for those who needed it."* (*CHW-FGD, 1A*). This PN also used daily morning meetings as training sessions, *"our PN is teaching us … every day in the morning." (CHW-FGD, 1A)*. Given the volume of information that CHWs were required to retain, these regular sessions were important. This PN involved the two ENs in the training: *"Sister teaches us like nobody's business" (EN, 1A)*, enabling the ENs to take up the supervisory role.



For peer review only - http://bmjopen.bmj.com/site/about/guidelines.xhtml

In contrast, in the EN-only sites, supervisors were unable to arrange formal training for the CHWs, in part due to their rural location at some distance from the district training centre, and because the ENs were not sufficiently skilled to carry out the practical assessment. With little experience themselves, they were not able to provide on-the-job training.

*Supervised home visits*

ENs routinely accompanied different pairs of CHWs on home visits several days a week. Some ENs demonstrated sensitivity in correcting CHWs' practice. *"When I can see this one didn't do it right, I keep quiet in the house, but immediately after we step outside as we are walking I do on-the-spot training" (EN, 1B)*. Other ENs working without a PN supervisor, seemed uninvolved during the home visits. Despite one EN spending 4 days a week out in the community, the CHWs did not acknowledge her as a supervisor, *"When we get there she's doing the same job that I normally do when I am alone." (CHW-FGD, 3A)* Consequently, her CHWs didn't report problems they encountered, such as suspected TB patients, as they had little trust in her problem solving capability.

*Regular debriefing*



For peer review only - http://bmjopen.bmj.com/site/about/guidelines.xhtml

One health post-based PN/EN team (2A) held daily debriefing sessions, at which CHWs described the households visited, problems encountered and actions taken. The sessions strengthened CHW's knowledge base and problem-solving abilities, and provided a platform for collective supervision, enabling the CHWs to learn from each other's experience.

One team (1A) held debriefings in conjunction with other activities such as in-service training or medication preparation, which diluted the effects of debriefing. In other teams, feedback was said to happen once a month, but we were unable to validate this, and it was unlikely the CHWs would be able to recall visits made a month ago. Those teams that had no place to meet inside the facility tended not to discuss serious matters for reasons of confidentiality. *"Sometimes we stand outside to hold meetings… patients and the security personnel will be listening to what we are discussing." (CHW-FGD, 3A)*

The CHWs used notebooks to document their daily activities. Through reviewing these notes, experienced supervisors could discuss the visits with the CHWs, ask questions and provide support, as observed in teams 1A, 1B and 2B. In every team there were CHWs that took poor notes, but this was better contained by supervisors



For peer review only - http://bmjopen.bmj.com/site/about/guidelines.xhtml

willing to spend time reviewing records, and discussing problems with the relevant CHWs. It sent a clear message that documentation of activities was central to the work.

*Assisting in reporting*

PNs of two teams (1A & 2B) required CHWs to compile their statistics every Friday, as doing it on a weekly basis (rather than monthly) reduced recall error and thus enhanced quality. One PN verified the weekly report of each CHW against their notebooks, correcting mistakes and making sure that the CHWs understood the meaning of each data category and how notes were translated into figures. This practice strengthened the ability of CHWs to record their work, and trained them to be accountable for what they did. To make sure that CHWs reported faithfully, another supervisor would make unsolicited household visits, *"Sometimes I would just go out, and when they talk about households, I would say, oh that one who lives in that shack? And they would say, 'Sister you went there?' I would say, yes, it is necessary."(SN, 2B)* In contrast, EN supervisors without senior support, tended to simply received whatever reports the CHWs handed over, with no discussion or interrogation of the statistics produced.



For peer review only - http://bmjopen.bmj.com/site/about/guidelines.xhtml

<u>*Clinic duties*</u>

There was an understanding between district officials and facility staff that clinic-based supervisors were to spend 70% of their time on CHW duties, and 30% of their time they could assist clinic staff, particularly if the clinic was short staffed. District officials regularly restated this 'rule' as they were concerned about supervisors neglecting their CHW duties. We did observe EN supervisors, who didn't have PN supervisors, spending the majority of their time in the clinic, but this was because they felt unable to contribute to CHW activities. However, one EN supervisor with PN mentor said:

*"if I am not busy I help with vital signs stations.. it is not part of my job but I do it anyway .. I refer my patients and the nurses see them. So there is teamwork. The support system is great so I never felt pressure to leave my WBOT duties"* (EN interviews, 1B)

*"Remember, (patients) in this family planning (unit of the clinic), are also the children of the mothers and the fathers that we deliver the medication at home. So, we're working hand in hand….even if they (clinic staff) ask, are you finished what you were doing? If you don't mind, can you please help me to take a blood pressure on this patient? Then I will do.*" (EN interview, 1A)



For peer review only - http://bmjopen.bmj.com/site/about/guidelines.xhtml

**The consequences of supervision**

<u>*Motivation, job satisfaction and engagement*</u>

Job satisfaction, professional confidence and motivation were more evident in teams with effective supervision. One CHW commented that since the PN arrived: *"we started to feel that we are now CHWs and human beings." (CHW-FGD, 1A)* Two ENs mentored by PNs, explained: *"At first it was challenging for me leaving the hospital and working in the community… maybe I'm not a nurse anymore, but now I feel more useful. The community is aware of us … they are coming to us. People are taking treatment now. I think there is lesser tracing on ARV patients, most defaulters you will find are TB. Our stats are going up" (EN, 1B) "I'm a street nurse…but you know what, when I reach the households, this is very enjoyable." (EN, 1A)* When CHWs were on a strike over issues with their contract in 2016, some of the CHWs at the clinic-based PN/EN site (1B) tried to carry on, not wanting to let their patients down or damage their relationship with supervisors and the facility. Their supervisor interpreted their actions: *"they see that you, as their leader, are supporting them and trying to understand where they come from, especially with this employer thing being mixed up. That is why I am saying, hence, they refused to go on strike today." (JN, 1B)*

For peer review only



For peer review only - http://bmjopen.bmj.com/site/about/guidelines.xhtml

However, in the absence of supportive supervision, insufficient training constrained CHWs ability to assist patients, and as a result, demotivated them. Their frustrations over their working conditions were unresolved, and not acknowledged by their immediate supervisors. As a result some CHWs decided to work fewer hours a day as a passive protest, in addition to the collectively organised strikes.

*Performance*

CHWs with engaged and skilled supervisors carried out a fuller range of activities, and used their discretion to make decisions, for example: *"A patient had an elevated blood glucose level. However, it was too late for the patient to go to the clinic, so rather than writing a referral letter, she visited the patient again with glucometer after work, by which time the glucose level had dropped by 50%. The next morning, she went for a third time and found that sugar level had returned to normal." (Observation notes, 1A*) This CHW, from a clinic-based PN/EN team, was able to interpret the glucometer readings, and re-checked the patient to avoid making an unnecessary referral.



For peer review only - http://bmjopen.bmj.com/site/about/guidelines.xhtml

The dedication of some CHWs to their work is evident in the following client interview: *"If you send a message to a CHW, it does not matter what time it is, or what the problem is, they will come and help you. Whatever problem you have, you can talk to them, and you are not worried that they will be talking about your problem around the area. I like the work they do very much." (Patient-BT, 2A)* In the EN-only model where supervision was the weakest and support from facility staff was negligible, CHW activities were minimal, with CHWs often only delivering medication to the elderly. One CHW highlighted how a colleague's poor performance impacted on the collective reputation of the team, *"It's not good if the patient comes here and when one of the clinic staff checks the card finds out the medication delivery is long overdue. It affects all of us in the eyes of the clinic staff and the PN." (CHW-FGD, 1A)*

<u>*Collaboration between CHWs and facility staff*</u>

Located at a distance from the main clinics, health post teams received little supervisory support from clinic staff. Other than supplying medication, there was little managerial oversight by the clinic. *"I am not directly supervising them except for sometimes when they are there. You can only supervise something that you can see. With the CHW team it is just via the phone and then it ends there."*



For peer review only - http://bmjopen.bmj.com/site/about/guidelines.xhtml

*(Facility Manager, 2A)* In the health posts located in communities without a clinic, the PN spent a considerable amount of time seeing patients, and so neglected her CHW supervision responsibilities. Furthermore, the distance between the health post and the clinic hampered efforts to ensure CHW's patients accessed to care at the clinic.

*"The saddest part is when the patient comes to the clinic and the file is not there, the doctor does not see the patient, and the patient is getting upset with us. Every time when we go to the clinic, we have to look for the files, the clerks do not assist us and we don't even find those files."(Observation notes from conversation with CHW, 2A)*

In clinic based team, without a senior supervisor to assist, management of files was also a problem:

*" Sometimes you find the admin clerk is in a bad mood, she speaks to us in a bad way in front of the patients, "I am not going to give you the file. You have to go back to the patient to tell the patient to come to the clinic to collect it." You report to the sister,*

For peer review only



For peer review only - http://bmjopen.bmj.com/site/about/guidelines.xhtml

*she will tell you that she has been warning her for several times but she does not listen." (CHW-FGD, 3A)*

CHWs reported that other facility staff expressed a dismissive attitude towards them:

*"the peer educators, HIV/AIDS counselor we all go together to sign the same contract but they are treated as if they are more educated than us, they call us street maids" (CHW-FGD, 3B)*

When asked why they were not allowed to work inside the facility, the CHWs responded: *"the manager tells us that we are not part of the clinic, so there's nothing she can do for us."* (field notes, 3A) The shortage of space exacerbated the CHWs' sense of marginalisation and exclusion.

However, in clinic based teams, with the support of senior supervisors, and the resulting growth in skills and confidence, CHWs had been able to establish better relationships with clinic staff. In turn, clinic staff, seeing the CHW's greater motivation and effort, were more likely to treat the CHWs with respect. There were several benefits to this resulting collaboration. Firstly, communication and



For peer review only - http://bmjopen.bmj.com/site/about/guidelines.xhtml

coordination was better. Working together, the facility manager and PN were able to confirm whether, once defaulters had been traced by CHWs, they returned to the clinic. *"We engage with facility manager, she helps with problems that we have... we include her in everything" (EN, 1B)*. Clinic staff willingly accepted referrals from CHWs, and included the CHWs in training sessions: *"Sister X is not (in the CHW team), but she is very helpful. She attended severe malnutrition training and she called all the CHWs and did a presentation for everybody*." *(EN, 1B).*

Secondly, in one clinic, the contribution of CHWs had become indispensable: *"If they (CHWs) were not there, it means you will be over worked, the clinic will always be full, to be honest. There is good communication between us." (Facility Manager, 1A)*

The third benefit was the empowerment of CHWs, who learned to negotiate with other less willing staff: *"I have a patient who doesn't want to come to the clinic and I don't want my patient to default on her HIV treatment. I said to the sister 'my patient is working at the farms so please pack medication for her. I'll ask her to come next month.' The sister scolded me because the patient did not come on her last appointment. So I begged the sister and promised to bring the patient myself.*



For peer review only - http://bmjopen.bmj.com/site/about/guidelines.xhtml

*Then it was sorted, the patient got her medication and she came to the clinic with me." (CHW-FGD, 1B)*

<u>*Negotiating acceptance and building the community's trust*</u>

All CHW teams depended on the supervisor to liaise with key community members:

*"We established good relations with the ward councillor so whenever there was a problem I would call her." (PN, 2B)* The presence of the senior supervisor increased CHWs' credibility in the community: *"If CHWs found that I am not well, they report to the PN, and the PN would come and see me. I have noticed that when I follow the PN's advice, my health improves. We are alive today because of them." (Patient-BT, 2A)*

Box 1 describes visits to two different households conducted on the same day by the same CHW, for which she received very different support from the facility.

**Box 1: Support from the clinic facilitates CHW performance and client trust**

<u>*Case 1 : Patient defaults as nurse insists on waiting for test results*</u>

The patient, a woman on ARV with a spinal cord problem, had moved recently. She was no longer able to collect ARVs and go for physio appointment at the clinic near her previous home as she had no money for transport. She asked for a referral to a closer clinic. The nurse



For peer review only - http://bmjopen.bmj.com/site/about/guidelines.xhtml

refused as ARV patients tended to move clinics, so patient stole the file.

The CHW took her file to the closer clinic to ask if she could collect medication for the patient. The CHW was told that a new file had to be opened and the patient had to wait for CD4 results to come back. As a result, when we interviewed her, the woman had been defaulting for the past 3 weeks.

In the interview the patient said the CHW had not followed up on her requests for food parcels as they had no food, for a physio appointment, or for her husband to be tested for HIV. *"The CHW doesn't follow up on me to see how I am doing; the only thing that she is good at is to make empty promises."* *(Patient-AA, 3A)*

*Case 2: Social worker responds to CHW request*

The patient was a woman whose child was knocked down and killed by a car in front of her and her children. The family were traumatised. CHW wrote a referral letter to a social worker. The patient went to the social worker and then started attending a support group. CHW also arranged for the social worker to see her husband and children at home.

Patient said she trusted CHW because *"she is kind, open and she knows how to speak to people even if you are in a difficult situation. She does not gossip about her patients on the street."* *(Patient-Z, 3A)*

Inadequate and unpredictable support from the clinic profoundly influenced the CHW's ability to provide care for her clients, and in turn, the degree of trust between client and CHW.

Table 4 summarises the key effects of location and the level of supervision on CHW performance.

**Table 4: Impact of supervision and location on the determinants of CHW performance**

| | Clinic-based with PN and EN | Health post with PN and EN | Clinic-based with EN only |
|---|---|---|---|



For peer review only - http://bmjopen.bmj.com/site/about/guidelines.xhtml

| | | | |
|---|---|---|---|
| **Mentorship of EN and CHW** | Good | Depending on how busy the PN was seeing patients | • Junior ENs were not able to provide adequate training or supervision<br>• Little as facility manager was not engaged; |
| **Relationship with clinic** | Good | Poor | Poor |
| **Files** | Well managed by PN | Files at clinic were not managed by PN & EN; often lost and CHWs had to go and find them | Part of clinic filing system |
| **Effect on quality of service** | • More able to ensure patient gets care needs<br>• Better relationship with community | • Limited service at health post; patient had to travel to clinic for anything other than basic service;<br>• Referrals were more difficult because of distance between health post from clinic; | • CHWs shied away from difficult cases, knowing that they did not have the support;<br>• Poorer relationship with community |

For peer review only



For peer review only - http://bmjopen.bmj.com/site/about/guidelines.xhtml

**DISCUSSION**

In this paper, we explored the effect of three different supervision and location configurations on CHW motivation and performance. Our findings show that experienced supervisors employed a range of strategies to train, motivate, monitor CHWs, to improve the quality of their work. In turn CHWs were treated with respect by the clinic staff, making space for collaboration and support that benefited both parties. Being an equal of the facility manager, a PN supervisor was in a position to bridge the divide between the CHWs and clinic staff. By obtaining a place for the CHWs inside the facility, as one of the supervisors did, she secured a position – physical and symbolic – for them in the health system. She drew in other staff to participate in the supervision of CHWs, further defending their legitimacy. Clinic staff provided advice and ensured the CHWs' patients accessed the care that they needed; CHWs traced the defaulting patients in the clinics and so improved the clinics patient statistics. The senior supervisor, a shared asset, was able to effectively integrate the CHWs into the health system.

In contrast, the teams in health posts found it difficult to build relationships with the clinic, particularly if the health post is at some distance from the clinic and the PN was busy with patient consultations. The EN-headed teams lacked adequate training



For peer review only - http://bmjopen.bmj.com/site/about/guidelines.xhtml

and supervision (partly due to their rural location), and consequently the CHWs' performance was minimal. With little evidence of the CHWs' contribution, the social distance between the CHWs and clinic staff, and her junior position, the EN was not able to facilitate a collaboration. Despite being based in the clinic, the CHWs were not effectively integrated into the health system.

Our study has several limitations. Firstly, despite fieldworkers asking CHWs to carry out their duties as normal, during observation many CHWs appeared to make extra effort to reach a greater number of households. We followed the same CHWs for 3-5 days to mitigate this, and the number of household visits did fall overtime, as the CHWs became used to the presence of an observer. Second, the information on performance is based on what we learned from our respondents rather than on an objective measure of performance. Third, while we selected the sites to ensure the different supervisory configurations were included, we do not know to what extent the included CHW teams are representative of other CHW teams in the District. The study demonstrates noteworthy strengths. As far as we know, this is the first study to examine the effect of different levels of on-site supervisors as well as the location on CHW performance in a government programme. The case study approach allowed us to purposively select teams based



For peer review only - http://bmjopen.bmj.com/site/about/guidelines.xhtml

on their different supervision/ location configurations, and to compare and contrast their functioning in order to understand what worked, and what did not.

CHW supervision is often no more than periodic interactions by facility staff or officials who may not understand the context and role of CHWs, resulting in bureaucratic inspection and fault-finding, rather than problem solving and support.[6, 7, 20] For example, in the Ethiopian national programme, supervisor make irregular visits from either the local health centre or district offices.[21] In our study the presence of an on-site supervisor enabled on-going supportive engagement, rather than a distant supervisor, who had fit supervisory visits into a busy schedule. Evidence from a rapid assessment of the South Africa WBOT programme in five provinces suggested the facility-based staff were unable to provide effective supervision as they were overstretched, working in under-resourced and under-performing clinics. [15] The authors concluded that CHW teams should be based in stand-alone health posts with a nurse supervisor, rather than a clinic. Our data suggests it would be more advantageous for the nurse supervisor and the CHWs to be based the clinic, with clear rules about how much time the supervisor can spend on clinic activities.



For peer review only - http://bmjopen.bmj.com/site/about/guidelines.xhtml

In the Ethiopian programme, CHWs, based in health posts, also struggled to make referrals and experienced dismissive attitudes of clinical staff; where there was more regular contact, the CHWs felt valued and part of the team.[21] Studies in Uganda, Bangladesh and Bolivia reported contact with a higher level of the health system gave CHWs credibility with their clients.[22-24] Similar to our study, in Mozambique supervision increased the participation and voice of CHWs in the intervention facilities.[13]

Training and supervision of the supervisors is a more neglected subject than that of CHWs.[9, 25, 26] This issue is beyond the scope of this paper, but in the second phase of our 3-year study, we are evaluating the effect on performance of a senior supervisor in building the capacity of the junior supervisors.

There are on-going global efforts to scale-up CHW programmes as a means to strengthen health systems and accelerate universal health coverage. These include one million CHWs in sub-Saharan Africa,[27] making CHWs available everywhere on the African continent, [28] developing guidelines by WHO, as well as the latest funding commitment from the private sector.[29] Our study suggests that it is worthwhile to make senior supervision an explicit component of CHW programmes.



For peer review only - http://bmjopen.bmj.com/site/about/guidelines.xhtml

It takes funds, understanding of context specificity, as well as innovation, to implement cost-effective supervisory mechanisms. This research has offered some recommendations based on the experience of well-functioning teams (See Table 5).

For peer review only



For peer review only - http://bmjopen.bmj.com/site/about/guidelines.xhtml

## Table 5: Programme design features and supervisory strategies that worked

| | Recommendation | Rationale |
|---|---|---|
| Programme design | • Attachment to PHC clinic | Facilitate physical & operational integration into the health system |
| | • Team up senior and junior supervisors | Build relations, guide CHWs to navigate through the community and system, pass down know-how to junior supervisors, |
| | • Setting guidelines as to how much time supervisors can spend assisting in the clinic | Help to acknowledge that the engagement is a two way collaboration, in which there are benefits for everybody |
| | • Strengthen HR management practices | Build trust, improve dialogue in the workplace, problem solving supervision, and culturally appropriate communication. |
| Supervisory strategies | • Supervise home visits | Provide opportunities to strengthen CHWs' knowledge and skills, demonstrate a strong backup for CHWs in the community, keep updated of community's status |
| | • Formal and on-the-job training | Impart knowledge and skills |
| | • Regular debriefing/feedback | Individual and collective supervision, track performance, build up teamwork spirit |
| | • Examine daily logs and registers | Ensure accurate documentation and subsequent reporting |
| | • Direct CHWs to tasks, such as tracing defaulting patients, that explicitly assist the clinic | Ensure the clinic staff are able to see the benefits of having CHWs as part of the team |
| | • Draw in clinic staff to work with and train CHWs | Improve the extent and quality of working relationships between clinic staff and CHWs, and allows the CHWs to benefit from the clinic staff's expertise. |
| | • Assist data collating & reporting | Ensure that CHW activities are accurately reported so that health system managers can see the benefits of the programme |
| | • Administration and logistics | Resolve administrative matters, negotiate for better work conditions, ensure CHWs are adequately equipped to deliver service |

For peer review only

## CONCLUSION

With high expectations of the CHW programme in improving access to care in South Africa and other similar settings, we found that full-time, on-site supervision can make an important contribution in enabling CHWs to provide comprehensive promotive and preventative care. In the context of an overstretched health system, senior supervision located within a clinic, can enable the development of a collaboration between the clinic and CHWs, reducing the CHW's marginalisation, and enabling their potential contribution to improving health outcomes to be realised.

For peer review only

### Contributorship

YHT interpreted data, wrote and revised the manuscript with JG, and approved the final manuscript as submitted.

FG and JG conceptualized and designed the study, analysed data, critically revised the manuscript and approved the final manuscript as submitted.

JKD carried out data collection process, managed data, analysed data, critically revised the manuscript, and approved the final manuscript as submitted.

NN analysed data, revised the manuscript and approved the final manuscript as submitted.

TR and HM analysed data and approved the final manuscript as submitted.

### Acknowledgements

The authors would like to acknowledge the following individuals for their invaluable contribution: the field workers, the respondents, the CHW teams, Mrs Salamina Hlahane, district manager at Sedibeng, and Mrs Bridget Lefhoedi, community-based services coordinator. Input from Prof. Margaret Thorogood,



For peer review only - http://bmjopen.bmj.com/site/about/guidelines.xhtml

David Sanders, and Emmanuelle Daviaud throughout the study has been highly appreciated. We also thank these organizations for their generous support: Department of Health Sedibeng District, Gauteng Department of Health, the UK Medical Research Council (MRC) and Taiwan Ministry of Science and Technology.

**Endnotes:**

Re-hired retirees were used for three reasons. Firstly due to the shortage of professional nurses in the system, drawing in those who had retired, increased the supply. Secondly, they had a wealth of experience in community nurses, which younger nurses often did not. Thirdly, they were given an increment on top of their pension, and so cost the district less than a formally employed professional nurse.

**Competing Interest**

None.

**Data sharing**

A sample of site-specific summary is available in appendix.

**Funding**

MRC-UK under Health Systems Research Initiative (MR/N015908/1)
Taiwan Ministry of Science and Technology (105-2917-I-564-006)



For peer review only - http://bmjopen.bmj.com/site/about/guidelines.xhtml

## REFERENCES


1. Perry HB, Zulliger R, Rogers MM. Community health workers in low-, middle-, and high-income countries: an overview of their history, recent evolution, and current effectiveness. *Annu Rev Public Health* 2014;35:399-421.
2. Datiko DG, Yassin MA, Theobald SJ, et al. Health extension workers improve tuberculosis case finding and treatment outcome in Ethiopia: a large-scale implementation study. *BMJ Glob Health* 2007;2**:**(4)e000390.
3. Lehmann U, Sanders D. Community health workers: what do we know about them. The state of the evidence on programmes, activities, costs and impact on health outcomes of using community health workers. Geneva: World Health Organization 2007:1–42.
4. van Ginneken N, Lewin S, Berridge V. The emergence of community health worker programmes in the late apartheid era in South Africa: An historical analysis. *Soc Sci Med* 2010;71:1110-8.
5. Barnett ML, Gonzalez A, Miranda J, Chavira DA, Lau AS. Mobilizing Community Health Workers to Address Mental Health Disparities for Underserved Populations: A Systematic Review. *Adm Policy Ment Health* 2017;1-17.
6. Hill Z, Dumbaugh M, Benton L, et al. Supervising community health workers in low-income countries–a review of impact and implementation issues. *Glob Health Action* 2014;7:24085.
7. Glenton C, Colvin CJ, Carlsen B, et al. Barriers and facilitators to the implementation of lay health worker programmes to improve access to maternal and child health: qualitative evidence synthesis. *Cochrane Database Syst Rev* 2013;10.
8. Kok MC, Kane SS, Tulloch O, et al. How does context influence performance of community health workers in low- and middle-income countries? Evidence from the literature. *Health Res Policy Syst* 2015;13:1-13.
9. Pallas SW, Minhas D, Pérez-Escamilla R, et al. Community health workers in low-and middle-income countries: what do we know about scaling up and sustainability? *Am J Public Health* 2013;103:e74-e82.
10. Kok MC, Dieleman M, Taegtmeyer M, et al. Which intervention design factors influence performance of community health workers in low-and middle-income countries? A systematic review. *Health Policy Plan* 2014;30:1207-27.




For peer review only - http://bmjopen.bmj.com/site/about/guidelines.xhtml

11. Bosch-Capblanch X, Liaqat S, Garner P. Managerial supervision to improve primary health care in low- and middle-income countries. Cochrane Database Syst Rev 2011;9.
12. Rabbani F, Shipton L, Aftab W, et al. Inspiring health worker motivation with supportive supervision: a survey of lady health supervisor motivating factors in rural Pakistan. *BMC Health Serv Res* 2016;16:397.
13. Madede T, Sidat M, McAuliffe E, et al. The impact of a supportive supervision intervention on health workers in Niassa, Mozambique: a cluster-controlled trial. *Hum Resour Health* 2017;15:58.
14. Ameha A, Karim AM, Erbo A, et al. Effectiveness of supportive supervision on the consistency of inter-grated comunity cases management skills of the health extension workers in 113 districts of Ethiopia. *Ethiop Med J* 2013;52:65-71.
15. Marcus TS, Hugo J, Jinabhai CC. Which primary care model? A qualitative analysis of ward-based outreach teams in South Africa. *Afr J Prim Health Care Fam Med* 2017;9:1-8.
16. Schneider H, Schaay N, et al. The challenges of reshaping disease specific and care oriented community based services towards comprehensive goals: a situation appraisal in the Western Cape Province, South Africa. *BMC Health Serv Res* 2015;15:436.
17. Department of Health, South Africa. Provincial guidelines for the implementation of the three streams of PHC Re-engineering. http://www.jphcf.co.za/wp-content/uploads/2014/06/GUIDELINES-FOR-THE-IMPLEMENTATION-OF-THE-THREE-STREAMS-OF-PHC-4-Sept-2.pdf (accessed 23 Jan 2017).
18. Trafford Z, Swartz A, Colvin CJ. “Contract to Volunteer”: South African Community Health Worker Mobilization for Better Labor Protection. *New Solut* 2018; 27:648-66.
19. Nxumalo N, Choonara S. *A rapid assessment of ward-based PHC outreach teams in Gauteng Sedibeng district–emfuleni sub-district.* Johannesburg: Centre for Health Policy, University of the Witwatersrand, 2014.
20. Roberton T, Applegate J, Lefevre AE, et al. Initial experiences and innovations in supervising community health workers for maternal, newborn, and child health in Morogoro region, Tanzania. *Hum Resour Health* 2015;13:19.
21. Kok MC, Kea AZ, Datiko DG, et al. A qualitative assessment of health extension workers’ relationships with the community and health sector in Ethiopia: opportunities for enhancing maternal health performance. *Hum Resour Health* 2015:13:80.



For peer review only - http://bmjopen.bmj.com/site/about/guidelines.xhtml

22. Bartos A, Tenorio A, Saunero R, et al., *Extending the duration of exclusive breastfeeding in El Alto, Bolivia through a community-based approach and the provision of health services.* Geneva, Switserland: Child Health and Nutrition Research Initiative, 2009.
23. Nankunda J, Tumwine JK, Soltvedt A, et al. Community based peer counsellors for support of exclusive breastfeeding: experiences from rural Uganda. *Int Breastfeed J* 2006;1:19.
24. Haider R, Ashworth A, Kabir I, et al. Effect of community-based peer counsellors on exclusive breastfeeding practices in Dhaka, Bangladesh: a randomised controlled trial. *Lancet* 2000;356:1643-7.
25. Daniels, K., et al., Supervision of community peer counsellors for infant feeding in South Africa: an exploratory qualitative study. *Hum Resour Health* 2010;8:6.
26. Rowe AK, Onikpo F, Lama M, Deming MS. The rise and fall of supervision in a project designed to strengthen supervision of Integrated Management of Childhood Illness in Benin. *Health Policy Plan* 2009;25:125-34.
27. Singh P, Sachs JD. 1 million community health workers in sub-Saharan Africa by 2015. *Lancet* 2013;382:363-5.
28. The Lancet Global Health. Community health workers: emerging from the shadows? Lancet Glob health 2017;5:e467.
29. Winters N, O'Donovan J, Geniets A. A new era for community health in countries of low and middle income? *Lancet Glob Health* 2018:6:e489-90.


For peer review only



For peer review only - http://bmjopen.bmj.com/site/about/guidelines.xhtml

Case site summary
Site: A Author(s): JG Date:22 02 2017

| | |
|---|---|
| **Model** | Clinic based team, although doesn't perform all standard clinic services (e.g. TB)<br>One PN, two ENs |
| **PN** – background/ experience, age, role, actual responsibilities | Professional nurse, midwifery, community health worker, at X for 6 years then Y hospital, retired at X, then went to the UK for X years .. started to work in Y at 62 .. now 72 years<br><br>**PNO:** once CHW have gone into the field, and have explained several things to the FW, spends an hour seeing patients.. and then leaves (so no meeting after CHW return)<br><br>**PNO_NM:** interview with FW, patient consultations for 2 hours; after 11.20 she reads patient files / notebooks; She also see family planning patients; (FW-PN doesn't seem to have a lot of work to do) |
| **EN**– background/ experience, age, role, actual responsibilities | **<u>EN01</u> - responsible for ward X**<br>**X** years old, became a EN in X, worked in private clinic before here, not from the area<br>FW comment: the EN seems passive.. maybe due to helplessness around CHW.. she struggles to maintain basic level of supervison by checking and doing CHW stats. Not clear how much time she spends in the clinic<br>*JG: from first meeting we know this EN doesn't like going out into the community .. it may be that she prefers to work in the clinic. When it comes to observations, she didn't actually do the stats because EN02 had given it to the data capturer to do because EN02 was going on training .. so sounds as if EN02 does most of the data reporting etc.*<br>**Observation day 1:**<br>Private meeting between PN and EN<br>Check on crèche that children have been immunized – no really confirmation of this<br>HH1: EN manages interaction with hh – follow up<br>Check on a pregnant woman at the crèche: registration done by CHW<br>HH2: patient there, but says that gate is locked;<br>HH3: patient not there<br>HH4: EN leads the interaction – follow up<br>FW: thinks EN went into the field because FW was there to observe .. but not real need for her to be there.<br>**Observation day2:**<br>Works in clinic as they are short staffed – so can't be observed; lunch and then knocks off as ½ day;<br>Next week she is on training so will ask receptionist to help with the stats. She will sign books to show she has checked them<br><br>**<u>EN02</u>** used to work in critical care unit in private hospital<br>Not sure if I am a nurse any more .. but I feel more useful<br>Much more active, tries to solve problems, has set up whats app group, able to provide supervision<br>**Observation day 1:**<br>Creche campaign; EN is leading activities<br>**Observation day 2:**<br>Works in clinic as short-staffed; sorting patients and then doing child immunisations;<br>Comes out to check on CHW to allocate them to FW<br>HH1 CHW leads<br>HH2 CHW checks patient, but EN worried so asks questions about stomach<br>HH3: EN leads<br>HH4: EN asks about granny; in hospital; EN is shocked because here only 2 weeks ago, checks whether she has enough medication etc *(suggests that EN02 does go regularly to the field)*<br>HH5: jointly doing activities<br>HH6: CHW leads, didn't ask about child<br><u>HH7</u>: Jointly leading<br>Return to clinic at about 12 noon<br>*FW comment: EN is very supportive of CHW*<br>**Observation day 3:**<br><u>HH1</u>: on way to support group visited a lady who had given birth to twins- just checked on babies<br><u>Support group</u>: EN sits at a distance, CHW know how to do their work; she only intervened when a BP was too high, and when difficult TB case arrives (HH interview: CHW bring sputum bottle, but patient fails to provide) ; not 12 noon so there is time to see a few households |

For peer review only

| | |
|---|---|
| | HH2: not allocated to a CHW, but she checks on him because he is sick with TB; EN leads (as she hasn't been before); EN says they will come and do registration even though not in demarked area<br>HH3: car accident victim and HIV<br>HH4: EN quiet, CHW leads<br>Back on counsellors place by 1pm after 30 mins walk<br>**JG:** *EN seems works hard, happy to go and find extra households, seems to know some households – evidence that goes into the field regularly*<br>**Observation day 4**<br>Discussion about working on Fridays they do stats – cHW said we don't; EN said that is only the last Friday of the month .. so today they are going into the field; Later EN says later .. eish we are working on a Friday .. this is slavery<br>HH1; came to give referral letter for NGO.. which is now closed for the year;<br>CHw says I will take you to the patient you didn't manage to see on Monday<br>HH2: patient discharged from hospital in pain; EN tried to help – offered to call an ambulance, but woman said no because nobody to go with her; suggested clinic on Monday – ok, daughter will take me; asked for daugther's number; can't get it out of the phone<br>HH3: checking on patient that they referred two days ago; she hadn't gone ; doesn't take her treatment;child without immunization; EN quite upset<br>HH3: healthy baby; EN happy ☺ |
| **CHWs** – average details across team (Number, age, gender, years' experience, education) | X CHWs in FGD, (X reported to part of team) , all female, (another X waiting to be registered. PN wants them back)<br>Average age 39 (range25-49)<br>64% of have some secondary education;<br>29% have matric<br>7 years on average as a CHW (range 3-16)<br>Average number of households=262 (248-317) (only Sicelo and Biopatong gave specific numbers per CHW)<br>25 mins walk from home to clinic (range 2-50)<br>18 mins walk from clinic to households (2-45)<br>93% have phase 1; 21% have phase 2 |
| Community profile (eventually draw in survey data) | **PN:** Ward X contains a large informal settlement **(FDG SM?)**<br>Ward 10 predominately a township **(FDG AS?)**<br><br>**PN:** We started going from house to house, counting people during the registration.. according to where the CHWstayed.. initial plan was 250 households, but didn't work .. either 190 or 200 (if medical aid we skip it)<br><br>People report to us if somebody is sick, they will even break down the door<br>**ENO2** (ward X) – area is huge - shortage of CHW<br>3 areas within ward X<br>Ext X and X informal settlements / shacks – 5 is too big<br>Ext X – RDP house<br>People are moving to Rdp houses, go back 3 days later and people have moved..<br>Lots of Zim patients, but language not a barrier<br>Informal settlement, lots of crime, didn't feel safe at beginning,, but not easy for CHW to be robbed because most of them are well known in community |
| Challenges? (Not general) | **FM:** Storage is a problem – for files,<br>Safety .. too many break ins in the clinic<br>CHW need more training, and then more CHW will hopefully come later<br>**FDG-SM**<br>Equipment – one CHW has take the BP machines that side .. so you call her to ask her to bring this side .. call from counsellor .. and we don't have machines<br>Behaviour of nurses – some patients refuse to come to the clinic because of the bad treatment. However we encourage them for the sake of their health to ignore the nurses attitude P11<br>Queuing – it irritates the patients .. so it gives us a problem when we try to encourage the patients to go to the clinic ..<br>Clocking in WBOT promised we would work around where we stay.. they promised a clock in Ward 8 .. we have found a place for it .. it is tiring to have to come to ward 10 to clock in .. go back to ward 8 and then come back to the clinic .. it is a distrance and it is hot or raining!<br>Stipend, job security, career development, smart purse<br>**FDG_AS**: as above plus job safety, training, recognition and appreciation<br>We can't even go to the bank with this card, only to shoprite .. otherwise they charge us |

| | |
|---|---|
| | **EN02 Obs:** currently leave is pause .. because smart purse says 13 days, contract says 21 days – hey have sent a letter to smart purse – EN really interested in solving problems .. but sometimes above her |
| Planning (of the day) | **PN:** I have paired them, an intelligent one with a less intelligent one |
| Weekly pattern of activity (what do they do when?)<br>- overtime | **FM:** one of their strengths is tracing .. they will find the person p18<br>CHW say that they do overtime<br>**FM:** says they don't<br>**FM:** may be ENs help out in the clinic 1 day out of 5<br>Did two campaign days during FW<br>**EN01:**<br>Run support group for elderly patients on Wednesday<br><br>**FDG-SM:**<br>We follow up on defaulters<br>Household registrations each day, - it is not possible to do registrations at one time of the year<br>Home-based care – we don't do much of this ..<br>we refer to social services<br>*JG: not convinced there is a day for this, and a day for that .. rather that they do all of the activities when needed.*<br><br>**FDG-AS:**<br>With Kgatelopele the first week I deliver medication, the second I will check if they are taking it properly by counting.. if it doesn't balance .. then I will ask her why .. then I will report to the clinic to the cinic that I am following up on this person . |
| Daily pattern of activity | 6 hh per day (for two CHW) (average is 5)<br>13 mins average per household (average is 12)<br>10 mins average travelling between households (average is 14)<br>5 hours work per day (average is 4.40)<br><br>5% of activities are meeting with supervisors (not time)<br>20% of activities are HIV as condition; (TB 5.5%)<br>52.75% of activities are other chronic<br>5.49% as mother and child<br>53% of recipients are elderly<br><br>Between 8-9 am do training<br>**EN02**: training and gym until 9am.. e.g. with new machines I take two CHW and show them so that they know what to do in the field<br>You can see a big difference between those who have attended the trainings..<br><br>On a Tuesday they are taking medication to patients, and from there to check on what ever<br>So if we have gone to 2 households, taking treatment, then I pick one house, any house that we haven't been to before ..and do a registration ..<br>Cos you find that their focus is on the patients that they know, the patients that they know<br>But with me I am always squeezing in just one house that has never been registered .. so I don't totally change their routine .. just one HH and you will find problems<br><br>**EN01:** 8am clock in and wait outside until 9am when go to households (no mention of training)<br>CHW work in pairs must see 6 households 3, 3, lunch break is between 12-1<br>They don't just to go their patients, they also visit other households after a longer break when no new patient is identified. People in the area also inform the CHW when there is somebody who is sick;<br><br>**FGD-SM:** 6 households per day each and households registrations is too much .. and we have to be back here by 13.30 .. our lunch is at 12 noon. We discuss issues we have with the nurse and pack medication to deliver (next day or on way home)?)<br>We write everything in our note book .. and then we note in our diary when we have to return to a household<br><br>**FDG-AS:** planning seems to be more about which of your houses and which of mine<br>• Last time they told us we had to see 6 patients a day per CHW .. now they have changed it to 4<br>• If you are doing household registrations .. you can't do 6 per day p13 |

For peer review only

| | |
|---|---|
| | • Sometimes we do home-based care, if the patient is living on her own and she is on treatment . .if ask if she has eaten .. if she has't I cook for her;<br>• We refer them .. the provider needs 8rand<br>• Sometimes we teach the family how to bath them |
| Record keeping & Stats – how & when<br>- How notes/info are kept<br>- How they are consolidated<br>- Patient files<br>- Follow ups | **FM:** Has improved since two ENs arrived<br>WE don't have email and our transport is irregular;<br>Now doing it once a week .. we used to do it once a month .. but then it was late<br>Two ENs are taking responsibility and they forward to the data capturer and then we come back and chat about it (Sister Tenqui and FM?)<br>**PN:** they have notebooks that they write in .. not more than 5/6 households per notebook .. (*so does that mean that they have 40 notebooks?)* I read those books every time I get a chance<br>**EN02:** she does monthly stats, she checks individual stats, talks to CHW saying why do you say this, or why haven't you write x, gives training to CHW.<br>In training we were told it would be easier if we did it weekly so I said to CHW lets do it on Fridays .. lets not go ot households but do stats instead .. now we are doing it monthly because we are faster now<br>From CHW to EN to facility manager<br>*JG: this is why they don't work on Fridays.*<br>**FDG-SM** we do stats daily .. at lunchtime we record evertying (some confusion about recording number of children)<br>**FDG-AS:** the OTL takes time to check them<br>I do them in my lunch hour, or at home, or when we are seeing my partner's patients |
| Referrals<br>- Forms/documentation<br>- Queueing<br>- Files | **FM** :Patient meant to show form at door and then goes to see Sister X, who then completes the form and gives back to the carer (if another nurses see patient they also complete the form)<br>**EN02:** Report number of referrals in stats as well<br>**FDG-SM**<br>"The referral form is causing trouble with the patients. The patients are supposed not to follow the queue, they should go straight to the PN but the patients are been shouted, told to go and follow the queue. Now the patients are fighting with us because we told them that with the referral they don't have to queue."p 15 |
| Medication delivery | **FM:** ENs can't come issue medication; so tengiwe does it . and if she is not here, they come to me with the vital signs data and the pill count, the condition of the patient, and the next clinic data. If the ENs are there the CHW will do this<br>**FDG-AS:** when you count their medication, sometimes they start shouting at you . you try to tell them that I am tyring to help you .. |
| Relationships Clinic – WBOT team | Other sisters will give medication out if necessary<br>CHW refer patients to specific sisters eg. For TB<br>**EN02** We do in-service training .. Sister X is doing EPS .. she is very helpful .. she is not WBOTS but she is very helpful - she is a staff nurse, but she has got more xperience … she attended severe malnutrition training .. she called all the CHW and patients .. shw as doing a presentation for everybody .. so it is not only us doing that .. She is also helping when we need something There is support from the clinic.<br>We engage with the FM for everything, problems we have, reporting .. she is part of the community, we include her in everything<br>District coordinator – I have only seen her once, when she came with those two ladies from wits !<br>Training person at X– if I have aproblem a message the OTL group, or even phone her .. she is giving support.<br>**FDG-SM**<br>"At the clinic we are treated as if we are not part of the clinic, they talk to us as if we are children. They shout at us in front of the patient that you brought for help. We don't have support at all. Another thing, they don't want us to eat breakfast but they eat when they come to the clinic. We consult TB patients on empty stomach. I did raise that to the PN and she said we should eat at home. Then I asked why she is not also eating at home in the morning and see if she can do it. In the morning we prepare our children to go to school, others have husbands to take care of before we leave at 7:00am, and we have to walk. We don't have time to have breakfast at our houses in the morning. We only have breakfast and lunch together at 12:00pm. There is no support at all." P15<br>**FDG-AS:**<br>"the sister in charge and our OTLs are treating us like.. I don't know .. as you can see we have no place to stay, no chairs, no table, nothing .. we came with the idea of starting the day with a prayer like in hospitasl.. but the sister in charge says we won't do such a thing here .. so we stopped because we are powerless' p14<br><br>**Any conflict with OTL or clinic staff** |

For peer review only

| | |
|---|---|
| | "We are all going to talk neh .. add to what I say .. we have our sister in charge, but we have another one .. the CHWs know who I am talking abut .. this sisteris controlling us more than our OTL" .. P21<br>"she even shouts at us infront of the patients. We are adults, mothers and we have our own houses and when we are here she treats us like children. She does not respect us." P21<br>"And sometimes we walk in, she shouts and says 'hey you is that a uniform, that is not the way we dress here.' Where are we going to get the new navy pant? That pant costs R190-R200.00. All that shouting she does it in front of everyone at the clinic. That is not okay and when you talk back to her, she will say that she is not our OTL and does report to us."<br>**Participant**: "We want her to be out of the WBOT team. We want to report to our OTL only. She is responsible for other nurses not us."<br><br>"My challenge as a CHW is that when I do home visit, some households do not have food to eat and the patient is on treatment. So I have to empathise with the patient, contribute something so that the patient can eat. When I come to the clinic and ask for this porridge, the clinic staffs tells me that I have to come with the patient so that they can see the patient. That is a big problem because the patient is sick and has no transport money to go to the clinic. So yah it hurts." P20 |
| Relationship EN & PN | **EN02:** if I need clarity I will phone here |
| Supervision of CHWs (including training) | **PN:** I read their books whenever I can; if they have difficult cases they bring them straight to me .. and I take the car and drive to see the issue ..<br>**EN01:** Most of the CHW are older than her.. they don't always listen to the EN. When they only have one household to visit they have all sorts of stories to get away. She reported this to the FM who said she would sort it out.<br>Two CHW communicate on behalf of the rest ..<br>They report to the EN when they have found a defaulter, they then revisit and then report<br>Weekly or bi-weekly she checks the activities of the CHW<br>FM told CHW not to come to work during strike<br><br>**EN02:** supervises 8 CHW, 5 have not been registered so they are using the book. If she knows CHW have much work to do, she leaves with them at 8 rather than 9<br>Different types of CHW – counsellor, peer educators, rest are CHW; one CHW is the leader whose job it is to forward complaints to her<br>If I have been on training I will ask how did it go with that patient, we have an whatspp group so we can communicate that weay .. if I am not around, they will send me a whatsapp and I will say do this or that.<br>I spend the whole day with them, .. and then I give them a lift home<br><br>Since the strike they have not been the same .. some they understand, some they are aggressive … so I said if you need something, or clarity, sit as CHW, write a memo /list and let your head CHW delivery it .. according to the hierarchy – this really helps, otherwise we have too many complaining and nothing will be sorted out<br>e.g. bags – why only 10 bags; so they wrote it down, I took it to my PN, and the facility manager who was able to sort it out; we discussed how they are going to use the equipment<br>*JG – example of sorting things out*<br><br>It goes back to the in-service training … when I am compiling the stats I am able to see this one is struggling .. when I do supervision I can see this one didn't do it right … I keep quiet in the house,, but immediately we step outside .. as we are walking I do spot training<br><br>Both FM and EN01 had stories of truancy (going home and coming back to clock out);<br>EN02 uses her car to drive them back to the facility if they need to clock out, or allows them to sign out tomorrow if they are working late<br>**EN 02** Sometimes they come back early because of the distance.. because it can be an hours walk … you can waste 2 hours a day walking backwards and forwards<br>**FDG-SM** do you work overtime? If we do household registration, and we don't find anybody who can has the information, we go back in the afternoon or the weekend .. .. Sometimes we deliver medication in the afternoon.. sometimes we are do vital signs at 7.45 |

| | |
|---|---|
| | **FDG-AS:** if some people are very sick we monitor them over the weekend<br>Our team leader is supportive .. if the patient wants to know something .. I ask her |
| Supervision of ENs | **FM:** do induction and appraisals of EN; provide training – HIV (going on Monday), TB (one has gone the other will go in 2017), EVP<br>**EN01:** if sister X is not around she goes to the FM and she provides a solution<br>They help out between 7.30 and 12.00 at the clinic when the clinic is full. When the clinic needs them they pick two CHW to stay and help the EN .. but they are free to say no<br>**EN02:** The facility manager makes sure we attend regular training to uplift ourselves She organizes workshops with CHW to train them on what she has learnt<br>Now I can lead campaigns – cos I understand everything about the campaign – so you see there really is development here..<br>My job is with WBOTs. 70% in the field, 30% in WBOT admin .. if I am not busy I help with vital signs stations.. it is not part of my job but I do it anyway .. I refer my patients and the nurses see them .. So there is teamwork .. The support system is great so I never felt pressure to leave my wBOT duties<br>**FDG-SM**<br>"And sometimes when you go with the OTL, the patients are feeling uncomfortable because she shouts at them. And we are unable to tell her that what you are doing is wrong, you are not supposed to talk like that with patients. Now the patients are telling us that we are not welcomed in their houses if we come with the OTL."<br>*JG- not clear which OTL is this. .. although know it is ward X* |
| Level of knowledge of CHWs | **FM:** Depends on education of the individual - some will do as you ask, others you need to dig deeper ..<br>**PN**: they are done some of the training (phase 1 and 2)<br>Are they able to implement it ? some are .. others are dragging, but they are trying p7<br>**FDG-SM – what cases aren't you able to deal with?**<br>Sick patient with no food >refer<br>Elderly patients who needs to be in old age home |
| Travel (time, mode, particular features/challenges) | |
| Resources Available | |
| Resources Needed | Have to buy batteries, transport, airtime (although do call-backs), forms, uniform out of own money (although towards the end, the CHW were wearing pink shirts)<br>Don't have office space, or masks for working with TB patients<br>**PN:** forms .. yes, one day a teacher was here from the primary school.. and I asked him to make copies<br>*No photocopier*<br>They need a permanent structure so that they can do maternity<br>We are using our own transport - that is what is irritating<br>**EN02:** Shortage of bags<br>**EN02:** I ma suing my own petrol, my own cellphone, chw use their own airtime.. sometimes they even call patients.<br>We need a place to meet as CHW /OTLS; patient files are stored in PN's room<br>We need our own social worker<br>**FDG_SM – description of the bag**<br>We are meant to screen everybody over 25 years for diabetes.. but we don't because the strips are not enough<br>The machine is already broken .. it is not strong<br>We don't have an office, airtime, batteries (we have to buy out of our money)<br>**FDG-SA:** we were given 10 bags for 17 of us; my machine isn't working.. I reported it .. it is embarrassing when it doesn't work; the strips are finished; we report to the sister who says that the patient must come to the clinic<br>You use your own airtime to call an ambulance;<br>If you are tracing, if you don't find that person at the address, you call them using your own airtime .. otherwise how are going to find them them<br>What makes us not to achieve more is that when we come to the clinic and ask for some more equipment, we are told that that they don't have' |
| Community relationships | **FM:** Initially not accepted, were chased away .. now they are accepted. ..have built rapport<br>community unhappy when CHW on strike (they were saying nurses were on strike p6 |

For peer review only

| | |
|---|---|
| | can do dressings which patients appreciate<br>**PN**<br>• The relationship between the community and the CHW .. 'one day we were out walking with Dr Y and Dr X.. and somebody asked the CHW how come you are walking with so many men' .. she answered they are doctors we are working together' Dr X asked theperson .. how do you know my nurse? .. she brings me medication every month P6 .. Dr X was very excited that they were well known in the community p6<br>• That is why we allocate houses to them in their area.. because then people can call them over the weekend . and then they give us a report and say .. a patient was giving birth .. and so we called an ambulance etc .. it is better if the CHW is in the area<br>• We don't have a clinic committee .. but we have a very good relationship with the ward councilor (ward X?) (EN02 as well – if there is somebody who is sick he calls me, so we work toghether)<br>• I want to start a support group for young people with HIV<br>**EN02**<br>The community is aware of us .. they are coming to us. Even the referral forms its good<br>People are taking treatment now. I think there is lesser tracing on ARV patients, most defaulters you will find are TB. Our stats are going up<br>**Reason for success**<br>Employing us as staff nurses .. cos if the CHW see you as supporting them, trying to understand where they are coming from, especially with this employer thing is mixed up … That is why they refused to go on strike today<br>**EN02**<br>When they were on strike for 3 months .. the patients were complaining .. where have you been, we have been waiting for you .. they decided not to go to the clinic but wait until the sisters were back .. so they really rely on them<br>**FGD-SM**<br>We get the list of defaulters .. but now there are few because of our work .. people have now accepted their illness .. people do appreciate us.. just come and disclose to us .. and later that person will come and thank you for the work you have done p5<br><br>Iyhoo, I'm so proud about my patients, they get better. Others I found them bedridden but because of us they have recovered. Even if they don't give me any material thing, but when I pass them on the street and she/he tells others that she/he is walking because of me, I feel satisfied because at least I have made other people better." P15<br><br>"In ward 8 we don't have any conflict with the community because the ward councilor had introduced us during the public meeting. They know us and they trust us in a way that when they need help even if it is during the night, they wake us. People got that belief that when the CHWs call the ambulance, the ambulance arrives quickly. So we are don't mind because we know the community rely on us."<br><br>We already said, they are able to come to us. Like no the nurse at the clinic is unapproachable, she talks to the patients the way she likes and the patient keep quiet but when they see us, they tell us what happened with the nurse at the clinic. So it is easy for them to talk to us than to the nurses. Other patients said if the nurse comes to their houses and speaks the way she speaks when she is at the clinic, we are going to hit her. Then we try to calm them down." P15<br><br>If my neighbour is sick I ask my partner to attend to her.. cos I don't want the neighbour to say that I talk about her ..<br><br>"The community things we are their helper, they love us. In everything that we do, they stand with us." P25<br><br>"To honest the patients trust us and they appreciate us. I have a patient who is HIV and she doesn't want to come to the clinic and I don't want my patient to default. So I promised her that I will assist. Then I came to the clinic and talk to the sister. I said sister my patient is working at the farms so please pack medication for her, here is her clinic card. I'll ask her to come next month. The sister scolded me because the patient did not come to the clinic on her last appointment. So I begged the sister and I promised the sister that I will bring the patient myself. Then it was sorted, the patient came to the clinic with me and she got her medication." P18 |
| Relationships with other NGOs, | **EN02**<br>We work with policing forum, ward counsellor, we have their numbers .. we do campaigns together .. community and health campaigns (e.g.creches) |

For peer review only

| | |
|---|---|
| networking, knowledge of context (draw on what exists & links clients) | Household registration for municipality .. although you ask these questions about income.. they think you are bringing food .. so they make you feel like you are making empty promises)<br>NGO A – I have referred too many people there .. but there results are not good.. if people don't have an ID they come back with nothing. We need our own social worker |
| Overall impression<br>- Incl do CHWs feel valued?<br>- Other notes | During the strike some of the CHW tried to work .. but the EN and the FM told them not to<br>**EN02:** strike started again today but CHW refuse to be part of strike (strike is for better employment issues, high bank card deductions (20R), lack of communication from smart purse, uniform); eventually they had to accepted it .. so not joining strike today<br><br>**Can the programme be led by staff nurses ?**<br>**EN02** I am saying this because with staff nurses in the community … I can deal with almost 90% of the problems .. but as a staff nurse I am not allowed to give out treatment.. if I pre-pack medication .. it has to be checked by the professional nurse .. also I can't consult |
| Interesting points | • FM is involved, and knows how the WBOT programme runs, clinic was set up and the same time as the WBOT<br>• EN02 know there is a reciprocal relationship between WBOT and clinic and need to maintain that ..(helps when clinic is busy and they see her patients)<br>• If clinic is not welcoming to patients .. difficulty in persuading patients to go to the clinic .. So success of WBOT is dependent on quality of care in clinics<br>• In EN only model – a PN has to be available to dispense the medication (or check it?)<br>• It seems that EN02 who goes out into the community has a better relationship with the CHW, the trust each other more .. a lot of the problems are about lack of trust<br>• EN 01 is a lot more passive and prefers to work in the clinic;<br>• PN sees patients but doesn't seem to meet with CHW when they return .. EN02 knows households and really works with CHW |

For peer review only

Consolidated criteria for reporting qualitative studies (COREQ): 32-item checklist

| Item | | Guide questions/description | |
|---|---|---|---|
| **Domain 1: Research team and reflexivity** | | | |
| **Personal Characteristics** | | | |
| 1. | Interviewer/facilitator | Which author/s conducted the interview or focus group? | **Field workers trained by researchers collected data** |
| 2. | Credentials | What were the researcher's credentials? *E.g. PhD, MD* | **PhD, MD, MS and MA** |
| 3. | Occupation | What was their occupation at the time of the study? | **Professors, researchers, postdocs, and PhD fellows** |
| 4. | Gender | Was the researcher male or female? | **6 females + 1male** |
| 5. | Experience and training | What experience or training did the researcher have? | **Health policy, qualitative, health systems research, social work** |
| Relationship with participants | | | |
| 6. | Relationship established | Was a relationship established prior to study commencement? | **No** |
| 7. | Participant knowledge of the interviewer | What did the participants know about the researcher? e.g. *personal goals, reasons for doing the research* | **Researchers and fieldworkers explained to participants the reasons for doing the research** |
| 8. | Interviewer characteristics | What characteristics were reported about the interviewer/facilitator? e.g. *Bias, assumptions, reasons and interests in the research topic* | **Researchers explained the aims and objectives of the study to the participants.** |
| **Domain 2: study design** | | | |
| Theoretical framework | | | |
| 9. | Methodological orientation and Theory | What methodological orientation was stated to underpin the study? *e.g. grounded theory, discourse analysis, ethnography, phenomenology, content analysis* | **Case study** |
| Participant selection | | | |
| 10. | Sampling | How were participants selected? *e.g. purposive, convenience, consecutive, snowball* | **Purposive** |
| 11. | Method of approach | How were participants approached? e.g. *face-to-face, telephone, mail, email* | **Either telephonically, or face-to-face.** |
| 12. | Sample size | How many participants were in the study? | **201 individuals were being observed or/and interviewed.** |
| 13. | Non-participation | How many people refused to participate or dropped out? Reasons? | **Only one senior nurse supervisor. However, she didn't really refuse, but only rescheduled a number of times and we eventually took it as a reluctance to participate.** |
| Setting | | | |
| 14. | Setting of data collection | Where was the data collected? e.g. *home, clinic, workplace* | **Home for household members; workplace (incl. community and facility) for CHWs and supervisors** |

For peer review only

| | | | |
|---|---|---|---|
| 15. | Presence of non-participants | Was anyone else present besides the participants and researchers? | **Yes, during observations, where there were community members and clinic staff.**<br>**No, during focus group & interview.** |
| 16. | Description of sample | What are the important characteristics of the sample? *e.g. demographic data, date* | **Characteristics of the sample related to research are provided in Results, on pages 15-16, lines 204-224.** |
| Data collection | | | |
| 17. | Interview guide | Were questions, prompts, guides provided by the authors? Was it pilot tested? | **Yes.**<br>**Pilot tested between researchers and fieldworkers (facilitators).** |
| 18. | Repeat interviews | Were repeat interviews carried out? If yes, how many? | **Follow-up focus group discussions at 4 of the clinic-based CHWs** |
| 19. | Audio/visual recording | Did the research use audio or visual recording to collect the data? | **Interviews and focus discussions were audio recorded for transcription, as they were done in local language.** |
| 20. | Field notes | Were field notes made during and/or after the interview or focus group? | **Yes, notes were made for every piece of data, both during data collection and afterwards.** |
| 21. | Duration | What was the duration of the interviews or focus group? | **5 months** |
| 22. | Data saturation | Was data saturation discussed? | **Yes** |
| 23. | Transcripts returned | Were transcripts returned to participants for comment and/or correction? | **No** |
| **Domain 3: analysis and findings** | | | |
| Data analysis | | | |
| 24. | Number of data coders | How many data coders coded the data? | **All 7 authors** |
| 25. | Description of the coding tree | Did authors provide a description of the coding tree? | **No** |
| 26. | Derivation of themes | Were themes identified in advance or derived from the data? | **Both** |
| 27. | Software | What software, if applicable, was used to manage the data? | **Done manually** |
| 28. | Participant checking | Did participants provide feedback on the findings? | **Feedback has been given to provincial, district and facility management, as well as CHW supervisors and CHWs in the intervention sites in various forms, including facility level workshops. Participants generally agreed with the findings.** |
| Reporting | | | |
| 29. | Quotations presented | Were participant quotations presented to illustrate the themes / findings? Was each quotation identified? e.g. *participant number* | **Yes** |
| 30. | Data and | Was there consistency between the data | **Yes** |

For peer review only

| | | | |
|---|---|---|---|
| | findings consistent | presented and the findings? | |
| 31. | Clarity of major themes | Were major themes clearly presented in the findings? | **Yes** |
| 32. | Clarity of minor themes | Is there a description of diverse cases or discussion of minor themes? | **Yes** |

For peer review only

BMJ Open

# Integrating community health workers into the formal health system to improve performance: a qualitative study on the role of on-site supervision in the South African programme



| | |
|---|---|
| Journal: | *BMJ Open* |
| Manuscript ID | bmjopen-2018-022186.R2 |
| Article Type: | Research |
| Date Submitted by the Author: | 03-Oct-2018 |



| | |
|---|---|
| Complete List of Authors: | Tseng, Yu-hwei; University of the Witwatersrand School of Public Health, Centre for Health Policy<br>Griffiths, Frances; University of Warwick Warwick Medical School<br>de Kadt, Julia; University of the Witwatersrand School of Public Health, Centre for Health Policy Johannesburg, Gauteng, ZA<br>Nxumalo, Nonhlanhla; University of the Witwatersrand School of Public Health, Centre for Health Policy<br>Rwafa, Teurai; University of the Witwatersrand School of Public Health, Centre for Health Policy<br>Malatji, Hlologelo; University of the Witwatersrand School of Public Health, Centre for Health Policy<br>Goudge, Jane; University of the Witwatersrand School of Public Health, Centre for Health Policy |



| | |
|---|---|
| <b>Primary Subject Heading</b>: | Health services research |
| Secondary Subject Heading: | Qualitative research, Public health, Health policy |
| Keywords: | Human resource management < HEALTH SERVICES ADMINISTRATION & MANAGEMENT, Organisation of health services < HEALTH SERVICES ADMINISTRATION & MANAGEMENT, QUALITATIVE RESEARCH, PUBLIC HEALTH, Health policy < HEALTH SERVICES ADMINISTRATION & MANAGEMENT |
| | |


SCHOLARONE™
Manuscripts

# Integrating community health workers into the formal health system to improve performance: a qualitative study on the role of on-site supervision in the South African programme

Yu-hwei Tseng,[1] Frances E Griffiths,[1,2] Julia De Kadt,[1] Nonhlanhla Nxumalo,[1] Teurai Rwafa,[1] Hlologelo Malatji,[1] Jane Goudge[1],

**Affiliations**

[1] Centre for Health Policy, School of Public Health, University of the Witwatersrand, Johannesburg, South Africa;

[2]Division of Health Sciences, Warwick Medical School, University of Warwick, Coventry, United Kingdom

**Address correspondence to:**

Jane Goudge

Centre for Health Policy (CHP)

School of Public Health, Faculty of Health Sciences, University of the Witwatersrand

Private Bag X3 Wits 2050, Johannesburg, South Africa

Tel: +27 11 717 3425; Email: jane.goudge@gmail.com

**Keywords:** community health worker, supervision, South Africa, health system integration, health post

**Abstract word count: 300**

**Text word count: 7,152**

**Table: 5**

**Box: 1**

**Number of references: 29**



For peer review only - http://bmjopen.bmj.com/site/about/guidelines.xhtml

## ABSTRACT

### INTRODUCTION

Community health worker (CHW) programmes have the potential to improve access to primary health care and so assist in achieving universal health coverage. However, their effectiveness is often limited by inadequate supervision, and insufficient integration into the health system. South Africa is implementing a national CHW programme with on-site supervision. In this paper, we compare the functioning of CHW teams reporting to a clinic-based nurse with community-based teams supervised by nurses who don't have additional clinic duties. We also consider whether a junior nurse can provide adequate supervision, given the shortage of senior nurses.

### METHODS

In the initial observation phase of a 3-year intervention study, we employed multiple qualitative methods to study six CHW teams with different configurations of supervision and location: either a junior nurse, or a junior with a senior nurse, and either facility- or community-based. We observed activities of CHWs and their supervisors, conducted focus group discussions, key informant interviews, and interviews with clients.

### RESULTS

Effective supervisors supported CHWs through household visits, on-the-job training, debriefing, reviewing CHWs' daily logs, and assistance with compiling reports. CHWs led by senior nurses were motivated and performed a greater range of tasks; junior nurses in these teams could better fulfil their role. Clinic-based teams with senior supervisors were better integrated and more able to ensure continuity of care. In contrast, teams with only junior supervisors, or based in the community, had less engagement with clinic staff, and were less able to ensure necessary care for patients, resulting in lower levels of trust from clients.

### CONCLUSION

Senior supervisors raised CHW skills, and successfully negotiated a place for CHWs in the health system. Collaboration with clinic staff reduced CHWs' marginalisation, and increased motivation. Despite being clinic-based, teams without senior supervisors had lower skill levels and were less integrated into the health system.




For peer review only - http://bmjopen.bmj.com/site/about/guidelines.xhtml

For peer review only



For peer review only - http://bmjopen.bmj.com/site/about/guidelines.xhtml

**Strengths and limitations of this study**

- To the best of our knowledge, this is the first study to examine the role of different levels of supervision and location of the CHW team on the team's motivation, performance and integration into the health system.
- The case study approach allowed us to purposively select teams based on their supervision/ location configurations, and to compare and contrast their functioning to understand what worked, and what did not.
- We employed multiple qualitative methods, including participant observation, interview and focus group discussion, to collect a substantial amount of data, enabling triangulation across data sources and sites.
- Limitations to the study include the observer effect (Hawthorne effect) during data collection, subjective rather than objective evaluations of CHW performance, and data collection limited to a single health district.

For peer review only



For peer review only - http://bmjopen.bmj.com/site/about/guidelines.xhtml

**INTRODUCTION**

Community health workers (CHWs) play an important role in improving health care coverage and access in low and middle-income countries (LMICs).[1-3] However, optimizing the contribution of CHWs continues to be a global challenge. CHWs, with no professional qualification, perform primary health care (PHC) related functions in the community.[1, 4] They work on the periphery of the health system and often in under-served communities where needs are immense and multifaceted. For this reason, CHWs require frequent and supportive supervision that supplements their training and connects them with the health system.[3, 5, 6] Recent reviews suggest that adequate supervision, and integration into the health system, are essential components to programme effectiveness.[5, 7-9]

Effective integration of CHW programmes into the health system requires government financing, national level planning, training and CHW scope of work, supervision, referral networks, and supply chains to be connected to and incorporated into similar processes provided for other cadres.[9] Without integration, there is unlikely to be a smooth patient pathway from CHW to clinic, limiting the effectiveness of CHWs. There is a growing literature on supervision, with some evidence that supportive supervision improves motivation and



For peer review only - http://bmjopen.bmj.com/site/about/guidelines.xhtml

performance.[10-14] However, much of this evidence is from NGO programmes (which are less integrated into the health system), or from programmes in which the CHWs' work is focused on a specific disease (e.g. HIV) or population (e.g. maternal). With the goal of universal access to comprehensive, integrated care, it is important to understand the type and level of supervision that will enable CHWs to provide the full range of required tasks, as well as facilitate integration into the health system.[12-15]

Given the double burden of non-communicable and infectious diseases in LMICs, governments such as South Africa's are seeking to shift CHW initiatives from focusing on a single disease or population group, to a more comprehensive approach.[16] South Africa is implementing a national CHW programme (WBOTs; ward-based outreach teams) as part of efforts to strengthen primary health care.[17] The intention is to provide full-time on-site supervisors whose responsibility is to manage, train, mentor, and monitor CHWs and to facilitate links with the health system and the community. However, given the shortage of health care workers, as in other LMIC settings, there is a limited number of nurses available to supervise CHWs. In this study, we ask whether CHW teams should be linked to a clinic, and report to a clinic-based nurse who may facilitate referrals, access to supplies, and



For peer review only - http://bmjopen.bmj.com/site/about/guidelines.xhtml

CHWs' integration into the health system. Or should CHWs be based in the community in which they are working, with a dedicated nurse who doesn't have the additional demands of clinic duties? We also ask whether a junior (enrolled) nurse is able to provide adequate supervision, given the shortage of senior (professional) nurses. By asking these questions, we examine supervision practices, the team's integration into the health system, and their effect on CHW motivation and performance.

## METHODS

In the initial observation phase (Sept 2016- Feb 2017) of a 3-year intervention study in Sedibeng Health District, Gauteng Province we used a case study approach to examine the operation of CHW teams with different configurations of supervisors and locations: (1) clinic-based teams supervised by a senior nurse (professional nurse; PN) and a junior nurse (enrolled nurse; EN); (2) community (health post)-based teams supervised by a PN and an EN; and, (3) clinic-based teams supervised by an EN only. We studied six teams (two of each of the three configurations); each team and their supervisors being a single case study.

### Study site



For peer review only - http://bmjopen.bmj.com/site/about/guidelines.xhtml

In some South African provinces, CHWs, previously employed by non-governmental organizations (NGOs), have been absorbed into a government WBOT programme in an attempt to shift from a patchwork of condition-specific services to a comprehensive programme with a national scope.[15, 18] The CHWs' role is predominately health promotion, prevention, and screening. Standardized CHW training covers identification of the need for ante-natal and post-natal care, monitoring immunisation of under 5s, adherence among patients with chronic diseases, screening for malnutrition, TB, gender based violence, and making referrals to health, social and other services. WBOT teams are meant to be comprised of a professional nurse, six or more CHWs, one health promoter and one environmental officer,[17] although realities on the ground often do not allow this.

We selected Sedibeng Health District as our study site due to the variation in supervision structures and location of its CHW teams. The district is relatively affluent by South African standards, although over 20% of its residents fall below the food poverty line. Outside the urban areas, disadvantaged communities with inadequate shelter, food insecurity and high disease burdens have limited access to services such as clinics, transport, water and electricity.



For peer review only - http://bmjopen.bmj.com/site/about/guidelines.xhtml

Sedibeng District has established 16 health posts to provide community-orientated care, different from the disease focused care provided by 28 primary health care clinics.[19] A health post consists of one or two temporary structures (providing 3-6 rooms), often operating without electricity and with irregular water supply. It is managed by one or two PNs, who obtain medication and other resources via a 'mother' clinic. The nurses supervise the CHW team, and provide basic services such as chronic medication, immunisation and treating minor ailments. Some health posts are placed at some distance from a clinic, in order to improve access to basic services.

At the time of the study there were 39 CHW teams (each with between 6-20 CHWs) in 37 of the district's 72 wards (the smallest geopolitical area). Sixteen of the teams were based at health posts and the remaining 23 were clinic-based. In addition to the services outlined in the national training programme (see above), the CHWs delivered medication to elderly or disabled chronically ill patients.

**Selection of CHW teams for study**

The research team consulted with district officials, categorizing the CHW teams into three types (See Table 1). We selected two teams of each type with the requirement



For peer review only - http://bmjopen.bmj.com/site/about/guidelines.xhtml

that each pair of teams needed to be as similar as possible (important characteristics included urban or rural location, the size of the teams, and the type of community they served). (We needed to pool the data from the two teams to generate a sufficient sample for the analysis of the coverage data, reported elsewhere). Tables 1 and 3 describe the characteristics of different teams and their catchment areas. The two health post teams were closest to the community, while the rural teams had the largest geographical area to cover (Table 1).

**Table 1. Supervision configurations of CHW teams and their catchment areas**

| Type | Supervisor | Based in | Site Code | Description of urban/rural location and type of community served | Max. distance CHW base to furthest household |
|---|---|---|---|---|---|
| 1 | Professional & Enrolled nurse | Clinic | 1A | Township[1] with relatively well-off households and apartheid-era housing | 2 km |
| | | | 1B | Township with RDP[2] housing and informal settlement[3] | 3 km |
| 2 | Professional & Enrolled nurse | Health post | 2A | Township | 1 km |
| | | | 2B | Large informal settlement, with many migrants | 1 km |
| 3 | Enrolled nurse only | Clinic | 3A | Rural, informal settlement, also with RDP housing and farm plots | 6 km |
| | | | 3B | Rural, township and farm plots | 25 km |

[1] Township: densely populated urban area built on the peripheries of towns and cities; [2] RDP housing: low-cost government built housing; [3] informal settlements: where impoverished population build shacks on vacant land.

## Data collection

Eight fieldworkers received two weeks of training, covering research methods and ethics, study tools, extensive role-play and observation practice, as well as an overview of training that CHWs receive. Between Sept 2016 and Feb 2017, the field



For peer review only - http://bmjopen.bmj.com/site/about/guidelines.xhtml

For peer review only

team collected interview and observation data on each team's activities, resources, engagement with the clinic and community, successes and challenges, as well as client and key informant views of the performance of the teams. (Further details of CHW performance using more objective indicators, such as coverage and the quality of care, will be reported elsewhere.) Data collection method and types of data are summarised in Table 2

**Table 2: Data collection method, participants and data collected**

| Method | Participants | Total number in 6 sites | Data collected |
|---|---|---|---|
| Observation | CHWs and supervisors while conducting their daily work | 126 days of observation | Descriptions of activities, interactions and clients; |
| FGD[1] | CHW teams | 12 FGDs (76 participants) | FGD: descriptions of activities and weekly & daily routines; resources available and needed; support from supervisors, peers, clinic and community; employment conditions; challenges of the programme<br><br>Self-administered questionnaire: age, years of training and service, |
| Interviews | Supervisors and facility managers<br><br>Community representatives | 43 key informant interviews | Background, training responsibilities, weekly & daily pattern of CHWs; resources; successes and challenges<br><br>Perceptions of the programme; acceptability to the community needs; |
| | Follow-up interviews with CHWs' clients who were referred to the clinic during observations of household visits | 74 household interviews | Client's perception of the service, and events subsequent to the referral |

[1] FGD: focus group discussion.

Fieldworkers observed CHWs and their supervisors throughout the workday, and wrote detailed daily field notes guided by a template. After each day spent in the field, the fieldworkers spent a day in the office typing up field notes in full. Each CHW or supervisor was observed for 3-5 consecutive days (interrupted by the office days) to allow for reduction in the Hawthorne effect. Participants were randomly



For peer review only

For peer review only - http://bmjopen.bmj.com/site/about/guidelines.xhtml

selected, although changes were sometimes required due to CHWs taking leave or being absent. Participants responded well to observation, and relaxed substantially during the course of the first day of observation, although there is some evidence the CHWs conducted more household visits early on in the observations.

All available CHWs participated in the FGDs, and all supervisors and facility managers (except one) were interviewed. Community representatives were purposively sampled. Key informant interviews were recorded and transcribed. Finally, 74 household members, who received referral advice from CHWs during observed home visits, were interviewed a month later, to understand their experience of the CHW service, and whether referral advice was acted upon. Interviews were conducted in participant homes, in the participant's choice of language(s), and recorded with a digital recorder. Field workers used the recording to draft a summary of the interview in English.

**Data analysis**

Taking a case study approach, we drew data from the various sources to develop an explanatory description of each team, and then drew comparisons across teams. This process involved several steps. Firstly, we extracted data from each interview or



For peer review only - http://bmjopen.bmj.com/site/about/guidelines.xhtml

day's observation into a template, either by summarising descriptions of events, or extracting raw data such as useful quotations. This increased our familiarity with the data and allowed us to reduce its volume significantly. Multiple team members extracted data from the same sources, compared extracted data and modified extraction strategies until we were confident about inter-extractor reliability.

Secondly, the authors presented a brief summary of each site in a one-day workshop. Through this collective process we identified themes that revealed the similarities and differences across the sites, such as weekly and daily pattern of activities, resources available, record keeping, managing patient referrals to the clinic, engagement with clinic staff, relationship between CHWs and supervisors, relationship with patients, local NGOs, and key community stakeholders. We then generated a template into which we collated site-specific data under these themes, producing a summary for each site (see example in appendix).

In preparing the manuscript, we continued to revisit the raw and summarised data to confirm descriptions of the context, enrich the content, provide clarity, and check emergent ideas. The multiple data sources, as well as events documented by



For peer review only - http://bmjopen.bmj.com/site/about/guidelines.xhtml

multiple field workers, allowed triangulation that increased the validity of our findings.

**Ethics**

The study protocol and instruments were approved by the University of the Witwatersrand's Human Research Ethics Committee (Medical) (M160354), the Gauteng Provincial Health Research Committee in South Africa, and the Biomedical & Scientific Research Ethics Committee (BSREC) at Warwick University (REGO-2016- 1825), United Kingdom. All participants gave informed consent. When accompanying CHWs into the community, fieldworkers obtained verbal consent from household members, prior to entering any household. There were few refusals.

**Patient and Public Involvement**

Patients were not involved other than as respondents. Feedback has been given to provincial, district and facility management, as well as CHW supervisors and CHWs in the intervention sites in various forms, including facility level workshops.



For peer review only - http://bmjopen.bmj.com/site/about/guidelines.xhtml

## RESULTS

### CHW conditions of work across districts

The lack of integration led to work conditions that were demotivating for CHWs. They were not formally employed by the government, but were an outsourced workforce, managed by a private payroll company, and paid a minimal stipend for 6 hours work a day. They struggled to contact the private payroll company responsible for monitoring attendance and paying stipends. An electronic system requiring CHWs to clock in/out at the clinic limited their ability to reach outlying areas as they needed to travel between the clinic and these areas on foot. Two months prior to the start of our fieldwork CHWs had been on strike over their conditions of employment. Moreover, opportunities for career development into nursing were limited, particularly for those without a high school diploma.

CHW work was hampered by insufficient provision of logistical support due to the lack of integration. They often worked without necessary resources, including equipment, stationery, uniform, name badges, or funds for transport or communication. For example, a team of 20 CHWs shared 1 or 2 blood pressure machines borrowed from the facility. When equipment, such as a glucometer, was provided, the associated consumables such as glucose strips were often not



For peer review only - http://bmjopen.bmj.com/site/about/guidelines.xhtml

replenished. Notebooks used by CHWs to record daily activities were purchased out of their own stipend. The general lack of resources compromised CHWs' work and generated resentment.

While teams based in health posts had some dedicated space, clinic-based teams did not have sufficient space to meet, complete paper work or store their files; often files were kept at home. Supervisors did try to address these deficiencies, and some of the endeavours appeared to be morale boosting. CHWs reported that when one supervisor negotiated with the facility manager to use a meeting room for CHW meetings, it immediately increased their job satisfaction.

*"Before she (PN) came, we were like orphans, we had no space inside the clinic. We were doing everything outside. She came with a lot of changes around here…PN had played an important role in our lives…I even enjoy my work now." (CHW-FGD, 1A)*

**The supervisors**

The supervisors in our study sites were either senior (ie. professional) or junior (ie. enrolled) nurses. A professional nurse (PN), with a 4-year degree in nursing, is able



For peer review only - http://bmjopen.bmj.com/site/about/guidelines.xhtml

to diagnose patients, prescribe treatment and dispense medication. Importantly, the PN supervisors in the study site were trained in primary health care and community nursing, and had attended various other courses on TB, HIV, diabetes and hypertension, integrated management of childhood illnesses (IMCI), nursing management and leadership. Many were rehired retirees[1], who had more than 30 years of experience as a nurse before they joined the programme (Table 3). Enrolled nurses (EN) have completed a 2 year course and can provide nursing care under supervision of a PN. All ENs except one (site 3B) received 1-week induction training on the WBOT programme before joining. Some of the EN supervisors in the site had also attended courses on early childhood development (ECD), TB, prevention of mother to child transmission (PMCT), and the expanded immunisation programme (EPI). Most of the ENs were younger and less experienced in community work than the CHWs they supervised. The PN supervisors reported to the facility manager, as did the EN supervisors, if there was no PN supervisor in the team.

**Table 3. Supervision of CHW – who, what and when**

| SUPERVISION AND LOCATION OF TEAMS | PN / EN clinic-based | | PN / EN health post-based | | EN clinic-based | |
|---|---|---|---|---|---|---|
| | 1A | 1B | 2A | 2B | 3A | 3B |
| <u>Who supervises and is supervised</u> | | | | | | |
| **PN supervisor** | | | | | | |
| Age (years) | 65 | 72 | 66 | 59 | n.a. | n.a. |
| Years as nurse | 36 | >30 | 38 | 39 | n.a. | n.a. |
| Years in programme | 4 | 4 | 4 | 4 | n.a. | n.a. |
| **EN supervisor** | | | | | | |



For peer review only - http://bmjopen.bmj.com/site/about/guidelines.xhtml

| SUPERVISION AND LOCATION OF TEAMS | PN / EN clinic-based | | PN / EN health post-based | | EN clinic-based | |
|---|---|---|---|---|---|---|
| | 1A | 1B | 2A | 2B | 3A | 3B |
| Number of JN in team | 2 | 2 | 1 | 1 | 1 | 1 |
| Mean age (years) | 43 | 29 | 28 | 25 | 36 | 31 |
| Mean years as nurse | 14 | 2.5 | 0.5 | 3 | 5 | 2 |
| Years in programme | 0.6 | 0.6 | 0.6 | 0.6 | 0.3 | 0.3 |
| **CHW** (supervisee) | | | | | | |
| Number of CHW in team | 16 | 17 | 9 | 12 | 14 | 20 |
| Mean age in years (range) | 38 (26-53) | 39 (25-47) | 34 (27-45) | 37 (26-51) | 42 (23-58) | 33 (23-54) |
| Mean years (range) as CHW | 7 (4-12) | 7 (3-16) | 7 (6-9) | 6 (3-12) | 10 (3-9) | 6 (5-17) |
| **Formal training of CHWs** | | | | | | |
| % CHW received phase 1 training | 91% | 93% | 100% | 90% | 5.6% | 16.7% |
| % CHW received phase 2 training | 91% | 21% | 100% | 46% | 0% | 0% |
| **Supervision activities** | | | | | | |
| **Supervised home visits (day/week)** | | | | | | |
| By professional nurse | n.o. | n.o. | 1 | Occasional | n.a. | n.a. |
| By enrolled nurse | 3 | 4-5 | 4-5 | s.s. | 4 | On request |
| **On-the-job training** | Yes | Yes | Yes | Yes | n.o. | s.s. |
| **Regular debriefing** | Daily | n.o. | Daily | Weekly | s.s. | s.s. |
| **Examining daily logs and registers** | Yes | Yes | n.o. | Yes | n.o. | n.o. |
| **Assisting in reporting** | Yes | Yes | n.o. | Yes | n.o. | n.o. |
| Frequency of preparing reports | Weekly | Monthly | Monthly | Weekly | Weekly | Monthly |
| **Resolving administrative matters** | Yes | Yes | Yes | n.o. | n.o. | n.o. |

Notes: n.a.: not applicable; n.o.: not observed & not indicated in other information sources; s.s.: the activity was mentioned by a single source other than CHWs, but was not indicated in other data sources.

## Supervisor activities

### *Formal and informal (on-the-job) training*

In four of the six sites, 90~100% of the CHWs had completed the first phase of the national training programme, but only 3 sites (all with PN supervisors) had a significant number of CHWs who had passed the second phase of training (Table 2). Both phases require a written test, followed by a practical assessment conducted by the supervisor during household visits; success in this assessment requires a competent supervisor, and comprehensive and on-going training. One PN placed considerable emphasis on training. *"When PN came, she checked who had done what course and organized training for those who needed it."* (*CHW-FGD, 1A*).

For peer review only



For peer review only - http://bmjopen.bmj.com/site/about/guidelines.xhtml

This PN also used daily morning meetings as training sessions, *"our PN is teaching us … every day in the morning." (CHW-FGD, 1A)*. Given the volume of information that CHWs were required to retain, these regular sessions were important. This PN involved the two ENs in the training: *"Sister teaches us like nobody's business" (EN, 1A)*, enabling the ENs to take up the supervisory role.

In contrast, in the EN-only sites, supervisors were unable to arrange formal training for the CHWs, in part due to their rural location at some distance from the district training centre, and because the ENs were not sufficiently skilled to carry out the practical assessment. With little experience themselves, they were also unable to provide on-the-job training.

*Supervised home visits*

ENs routinely accompanied different pairs of CHWs on home visits several days a week. Some ENs demonstrated sensitivity in correcting CHWs' practice. *"When I can see this one didn't do it right, I keep quiet in the house, but immediately after we step outside as we are walking I do on-the-spot training" (EN, 1B)*. Other ENs working without a PN supervisor, seemed uninvolved during the home visits. Despite one EN spending 4 days a week out in the community, the CHWs did not



For peer review only - http://bmjopen.bmj.com/site/about/guidelines.xhtml

acknowledge her as a supervisor, *"When we get there she's doing the same job that I normally do when I am alone." (CHW-FGD, 3A)* Consequently, her CHWs didn't report problems they encountered to her, such as suspected TB patients, as they had little trust in her problem solving capability.

<u>*Regular debriefing*</u>

One health post-based PN/EN team (2A) held daily debriefing sessions, at which CHWs described the households visited, problems encountered and actions taken. The sessions strengthened CHWs' knowledge base and problem-solving abilities, and provided a platform for collective supervision, enabling the CHWs to learn from each other's experience.

One team (1A) held debriefings in conjunction with other activities, such as in-service training or medication preparation, which diluted the effects of debriefing. In other teams, feedback was said to happen once a month, but we were unable to validate this, and difficulties with recall of events for a whole month would likely limit the utility of this approach. Those teams that had no place to meet inside the facility tended not to discuss serious matters for reasons of confidentiality.



For peer review only - http://bmjopen.bmj.com/site/about/guidelines.xhtml

*"Sometimes we stand outside to hold meetings… patients and the security personnel will be listening to what we are discussing." (CHW-FGD, 3A)*

The CHWs used notebooks to document their daily activities. Through reviewing these notes, experienced supervisors could discuss the visits with the CHWs, ask questions and provide support, as observed in teams 1A, 1B and 2B. In every team there were CHWs that took poor notes, but this was better contained by supervisors willing to spend time reviewing records, and discussing problems with the relevant CHWs. It sent a clear message that documentation of activities was central to the work.

*Assistance in compiling reports*

PNs of two teams (1A & 2B) required CHWs to compile their statistics every Friday, as doing it on a weekly basis (rather than monthly) reduced recall error and thus enhanced quality. One PN verified the weekly report of each CHW against their notebooks, correcting mistakes and making sure that the CHWs understood the meaning of each data category and how notes were translated into figures. This practice strengthened the ability of CHWs to record their work, and trained them to be accountable for what they did. To make sure that CHWs reported faithfully,



For peer review only - http://bmjopen.bmj.com/site/about/guidelines.xhtml

another supervisor would make unsolicited household visits, *"Sometimes I would just go out, and when they talk about households, I would say, oh that one who lives in that shack? And they would say, 'Sister you went there?' I would say, Yes, it is necessary."(SN, 2B)* In contrast, EN supervisors without senior support, tended to simply accept whatever reports the CHWs handed over, with no discussion or interrogation of the statistics produced.

For peer review only

*Clinic duties*

There was an understanding between district officials and facility staff that clinic-based supervisors were to spend 70% of their time on CHW duties, and assist clinic staff in the remaining 30% of their time, particularly if the clinic was short staffed. District officials regularly restated this 'rule', as they were concerned about supervisors neglecting their CHW duties. We did observe EN supervisors, who didn't have PN supervisors, spending substantial amount of time in the clinic, but this was because they felt unable to contribute to CHW activities. However, one EN with PN supervisor said:

*"if I am not busy I help with vital signs stations… it is not part of my job but I do it anyway … I refer my patients and the nurses see them. So there is teamwork. The*



For peer review only - http://bmjopen.bmj.com/site/about/guidelines.xhtml

*support system is great so I never felt pressure to leave my WBOT duties."* (EN interviews, *1B*)

*"Remember, (patients) in this family planning (unit of the clinic), are also the children of the mothers and the fathers that we deliver the medication at home. So, we're working hand in hand.…even if they (clinic staff) ask, are you finished what you were doing? If you don't mind, can you please help me to take a blood pressure on this patient? Then I will do.*" (*EN interview, 1A*)

**The consequences of supervision**

*Motivation, job satisfaction and engagement*

Job satisfaction, professional confidence and motivation were more evident in teams with effective supervision. One CHW commented that since the PN arrived: *"we started to feel that we are now CHWs and human beings." (CHW-FGD, 1A)* Two ENs supervised and mentored by PNs, explained: *"At first it was challenging for me leaving the hospital and working in the community… maybe I'm not a nurse anymore, but now I feel more useful. The community is aware of us … they are coming to us. People are taking treatment now. I think there is lesser tracing on ARV patients, most defaulters you will find are TB. Our stats are going up" (EN,*



For peer review only - http://bmjopen.bmj.com/site/about/guidelines.xhtml

*1B) "I'm a street nurse…but you know what, when I reach the households, this is very enjoyable." (EN, 1A)* When CHWs were on strike over issues with their contract in 2016, some CHWs at the clinic-based PN/EN site (1B) continued working, not wanting to let their patients down or damage their relationship with supervisors and the facility. Their supervisor interpreted their actions: *"they see that you, as their leader, are supporting them and trying to understand where they come from, especially with this employer thing being mixed up. That is why I am saying, hence, they refused to go on strike today." (EN, 1B)*

However, in the absence of supportive supervision, insufficient training constrained CHWs ability to assist patients, and as a result, demotivated them. Their frustrations over their working conditions were unresolved, and sometimes unacknowledged by their immediate supervisors. Some CHWs decided to work fewer hours a day as a passive protest, in addition to participating in the collectively organised strikes.

*Performance*

CHWs with engaged and skilled supervisors carried out a fuller range of activities, and used their discretion to make decisions. For example: *"A patient had an elevated blood glucose level. However, it was too late for the patient to go to the*



For peer review only - http://bmjopen.bmj.com/site/about/guidelines.xhtml

*clinic, so rather than writing a referral letter, she visited the patient again with glucometer after work, by which time the glucose level had dropped by 50%. The next morning, she went for a third time and found that sugar level had returned to normal." (Observation notes, 1A*) This CHW, from a clinic-based PN/EN team, was able to interpret the glucometer readings, and re-checked the patient to avoid making an unnecessary referral.

The dedication of some CHWs to their work is evident in the following client interview: *"If you send a message to a CHW, it does not matter what time it is, or what the problem is, they will come and help you. Whatever problem you have, you can talk to them, and you are not worried that they will be talking about your problem around the area. I like the work they do very much." (Patient-BT, 2A)* In the EN-only model where supervision was the weakest and support from facility staff was negligible, CHW activities were minimal, with CHWs often only delivering medication to the elderly. One CHW highlighted how a colleague's poor performance impacted on the collective reputation of the team, *"It's not good if the patient comes here and when one of the clinic staff checks the card finds out the medication delivery is long overdue. It affects all of us in the eyes of the clinic staff and the PN." (CHW-FGD, 1A)*



For peer review only - http://bmjopen.bmj.com/site/about/guidelines.xhtml

<u>*Collaboration between CHWs and facility staff*</u>

Located at a distance from the main clinics, health post teams received little supervisory support from clinic staff. Other than supplying medication, there was little managerial oversight by the clinic. *"I am not directly supervising them except for sometimes when they are there. You can only supervise something that you can see. With the CHW team it is just via the phone and then it ends there." (Facility Manager, 2A)* In the health post located in a community without a clinic, the PN spent a considerable amount of time seeing patients, and so neglected her CHW supervision responsibilities. Furthermore, the distance between the health post and the clinic hampered efforts to ensure CHWs' patients accessed to care at the clinic.

*"The saddest part is when the patient comes to the clinic and the file is not there, the doctor does not see the patient, and the patient is getting upset with us. Every time when we go to the clinic, we have to look for the files, the clerks do not assist us and we don't even find those files."(Observation notes from conversation with CHW, 2A)*



For peer review only - http://bmjopen.bmj.com/site/about/guidelines.xhtml

In clinic-based team, without a senior supervisor to assist, management of files was also a problem:

*" Sometimes you find the admin clerk is in a bad mood, she speaks to us in a bad way in front of the patients, "I am not going to give you the file. You have to go back to the patient to tell the patient to come to the clinic to collect it." You report to the sister, she will tell you that she has been warning [the admin clerk] for several times but she does not listen." (CHW-FGD, 3A)*

CHWs reported that other facility staff expressed a dismissive attitude towards them:

*"the peer educators, HIV/AIDS counselor we all go together to sign the same contract but they are treated as if they are more educated than us, they call us street maids" (CHW-FGD, 3B)*

When asked why they were not allowed to work inside the facility, the CHWs responded: *"the manager tells us that we are not part of the clinic, so there's nothing she can do for us."* (field notes, 3A) The denial of sharing space exacerbated the CHWs' sense of marginalisation and exclusion.



For peer review only - http://bmjopen.bmj.com/site/about/guidelines.xhtml

However, in clinic-based teams, with the support of senior supervisors, and the resulting growth in skills and confidence, CHWs had been able to establish better relationships with clinic staff. In turn, clinic staff, seeing the CHWs' greater motivation and effort, were more likely to treat the CHWs with respect. There were several benefits to this resulting collaboration. Firstly, communication and coordination was better. Working together, the facility manager and PN were able to confirm whether, once defaulters had been traced by CHWs, they returned to the clinic. *"We engage with facility manager, she helps with problems that we have... we include her in everything" (EN, 1B)*. Clinic staff willingly accepted referrals from CHWs, and included the CHWs in training sessions: *"Sister X is not (in the CHW team), but she is very helpful. She attended severe malnutrition training and she called all the CHWs and did a presentation for everybody." (EN, 1B).*

Secondly, in one clinic, the contribution of CHWs had become indispensable: *"If they (CHWs) were not there, it means you will be over worked, the clinic will always be full, to be honest. There is good communication between us." (Facility Manager, 1A)*



For peer review only - http://bmjopen.bmj.com/site/about/guidelines.xhtml

The third benefit was the empowerment of CHWs, who learned to negotiate with other less willing staff: *"I have a patient who doesn't want to come to the clinic and I don't want my patient to default on her HIV treatment. I said to the sister 'my patient is working at the farms so please pack medication for her. I'll ask her to come next month.' The sister scolded me because the patient did not come on her last appointment. So I begged the sister and promised to bring the patient myself. Then it was sorted, the patient got her medication and she came to the clinic with me." (CHW-FGD, 1B)*

*Negotiating acceptance and building the community's trust*

All CHW teams depended on the supervisor to liaise with key community members:

*"We established good relations with the ward councillor so whenever there was a problem I would call her." (PN, 2B)* The presence of the senior supervisor increased CHWs' credibility in the community: *"If CHWs found that I am not well, they report to the PN, and the PN would come and see me. I have noticed that when I follow the PN's advice, my health improves. We are alive today because of them." (Patient-BT, 2A)*



For peer review only - http://bmjopen.bmj.com/site/about/guidelines.xhtml

Box 1 describes visits to two different households conducted on the same day by the same CHW, for which she received very different support from the facility.

**Box 1: Support from the clinic facilitates CHW performance and client trust**

*Case 1 : Patient defaults as nurse insists on waiting for test results*

The patient, a woman on ARV with a spinal cord problem, had moved recently. She was no longer able to collect ARVs and go for physio appointment at the clinic near her previous home as she had no money for transport. She asked for a referral to a closer clinic. The nurse refused as ARV patients tended to move clinics, so patient stole the file.

The CHW took her file to the closer clinic to ask if she could collect medication for the patient. The CHW was told that a new file had to be opened and the patient had to wait for CD4 results to come back. As a result, when we interviewed her, the woman had been defaulting for the past 3 weeks.

In the interview the patient said the CHW had not followed up on her requests for food parcels as they had no food, for a physio appointment, or for her husband to be tested for HIV. *"The CHW doesn't follow up on me to see how I am doing; the only thing that she is good at is making empty promises." (Patient-AA, 3A)*

*Case 2: Social worker responds to CHW request*

The patient was a woman whose child was knocked down and killed by a car in front of her and her children. The family were traumatised. CHW wrote a referral letter to a social worker. The patient went to the social worker and then started attending a support group. CHW also arranged for the social worker to see her husband and children at home.

Patient said she trusted CHW because *"she is kind, open and she knows how to speak to people even if you are in a difficult situation. She does not gossip about her patients on the street." (Patient-Z, 3A)*

Inadequate and unpredictable support from the clinic profoundly influenced the CHW's ability to provide care for her clients, and in turn, the degree of trust between client and CHW.



For peer review only - http://bmjopen.bmj.com/site/about/guidelines.xhtml

464

465 Table 4 summarises the key effects of location and the level of supervision on CHW
466 performance.

467

468 **Table 4: Impact of supervision and location on the determinants of CHW performance**

| | Clinic-based with PN and EN | Health post with PN and EN | Clinic-based with EN only |
|---|---|---|---|
| **Mentorship of EN and CHW** | Good | Depending on how busy the PN was seeing patients | • Junior ENs were not able to provide adequate training or supervision<br>• Little mentoring as facility manager was not engaged; |
| **Relationship with clinic** | Good | Poor | Poor |
| **Files** | Well managed by PN | Files at clinic were not managed by PN & EN; often lost and CHWs had to go and find them | Part of clinic filing system |
| **Effect on quality of service** | • More able to ensure that patients get care needs;<br>• Better relationship with community | • Limited service at health post; patient had to travel to clinic for anything other than basic service;<br>• Referrals were more difficult because of distance between health post from clinic; | • CHWs shied away from difficult cases, knowing that they did not have the support;<br>• Poorer relationship with community |

469

470



For peer review only - http://bmjopen.bmj.com/site/about/guidelines.xhtml

## DISCUSSION

In this paper, we explored the effect of three different supervision and location configurations on CHW motivation and performance. We have demonstrated that experienced supervisors employed a range of strategies to train, motivate, and monitor CHWs, to improve the quality of their work. In turn CHWs were treated with greater respect by the clinic staff, making space for collaboration and support that benefited both parties. Being a near-equal of the facility manager, a PN supervisor was in a position to bridge the divide between the CHWs and clinic staff. When one of the PNs was able to obtain a place for the CHWs inside the facility, she secured a position – physical and symbolic – for them in the health system. She drew in other staff to participate in the supervision of CHWs, further defending their legitimacy. Clinic staff provided advice and ensured the CHWs' patients accessed the care that they needed. CHWs traced the defaulting patients in the clinics and so improved the clinic's patient statistics. The senior supervisor, a shared resource to both the CHWs and the clinic, was able to effectively integrate the CHWs into the health system.

In contrast, the teams in health posts found it difficult to build relationships with the clinic, particularly if the health post was at some distance from the clinic and the PN



For peer review only - http://bmjopen.bmj.com/site/about/guidelines.xhtml

was busy with patient consultations. The EN-headed teams lacked adequate training and supervision, and consequently the CHWs' performance was minimal. With little evidence of the CHWs' contribution, the social distance between the CHWs and clinic staff remained substantial. Given her junior position, the EN was not able to facilitate a collaboration with the clinic to reduce this distance. Consequently, despite being based in the clinic, the EN-led CHW teams were not effectively integrated into the health system.

Our study has several limitations. Firstly, despite fieldworkers asking CHWs to carry out their duties as normal, during observation many CHWs appeared to make extra effort to reach a greater number of households. We followed the same CHWs for 3-5 days to mitigate this, and the number of household visits did fall overtime, as the CHWs became used to the presence of an observer. Secondly, our data on CHW performance is based on what we learned from our respondents rather than on an objective measure of performance. Thirdly, while we selected study sites to ensure that the different supervisory configurations were included, we do not know to what extent the included CHW teams are representative of other CHW teams in the same district. The study demonstrates noteworthy strengths. As far as we know, this is the first study to examine the effect of different levels of on-site supervisors



For peer review only - http://bmjopen.bmj.com/site/about/guidelines.xhtml

509 as well as the location on CHW performance in a government programme. The case study approach allowed us to purposively select teams based on their different supervision/ location configurations, and to compare and contrast their functioning in order to understand what worked, and what did not.

CHW supervision is often no more than periodic interactions by facility staff or officials who may not understand the context and role of CHWs. This can result in bureaucratic inspection and fault-finding, rather than problem solving and support.[6, 7, 20] For example, in the Ethiopian national programme, supervisors make irregular visits from either the local health centre or district office.[21] In our study, the presence of an appropriately skilled and qualified on-site supervisor enabled on-going supportive engagement, rather than a distant supervisor who had fit supervisory visits into a busy schedule. Evidence from a rapid assessment of the South African WBOT programme in five provinces suggested that the existing facility-based staff were unable to provide effective supervision as they were already overstretched, working in under-resourced and under-performing clinics. [15] The authors concluded that CHW teams should be based in stand-alone health posts with a nurse supervisor, rather than within a clinic. Our data suggests it would be more advantageous for the nurse supervisor and the CHWs to be based at a



For peer review only
For peer review only - http://bmjopen.bmj.com/site/about/guidelines.xhtml

clinic, with clear rules about how much time the supervisor can spend on clinic activities.

In the Ethiopian programme, CHWs that were based in health posts also struggled to make referrals and experienced dismissive attitudes from clinical staff; while where there was more regular contact, the CHWs felt valued and part of the team.[21] Studies in Uganda, Bangladesh and Bolivia have reported that contact with a higher level of the health system gave CHWs credibility with their clients.[22-24] Similar to our findings, in Mozambique supervision increased the participation of CHWs in the intervention facilities.[13]

Training and supervision of supervisors is a more neglected subject than that of CHWs.[9, 25, 26] This issue is beyond the scope of this paper, but in the second phase of our 3-year study, we are evaluating the effect on performance of a PN supervisor in building the capacity of the EN supervisors.

Globally, there are on-going efforts to scale-up CHW programmes as a means to strengthen health systems and accelerate universal health coverage. These include the call for one million CHWs in sub-Saharan Africa,[27] making CHWs available



For peer review only - http://bmjopen.bmj.com/site/about/guidelines.xhtml

everywhere on the African continent, [28] developing guidelines by the World Health Organization (WHO), as well as the latest funding commitment from the private sector.[29] Our study suggests that it is worthwhile to make senior supervision an explicit component of CHW programmes. It takes funds, understanding of context specificity, as well as innovation, to implement cost-effective supervisory mechanisms. This research has offered some recommendations based on the experience of well-functioning teams (See Table 5).

For peer review only



For peer review only - http://bmjopen.bmj.com/site/about/guidelines.xhtml

**Table 5: Programme design features and supervisory strategies that worked**

| | Recommendation | Rationale |
|---|---|---|
| Programme design | Attachment to PHC clinic | Facilitate physical & operational integration into the health system |
| | Team up senior and junior supervisors | Build relations, guide CHWs to navigate through the community and system, pass down know-how to junior supervisors |
| | Setting guidelines as to how much time supervisors can spend assisting in the clinic | Help to acknowledge that the engagement is a two way collaboration, in which there are benefits for everybody |
| | Strengthen HR management practices | Build trust, improve dialogue in the workplace, problem solving, supervision, and culturally appropriate communication |
| Supervisory strategies | Supervise home visits | Provide opportunities to strengthen CHWs' knowledge and skills, demonstrate a strong backup for CHWs in the community, keep updated of community's status |
| | Formal and on-the-job training | Impart knowledge and skills |
| | Regular debriefing/feedback | Individual and collective supervision, track performance, build up teamwork spirit |
| | Examine daily logs and registers | Ensure accurate documentation and subsequent reporting |
| | Direct CHWs to tasks, such as tracing defaulting patients, that explicitly assist the clinic | Ensure the clinic staff are able to see the benefits of having CHWs as part of the team |
| | Draw in clinic staff to work with and train CHWs | Improve the extent and quality of working relationships between clinic staff and CHWs, and allow CHWs to benefit from the clinic staff's expertise |
| | Assist data collating & reporting | Ensure that CHW activities are accurately reported so that health system managers can see the benefits of the programme |
| | Administration and logistics | Resolve administrative matters, negotiate for better work conditions, ensure CHWs are adequately equipped to deliver service |



For peer review only

For peer review only - http://bmjopen.bmj.com/site/about/guidelines.xhtml

## CONCLUSION

With high expectations of CHW programmes in improving access to care in South Africa and other similar settings, full-time, on-site supervision can make an important contribution in enabling CHWs to provide comprehensive, promotive and preventative care. In the context of an overstretched health system, senior supervision located within a clinic can enable the development of a collaboration between the clinic and CHWs, reducing the CHWs' marginalisation, and enabling their potential contribution to improving health outcomes to be realised.

For peer review only

### Contributorship

YHT interpreted data, wrote and revised the manuscript with JG, and approved the final manuscript as submitted.

FG and JG conceptualized and designed the study, analysed data, critically revised the manuscript and approved the final manuscript as submitted.

JDK carried out data collection process, managed data, analysed data, critically revised the manuscript, and approved the final manuscript as submitted.

NN analysed data, revised the manuscript and approved the final manuscript as submitted.

TR and HM analysed data and approved the final manuscript as submitted.

### Acknowledgements

The authors would like to acknowledge the following individuals for their invaluable contribution: the field workers, the respondents, the CHW teams, Mrs Salamina Hlahane, district manager at Sedibeng, and Mrs Bridget Lefhoedi, community-based services coordinator. Input from Professors Margaret Thorogood,



For peer review only - http://bmjopen.bmj.com/site/about/guidelines.xhtml

David Sanders, and Emmanuelle Daviaud throughout the study has been highly appreciated. We also thank these organizations for their generous support: Department of Health Sedibeng District, Gauteng Department of Health, the UK Medical Research Council (MRC) and Taiwan Ministry of Science and Technology.

**Endnotes:**

Re-hired retirees were used for three reasons. Firstly, due to the shortage of professional nurses in the system, drawing in those who had retired increased the supply. Secondly, they had a wealth of experience in community nursing, which younger nurses often did not. Thirdly, they were given an increment on top of their pension, rather than a full salary, and so cost the district less than a formally employed professional nurse.

**Competing Interest**

None.

**Data sharing**

A sample of site-specific summary is available in appendix.

**Funding**

MRC-UK under Health Systems Research Initiative (MR/N015908/1)

Taiwan Ministry of Science and Technology (105-2917-I-564-006)



For peer review only - http://bmjopen.bmj.com/site/about/guidelines.xhtml

## REFERENCES

1. Perry HB, Zulliger R, Rogers MM. Community health workers in low-, middle-, and high-income countries: an overview of their history, recent evolution, and current effectiveness. *Annu Rev Public Health* 2014;35:399-421.
2. Datiko DG, Yassin MA, Theobald SJ, et al. Health extension workers improve tuberculosis case finding and treatment outcome in Ethiopia: a large-scale implementation study. *BMJ Glob Health* 2007;2**:**(4)e000390.
3. Lehmann U, Sanders D. Community health workers: what do we know about them. The state of the evidence on programmes, activities, costs and impact on health outcomes of using community health workers. Geneva: World Health Organization 2007:1–42.
4. van Ginneken N, Lewin S, Berridge V. The emergence of community health worker programmes in the late apartheid era in South Africa: An historical analysis. *Soc Sci Med* 2010;71:1110-8.
5. Barnett ML, Gonzalez A, Miranda J, Chavira DA, Lau AS. Mobilizing Community Health Workers to Address Mental Health Disparities for Underserved Populations: A Systematic Review. *Adm Policy Ment Health* 2017;1-17.
6. Hill Z, Dumbaugh M, Benton L, et al. Supervising community health workers in low-income countries–a review of impact and implementation issues. *Glob Health Action* 2014;7:24085.
7. Glenton C, Colvin CJ, Carlsen B, et al. Barriers and facilitators to the implementation of lay health worker programmes to improve access to maternal and child health: qualitative evidence synthesis. *Cochrane Database Syst Rev* 2013;10.
8. Kok MC, Kane SS, Tulloch O, et al. How does context influence performance of community health workers in low- and middle-income countries? Evidence from the literature. *Health Res Policy Syst* 2015;13:1-13.
9. Pallas SW, Minhas D, Pérez-Escamilla R, et al. Community health workers in low-and middle-income countries: what do we know about scaling up and sustainability? *Am J Public Health* 2013;103:e74-e82.
10. Kok MC, Dieleman M, Taegtmeyer M, et al. Which intervention design factors influence performance of community health workers in low-and middle-income countries? A systematic review. *Health Policy Plan* 2014;30:1207-27.



For peer review only - http://bmjopen.bmj.com/site/about/guidelines.xhtml

11. Bosch-Capblanch X, Liaqat S, Garner P. Managerial supervision to improve primary health care in low- and middle-income countries. Cochrane Database Syst Rev 2011;9.

12. Rabbani F, Shipton L, Aftab W, et al. Inspiring health worker motivation with supportive supervision: a survey of lady health supervisor motivating factors in rural Pakistan. *BMC Health Serv Res* 2016;16:397.

13. Madede T, Sidat M, McAuliffe E, et al. The impact of a supportive supervision intervention on health workers in Niassa, Mozambique: a cluster-controlled trial. *Hum Resour Health* 2017;15:58.

14. Ameha A, Karim AM, Erbo A, et al. Effectiveness of supportive supervision on the consistency of inter-grated comunity cases management skills of the health extension workers in 113 districts of Ethiopia. *Ethiop Med J* 2013;52:65-71.

15. Marcus TS, Hugo J, Jinabhai CC. Which primary care model? A qualitative analysis of ward-based outreach teams in South Africa. *Afr J Prim Health Care Fam Med* 2017;9:1-8.

16. Schneider H, Schaay N, et al. The challenges of reshaping disease specific and care oriented community based services towards comprehensive goals: a situation appraisal in the Western Cape Province, South Africa. *BMC Health Serv Res* 2015;15:436.

17. Department of Health, South Africa. Provincial guidelines for the implementation of the three streams of PHC Re-engineering. http://www.jphcf.co.za/wp-content/uploads/2014/06/GUIDELINES-FOR-THE-IMPLEMENTATION-OF-THE-THREE-STREAMS-OF-PHC-4-Sept-2.pdf (accessed 23 Jan 2017).

18. Trafford Z, Swartz A, Colvin CJ. “Contract to Volunteer”: South African Community Health Worker Mobilization for Better Labor Protection. *New Solut* 2018; 27:648-66.

19. Nxumalo N, Choonara S. *A rapid assessment of ward-based PHC outreach teams in Gauteng Sedibeng district–emfuleni sub-district.* Johannesburg: Centre for Health Policy, University of the Witwatersrand, 2014.

20. Roberton T, Applegate J, Lefevre AE, et al. Initial experiences and innovations in supervising community health workers for maternal, newborn, and child health in Morogoro region, Tanzania. *Hum Resour Health* 2015;13:19.

21. Kok MC, Kea AZ, Datiko DG, et al. A qualitative assessment of health extension workers’ relationships with the community and health sector in Ethiopia: opportunities for enhancing maternal health performance. *Hum Resour Health* 2015:13:80.



For peer review only - http://bmjopen.bmj.com/site/about/guidelines.xhtml

22. Bartos A, Tenorio A, Saunero R, et al., *Extending the duration of exclusive breastfeeding in El Alto, Bolivia through a community-based approach and the provision of health services.* Geneva, Switserland: Child Health and Nutrition Research Initiative, 2009.
23. Nankunda J, Tumwine JK, Soltvedt A, et al. Community based peer counsellors for support of exclusive breastfeeding: experiences from rural Uganda. *Int Breastfeed J* 2006;1:19.
24. Haider R, Ashworth A, Kabir I, et al. Effect of community-based peer counsellors on exclusive breastfeeding practices in Dhaka, Bangladesh: a randomised controlled trial. *Lancet* 2000;356:1643-7.
25. Daniels, K., et al., Supervision of community peer counsellors for infant feeding in South Africa: an exploratory qualitative study. *Hum Resour Health* 2010;8:6.
26. Rowe AK, Onikpo F, Lama M, Deming MS. The rise and fall of supervision in a project designed to strengthen supervision of Integrated Management of Childhood Illness in Benin. *Health Policy Plan* 2009;25:125-34.
27. Singh P, Sachs JD. 1 million community health workers in sub-Saharan Africa by 2015. *Lancet* 2013;382:363-5.
28. The Lancet Global Health. Community health workers: emerging from the shadows? Lancet Glob health 2017;5:e467.
29. Winters N, O'Donovan J, Geniets A. A new era for community health in countries of low and middle income? *Lancet Glob Health* 2018:6:e489-90.



For peer review only - http://bmjopen.bmj.com/site/about/guidelines.xhtml

Case site summary
Site: A Author(s): JG Date:22 02 2017

| | |
|---|---|
| **Model** | Clinic based team, although doesn't perform all standard clinic services (e.g. TB)<br>One PN, two ENs |
| **PN** – background/ experience, age, role, actual responsibilities | Professional nurse, midwifery, community health worker, at X for 6 years then Y hospital, retired at X, then went to the UK for X years .. started to work in Y at 62 .. now 72 years<br><br>**PNO:** once CHW have gone into the field, and have explained several things to the FW, spends an hour seeing patients.. and then leaves (so no meeting after CHW return)<br><br>**PNO_NM:** interview with FW, patient consultations for 2 hours; after 11.20 she reads patient files / notebooks; She also see family planning patients; (FW-PN doesn't seem to have a lot of work to do) |
| **EN**– background/ experience, age, role, actual responsibilities | **<u>EN01</u> - responsible for ward X**<br>**X** years old, became a EN in X, worked in private clinic before here, not from the area<br>FW comment: the EN seems passive.. maybe due to helplessness around CHW.. she struggles to maintain basic level of supervison by checking and doing CHW stats. Not clear how much time she spends in the clinic<br>*JG: from first meeting we know this EN doesn't like going out into the community .. it may be that she prefers to work in the clinic. When it comes to observations, she didn't actually do the stats because EN02 had given it to the data capturer to do because EN02 was going on training .. so sounds as if EN02 does most of the data reporting etc.*<br>**Observation day 1:**<br>Private meeting between PN and EN<br>Check on crèche that children have been immunized – no really confirmation of this<br>HH1: EN manages interaction with hh – follow up<br>Check on a pregnant woman at the crèche: registration done by CHW<br>HH2: patient there, but says that gate is locked;<br>HH3: patient not there<br>HH4: EN leads the interaction – follow up<br>FW: thinks EN went into the field because FW was there to observe .. but not real need for her to be there.<br>**Observation day2:**<br>Works in clinic as they are short staffed – so can't be observed; lunch and then knocks off as ½ day;<br>Next week she is on training so will ask receptionist to help with the stats. She will sign books to show she has checked them<br><br>**<u>EN02</u>** used to work in critical care unit in private hospital<br>Not sure if I am a nurse any more .. but I feel more useful<br>Much more active, tries to solve problems, has set up whats app group, able to provide supervision<br>**Observation day 1:**<br>Creche campaign; EN is leading activities<br>**Observation day 2:**<br>Works in clinic as short-staffed; sorting patients and then doing child immunisations;<br>Comes out to check on CHW to allocate them to FW<br>HH1 CHW leads<br>HH2 CHW checks patient, but EN worried so asks questions about stomach<br>HH3: EN leads<br>HH4: EN asks about granny; in hospital; EN is shocked because here only 2 weeks ago, checks whether she has enough medication etc *(suggests that EN02 does go regularly to the field)*<br>HH5: jointly doing activities<br>HH6: CHW leads, didn't ask about child<br><u>HH7</u>: Jointly leading<br>Return to clinic at about 12 noon<br>*FW comment: EN is very supportive of CHW*<br>**Observation day 3:**<br><u>HH1</u>: on way to support group visited a lady who had given birth to twins- just checked on babies<br><u>Support group</u>: EN sits at a distance, CHW know how to do their work; she only intervened when a BP was too high, and when difficult TB case arrives (HH interview: CHW bring sputum bottle, but patient fails to provide) ; not 12 noon so there is time to see a few households |

For peer review only

| | |
|---|---|
| | <u>HH2</u>: not allocated to a CHW, but she checks on him because he is sick with TB; EN leads (as she hasn't been before); EN says they will come and do registration even though not in demarked area<br><u>HH3</u>: car accident victim and HIV<br><u>HH4</u>: EN quiet, CHW leads<br>Back on counsellors place by 1pm after 30 mins walk<br>**JG:** *EN seems works hard, happy to go and find extra households, seems to know some households – evidence that goes into the field regularly*<br>**Observation day 4**<br>Discussion about working on Fridays they do stats – cHW said we don't; EN said that is only the last Friday of the month .. so today they are going into the field; Later EN says later .. eish we are working on a Friday .. this is slavery<br><u>HH1</u>; came to give referral letter for NGO.. which is now closed for the year;<br>CHw says I will take you to the patient you didn't manage to see on Monday<br><u>HH2</u>: patient discharged from hospital in pain; EN tried to help – offered to call an ambulance, but woman said no because nobody to go with her; suggested clinic on Monday – ok, daughter will take me; asked for daugther's number; can't get it out of the phone<br><u>HH3</u>: checking on patient that they referred two days ago; she hadn't gone ; doesn't take her treatment;child without immunization; EN quite upset<br><u>HH3</u>: healthy baby; EN happy ☺ |
| **CHWs** – average details across team (Number, age, gender, years' experience, education) | X CHWs in FGD, (X reported to part of team) , all female, (another X waiting to be registered. PN wants them back)<br>Average age 39 (range25-49)<br>64% of have some secondary education;<br>29% have matric<br>7 years on average as a CHW (range 3-16)<br>Average number of households=262 (248-317) (only Sicelo and Biopatong gave specific numbers per CHW)<br>25 mins walk from home to clinic (range 2-50)<br>18 mins walk from clinic to households (2-45)<br>93% have phase 1; 21% have phase 2 |
| Community profile (eventually draw in survey data) | **PN:** Ward X contains a large informal settlement **(FDG SM?)**<br>Ward 10 predominately a township **(FDG AS?)**<br><br>**PN:** We started going from house to house, counting people during the registration.. according to where the CHWstayed.. initial plan was 250 households, but didn't work .. either 190 or 200 (if medical aid we skip it)<br><br>People report to us if somebody is sick, they will even break down the door<br>**ENO2** (ward X) – area is huge - shortage of CHW<br>3 areas within ward X<br>Ext X and X informal settlements / shacks – 5 is too big<br>Ext X – RDP house<br>People are moving to Rdp houses, go back 3 days later and people have moved..<br>Lots of Zim patients, but language not a barrier<br>Informal settlement, lots of crime, didn't feel safe at beginning,, but not easy for CHW to be robbed because most of them are well known in community |
| Challenges? (Not general) | **FM:** Storage is a problem – for files,<br>Safety .. too many break ins in the clinic<br>CHW need more training, and then more CHW will hopefully come later<br>**FDG-SM**<br><u>Equipment</u> – one CHW has take the BP machines that side .. so you call her to ask her to bring this side .. call from counsellor .. and we don't have machines<br><u>Behaviour of nurses</u> – some patients refuse to come to the clinic because of the bad treatment. However we encourage them for the sake of their health to ignore the nurses attitude P11<br><u>Queuing</u> – it irritates the patients .. so it gives us a problem when we try to encourage the patients to go to the clinic ..<br><u>Clocking in</u> WBOT promised we would work around where we stay.. they promised a clock in Ward 8 .. we have found a place for it .. it is tiring to have to come to ward 10 to clock in .. go back to ward 8 and then come back to the clinic .. it is a distrance and it is hot or raining!<br>Stipend, job security, career development, smart purse<br>**FDG_AS**: as above plus job safety, training, recognition and appreciation<br>We can't even go to the bank with this card, only to shoprite .. otherwise they charge us |

For peer review only

| | |
|---|---|
| | **EN02 Obs:** currently leave is pause .. because smart purse says 13 days, contract says 21 days – hey have sent a letter to smart purse – EN really interested in solving problems .. but sometimes above her |
| Planning (of the day) | **PN:** I have paired them, an intelligent one with a less intelligent one |
| Weekly pattern of activity (what do they do when?)<br>- overtime | **FM:** one of their strengths is tracing .. they will find the person p18<br>CHW say that they do overtime<br>**FM:** says they don't<br>**FM:** may be ENs help out in the clinic 1 day out of 5<br>Did two campaign days during FW<br>**EN01:**<br>Run support group for elderly patients on Wednesday<br><br>**FDG-SM:**<br>We follow up on defaulters<br>Household registrations each day, - it is not possible to do registrations at one time of the year<br>Home-based care – we don't do much of this ..<br>we refer to social services<br>*JG: not convinced there is a day for this, and a day for that .. rather that they do all of the activities when needed.*<br><br>**FDG-AS:**<br>With Kgatelopele the first week I deliver medication, the second I will check if they are taking it properly by counting.. if it doesn't balance .. then I will ask her why .. then I will report to the clinic to the cinic that I am following up on this person . |
| Daily pattern of activity | 6 hh per day (for two CHW) (average is 5)<br>13 mins average per household (average is 12)<br>10 mins average travelling between households (average is 14)<br>5 hours work per day (average is 4.40)<br><br>5% of activities are meeting with supervisors (not time)<br>20% of activities are HIV as condition; (TB 5.5%)<br>52.75% of activities are other chronic<br>5.49% as mother and child<br>53% of recipients are elderly<br><br>Between 8-9 am do training<br>**EN02**: training and gym until 9am.. e.g. with new machines I take two CHW and show them so that they know what to do in the field<br>You can see a big difference between those who have attended the trainings..<br><br>On a Tuesday they are taking medication to patients, and from there to check on what ever<br>So if we have gone to 2 households, taking treatment, then I pick one house, any house that we haven't been to before ..and do a registration ..<br>Cos you find that their focus is on the patients that they know, the patients that they know<br>But with me I am always squeezing in just one house that has never been registered .. so I don't totally change their routine .. just one HH and you will find problems<br><br>**EN01:** 8am clock in and wait outside until 9am when go to households (no mention of training)<br>CHW work in pairs must see 6 households 3, 3, lunch break is between 12-1<br>They don't just to go their patients, they also visit other households after a longer break when no new patient is identified. People in the area also inform the CHW when there is somebody who is sick;<br><br>**FGD-SM:** 6 households per day each and households registrations is too much .. and we have to be back here by 13.30 .. our lunch is at 12 noon. We discuss issues we have with the nurse and pack medication to deliver (next day or on way home)?)<br>We write everything in our note book .. and then we note in our diary when we have to return to a househo ld<br><br>**FDG-AS:** planning seems to be more about which of your houses and which of mine<br>• Last time they told us we had to see 6 patients a day per CHW .. now they have changed it to 4<br>• If you are doing household registrations .. you can't do 6 per day p13 |

For peer review only

| | |
|---|---|
| | • Sometimes we do home-based care, if the patient is living on her own and she is on treatment . .if ask if she has eaten .. if she has't I cook for her;<br>• We refer them .. the provider needs 8rand<br>• Sometimes we teach the family how to bath them |
| Record keeping & Stats – how & when<br>- How notes/info are kept<br>- How they are consolidated<br>- Patient files<br>- Follow ups | **FM:** Has improved since two ENs arrived<br>WE don't have email and our transport is irregular;<br>Now doing it once a week .. we used to do it once a month .. but then it was late<br>Two ENs are taking responsibility and they forward to the data capturer and then we come back and chat about it (Sister Tenqui and FM?)<br>**PN:** they have notebooks that they write in .. not more than 5/6 households per notebook .. (*so does that mean that they have 40 notebooks?)* I read those books every time I get a chance<br>**EN02:** she does monthly stats, she checks individual stats, talks to CHW saying why do you say this, or why haven't you write x, gives training to CHW.<br>In training we were told it would be easier if we did it weekly so I said to CHW lets do it on Fridays .. lets not go ot households but do stats instead .. now we are doing it monthly because we are faster now<br>From CHW to EN to facility manager<br>*JG: this is why they don't work on Fridays.*<br>**FDG-SM** we do stats daily .. at lunchtime we record evertying (some confusion about recording number of children)<br>**FDG-AS:** the OTL takes time to check them<br>I do them in my lunch hour, or at home, or when we are seeing my partner's patients |
| Referrals<br>- Forms/documentation<br>- Queueing<br>- Files | **FM** :Patient meant to show form at door and then goes to see Sister X, who then completes the form and gives back to the carer (if another nurses see patient they also complete the form)<br>**EN02:** Report number of referrals in stats as well<br>**FDG-SM**<br>"The referral form is causing trouble with the patients. The patients are supposed not to follow the queue, they should go straight to the PN but the patients are been shouted, told to go and follow the queue. Now the patients are fighting with us because we told them that with the referral they don't have to queue."p 15 |
| Medication delivery | **FM:** ENs can't come issue medication; so tengiwe does it . and if she is not here, they come to me with the vital signs data and the pill count, the condition of the patient, and the next clinic data. If the ENs are there the CHW will do this<br>**FDG-AS:** when you count their medication, sometimes they start shouting at you . you try to tell them that I am tyring to help you .. |
| Relationships Clinic – WBOT team | Other sisters will give medication out if necessary<br>CHW refer patients to specific sisters eg. For TB<br>**EN02** We do in-service training .. Sister X is doing EPS .. she is very helpful .. she is not WBOTS but she is very helpful - she is a staff nurse, but she has got more xperience … she attended severe malnutrition training .. she called all the CHW and patients .. shw as doing a presentation for everybody .. so it is not only us doing that .. She is also helping when we need something There is support from the clinic.<br>We engage with the FM for everything, problems we have, reporting .. she is part of the community, we include her in everything<br>District coordinator – I have only seen her once, when she came with those two ladies from wits !<br>Training person at X– if I have aproblem a message the OTL group, or even phone her .. she is giving support.<br>**FDG-SM**<br>"At the clinic we are treated as if we are not part of the clinic, they talk to us as if we are children. They shout at us in front of the patient that you brought for help. We don't have support at all. Another thing, they don't want us to eat breakfast but they eat when they come to the clinic. We consult TB patients on empty stomach. I did raise that to the PN and she said we should eat at home. Then I asked why she is not also eating at home in the morning and see if she can do it. In the morning we prepare our children to go to school, others have husbands to take care of before we leave at 7:00am, and we have to walk. We don't have time to have breakfast at our houses in the morning. We only have breakfast and lunch together at 12:00pm. There is no support at all." P15<br>**FDG-AS:**<br>"the sister in charge and our OTLs are treating us like.. I don't know .. as you can see we have no place to stay, no chairs, no table, nothing .. we came with the idea of starting the day with a prayer like in hospitasl.. but the sister in charge says we won't do such a thing here .. so we stopped because we are powerless' p14<br><br>**Any conflict with OTL or clinic staff** |

For peer review only

| | |
|---|---|
| | "We are all going to talk neh .. add to what I say .. we have our sister in charge, but we have another one .. the CHWs know who I am talking abut .. this sisteris controlling us more than our OTL" .. P21<br>"she even shouts at us infront of the patients. We are adults, mothers and we have our own houses and when we are here she treats us like children. She does not respect us." P21<br>"And sometimes we walk in, she shouts and says 'hey you is that a uniform, that is not the way we dress here.' Where are we going to get the new navy pant? That pant costs R190-R200.00. All that shouting she does it in front of everyone at the clinic. That is not okay and when you talk back to her, she will say that she is not our OTL and does report to us."<br>**Participant**: "We want her to be out of the WBOT team. We want to report to our OTL only. She is responsible for other nurses not us."<br><br>"My challenge as a CHW is that when I do home visit, some households do not have food to eat and the patient is on treatment. So I have to empathise with the patient, contribute something so that the patient can eat. When I come to the clinic and ask for this porridge, the clinic staffs tells me that I have to come with the patient so that they can see the patient. That is a big problem because the patient is sick and has no transport money to go to the clinic. So yah it hurts." P20 |
| Relationship EN & PN | **EN02:** if I need clarity I will phone here |
| Supervision of CHWs (including training) | **PN:** I read their books whenever I can; if they have difficult cases they bring them straight to me .. and I take the car and drive to see the issue ..<br>**EN01:** Most of the CHW are older than her.. they don't always listen to the EN. When they only have one household to visit they have all sorts of stories to get away. She reported this to the FM who said she would sort it out.<br>Two CHW communicate on behalf of the rest ..<br>They report to the EN when they have found a defaulter, they then revisit and then report<br>Weekly or bi-weekly she checks the activities of the CHW<br>FM told CHW not to come to work during strike<br><br>**EN02:** supervises 8 CHW, 5 have not been registered so they are using the book. If she knows CHW have much work to do, she leaves with them at 8 rather than 9<br>Different types of CHW – counsellor, peer educators, rest are CHW; one CHW is the leader whose job it is to forward complaints to her<br>If I have been on training I will ask how did it go with that patient, we have an whatspp group so we can communicate that weay .. if I am not around, they will send me a whatsapp and I will say do this or that.<br>I spend the whole day with them, .. and then I give them a lift home<br><br>Since the strike they have not been the same .. some they understand, some they are aggressive … so I said if you need something, or clarity, sit as CHW, write a memo /list and let your head CHW delivery it .. according to the hierarchy – this really helps, otherwise we have too many complaining and nothing will be sorted out<br>e.g. bags – why only 10 bags; so they wrote it down, I took it to my PN, and the facility manager who was able to sort it out; we discussed how they are going to use the equipment<br>*JG – example of sorting things out*<br><br>It goes back to the in-service training … when I am compiling the stats I am able to see this one is struggling .. when I do supervision I can see this one didn't do it right … I keep quiet in the house,, but immediately we step outside .. as we are walking I do spot training<br><br>Both FM and EN01 had stories of truancy (going home and coming back to clock out);<br>EN02 uses her car to drive them back to the facility if they need to clock out, or allows them to sign out tomorrow if they are working late<br>**EN 02** Sometimes they come back early because of the distance.. because it can be an hours walk … you can waste 2 hours a day walking backwards and forwards<br>**FDG-SM** do you work overtime? If we do household registration, and we don't find anybody who can has the information, we go back in the afternoon or the weekend .. .. Sometimes we deliver medication in the afternoon.. sometimes we are do vital signs at 7.45 |

| | |
|---|---|
| | **FDG-AS:** if some people are very sick we monitor them over the weekend<br>Our team leader is supportive .. if the patient wants to know something .. I ask her |
| Supervision of ENs | **FM:** do induction and appraisals of EN; provide training – HIV (going on Monday), TB (one has gone the other will go in 2017), EVP<br>**EN01:** if sister X is not around she goes to the FM and she provides a solution<br>They help out between 7.30 and 12.00 at the clinic when the clinic is full. When the clinic needs them they pick two CHW to stay and help the EN .. but they are free to say no<br>**EN02:** The facility manager makes sure we attend regular training to uplift ourselves She organizes workshops with CHW to train them on what she has learnt<br>Now I can lead campaigns – cos I understand everything about the campaign – so you see there really is development here..<br>My job is with WBOTs. 70% in the field, 30% in WBOT admin .. if I am not busy I help with vital signs stations.. it is not part of my job but I do it anyway .. I refer my patients and the nurses see them .. So there is teamwork .. The support systemis great so I never felt pressure to leave my wBOT duties<br>**FDG-SM**<br>"And sometimes when you go with the OTL, the patients are feeling uncomfortable because she shouts at them. And we are unable to tell her that what you are doing is wrong, you are not supposed to talk like that with patients. Now the patients are telling us that we are not welcomed in their houses if we come with the OTL."<br>*JG- not clear which OTL is this. .. although know it is ward X* |
| Level of knowledge of CHWs | **FM:** Depends on education of the individual - some will do as you ask, others you need to dig deeper ..<br>**PN**: they are done some of the training (phase 1 and 2)<br>Are they able to implement it ? some are .. others are dragging, but they are trying p7<br>**FDG-SM – what cases aren't you able to deal with?**<br>Sick patient with no food >refer<br>Elderly patients who needs to be in old age home |
| Travel (time, mode, particular features/challenges) | |
| Resources Available | |
| Resources Needed | Have to buy batteries, transport, airtime (although do call-backs), forms, uniform out of own money (although towards the end, the CHW were wearing pink shirts)<br>Don't have office space, or masks for working with TB patients<br>**PN:** forms .. yes, one day a teacher was here from the primary school.. and I asked him to make copies<br>*No photocopier*<br>They need a permanent structure so that they can do maternity<br>We are using our own transport - that is what is irritating<br>**EN02:** Shortage of bags<br>**EN02:** I ma suing my own petrol, my own cellphone, chw use their own airtime.. sometimes they even call patients.<br>We need a place to meet as CHW /OTLS; patient files are stored in PN's room<br>We need our own social worker<br>**FDG_SM – description of the bag**<br>We are meant to screen everybody over 25 years for diabetes.. but we don't because the strips are not enough<br>The machine is already broken .. it is not strong<br>We don't have an office, airtime, batteries (we have to buy out of our money)<br>**FDG-SA:** we were given 10 bags for 17 of us; my machine isn't working.. I reported it .. it is embarrassing when it doesn't work; the strips are finished; we report to the sister who says that the patient must come to the clinic<br>You use your own airtime to call an ambulance;<br>If you are tracing, if you don't find that person at the address, you call them using your own airtime .. otherwise how are going to find them them<br>What makes us not to achieve more is that when we come to the clinic and ask for some more equipment, we are told that that they don't have' |
| Community relationships | **FM:** Initially not accepted, were chased away .. now they are accepted. ..have built rapport<br>community unhappy when CHW on strike (they were saying nurses were on strike p6 |

For peer review only

| | |
|---|---|
| | can do dressings which patients appreciate<br>**PN**<br>• The relationship between the community and the CHW .. 'one day we were out walking with Dr Y and Dr X.. and somebody asked the CHW how come you are walking with so many men' .. she answered they are doctors we are working together' Dr X asked theperson .. how do you know my nurse? .. she brings me medication every month P6 .. Dr X was very excited that they were well known in the community p6<br>• That is why we allocate houses to them in their area.. because then people can call them over the weekend . and then they give us a report and say .. a patient was giving birth .. and so we called an ambulance etc .. it is better if the CHW is in the area<br>• We don't have a clinic committee .. but we have a very good relationship with the ward councilor (ward X?) (EN02 as well – if there is somebody who is sick he calls me, so we work toghether)<br>• I want to start a support group for young people with HIV<br>**EN02**<br>The community is aware of us .. they are coming to us. Even the referral forms its good<br>People are taking treatment now. I think there is lesser tracing on ARV patients, most defaulters you will find are TB. Our stats are going up<br>**Reason for success**<br>Employing us as staff nurses .. cos if the CHW see you as supporting them, trying to understand where they are coming from, especially with this employer thing is mixed up … That is why they refused to go on strike today<br>**EN02**<br>When they were on strike for 3 months .. the patients were complaining .. where have you been, we have been waiting for you .. they decided not to go to the clinic but wait until the sisters were back .. so they really rely on them<br>**FGD-SM**<br>We get the list of defaulters .. but now there are few because of our work .. people have now accepted their illness .. people do appreciate us.. just come and disclose to us .. and later that person will come and thank you for the work you have done p5<br><br>Iyhoo, I'm so proud about my patients, they get better. Others I found them bedridden but because of us they have recovered. Even if they don't give me any material thing, but when I pass them on the street and she/he tells others that she/he is walking because of me, I feel satisfied because at least I have made other people better." P15<br><br>"In ward 8 we don't have any conflict with the community because the ward councilor had introduced us during the public meeting. They know us and they trust us in a way that when they need help even if it is during the night, they wake us. People got that belief that when the CHWs call the ambulance, the ambulance arrives quickly. So we are don't mind because we know the community rely on us."<br><br>We already said, they are able to come to us. Like no the nurse at the clinic is unapproachable, she talks to the patients the way she likes and the patient keep quiet but when they see us, they tell us what happened with the nurse at the clinic. So it is easy for them to talk to us than to the nurses. Other patients said if the nurse comes to their houses and speaks the way she speaks when she is at the clinic, we are going to hit her. Then we try to calm them down." P15<br><br>If my neighbour is sick I ask my partner to attend to her.. cos I don't want the neighbour to say that I talk about her ..<br><br>"The community things we are their helper, they love us. In everything that we do, they stand with us." P25<br><br>"To honest the patients trust us and they appreciate us. I have a patient who is HIV and she doesn't want to come to the clinic and I don't want my patient to default. So I promised her that I will assist. Then I came to the clinic and talk to the sister. I said sister my patient is working at the farms so please pack medication for her, here is her clinic card. I'll ask her to come next month. The sister scolded me because the patient did not come to the clinic on her last appointment. So I begged the sister and I promised the sister that I will bring the patient myself. Then it was sorted, the patient came to the clinic with me and she got her medication." P18 |
| Relationships with other NGOs, | **EN02**<br>We work with policing forum, ward counsellor, we have their numbers .. we do campaigns together .. community and health campaigns (e.g.creches) |

For peer review only

| | |
|---|---|
| networking, knowledge of context (draw on what exists & links clients) | Household registration for municipality .. although you ask these questions about income.. they think you are bringing food .. so they make you feel like you are making empty promises)<br>NGO A – I have referred too many people there .. but there results are not good.. if people don't have an ID they come back with nothing. We need our own social worker |
| Overall impression<br>- Incl do CHWs feel valued?<br>- Other notes | During the strike some of the CHW tried to work .. but the EN and the FM told them not to<br>**EN02:** strike started again today but CHW refuse to be part of strike (strike is for better employment issues, high bank card deductions (20R), lack of communication from smart purse, uniform); eventually they had to accepted it .. so not joining strike today<br><br>**Can the programme be led by staff nurses ?**<br>**EN02** I am saying this because with staff nurses in the community … I can deal with almost 90% of the problems .. but as a staff nurse I am not allowed to give out treatment.. if I pre-pack medication .. it has to be checked by the professional nurse .. also I can't consult |
| Interesting points | • FM is involved, and knows how the WBOT programme runs, clinic was set up and the same time as the WBOT<br>• EN02 know there is a reciprocal relationship between WBOT and clinic and need to maintain that ..(helps when clinic is busy and they see her patients)<br>• If clinic is not welcoming to patients .. difficulty in persuading patients to go to the clinic .. So success of WBOT is dependent on quality of care in clinics<br>• In EN only model – a PN has to be available to dispense the medication (or check it?)<br>• It seems that EN02 who goes out into the community has a better relationship with the CHW, the trust each other more .. a lot of the problems are about lack of trust<br>• EN 01 is a lot more passive and prefers to work in the clinic;<br>• PN sees patients but doesn't seem to meet with CHW when they return .. EN02 knows households and really works with CHW |

For peer review only

Consolidated criteria for reporting qualitative studies (COREQ): 32-item checklist

| Item | | **Guide questions/description** | |
|---|---|---|---|
| **Domain 1: Research team and reflexivity** | | | |
| **Personal Characteristics** | | | |
| 1. | Interviewer/facilitator | Which author/s conducted the interview or focus group? | **Field workers trained by researchers collected data** |
| 2. | Credentials | What were the researcher's credentials? *E.g. PhD, MD* | **PhD, MD, MS and MA** |
| 3. | Occupation | What was their occupation at the time of the study? | **Professors, researchers, postdocs, and PhD fellows** |
| 4. | Gender | Was the researcher male or female? | **6 females + 1male** |
| 5. | Experience and training | What experience or training did the researcher have? | **Health policy, qualitative, health systems research, social work** |
| Relationship with participants | | | |
| 6. | Relationship established | Was a relationship established prior to study commencement? | **No** |
| 7. | Participant knowledge of the interviewer | What did the participants know about the researcher? e.g. *personal goals, reasons for doing the research* | **Researchers and fieldworkers explained to participants the reasons for doing the research** |
| 8. | Interviewer characteristics | What characteristics were reported about the interviewer/facilitator? e.g. *Bias, assumptions, reasons and interests in the research topic* | **Researchers explained the aims and objectives of the study to the participants.** |
| **Domain 2: study design** | | | |
| Theoretical framework | | | |
| 9. | Methodological orientation and Theory | What methodological orientation was stated to underpin the study? *e.g. grounded theory, discourse analysis, ethnography, phenomenology, content analysis* | **Case study** |
| Participant selection | | | |
| 10. | Sampling | How were participants selected? *e.g. purposive, convenience, consecutive, snowball* | **Purposive** |
| 11. | Method of approach | How were participants approached? e.g. *face-to-face, telephone, mail, email* | **Either telephonically, or face-to-face.** |
| 12. | Sample size | How many participants were in the study? | **201 individuals were being observed or/and interviewed.** |
| 13. | Non-participation | How many people refused to participate or dropped out? Reasons? | **Only one senior nurse supervisor. However, she didn't really refuse, but only rescheduled a number of times and we eventually took it as a reluctance to participate.** |
| Setting | | | |
| 14. | Setting of data collection | Where was the data collected? e.g. *home, clinic, workplace* | **Home for household members; workplace (incl. community and facility) for CHWs and supervisors** |

For peer review only

| | | | |
|---|---|---|---|
| 15. | Presence of non-participants | Was anyone else present besides the participants and researchers? | **Yes, during observations, where there were community members and clinic staff.**<br>**No, during focus group & interview.** |
| 16. | Description of sample | What are the important characteristics of the sample? *e.g. demographic data, date* | **Characteristics of the sample related to research are provided in Results, on pages 15-16, lines 204-224.** |
| Data collection | | | |
| 17. | Interview guide | Were questions, prompts, guides provided by the authors? Was it pilot tested? | **Yes.**<br>**Pilot tested between researchers and fieldworkers (facilitators).** |
| 18. | Repeat interviews | Were repeat interviews carried out? If yes, how many? | **Follow-up focus group discussions at 4 of the clinic-based CHWs** |
| 19. | Audio/visual recording | Did the research use audio or visual recording to collect the data? | **Interviews and focus discussions were audio recorded for transcription, as they were done in local language.** |
| 20. | Field notes | Were field notes made during and/or after the interview or focus group? | **Yes, notes were made for every piece of data, both during data collection and afterwards.** |
| 21. | Duration | What was the duration of the interviews or focus group? | **5 months** |
| 22. | Data saturation | Was data saturation discussed? | **Yes** |
| 23. | Transcripts returned | Were transcripts returned to participants for comment and/or correction? | **No** |
| **Domain 3: analysis and findings** | | | |
| Data analysis | | | |
| 24. | Number of data coders | How many data coders coded the data? | **All 7 authors** |
| 25. | Description of the coding tree | Did authors provide a description of the coding tree? | **No** |
| 26. | Derivation of themes | Were themes identified in advance or derived from the data? | **Both** |
| 27. | Software | What software, if applicable, was used to manage the data? | **Done manually** |
| 28. | Participant checking | Did participants provide feedback on the findings? | **Feedback has been given to provincial, district and facility management, as well as CHW supervisors and CHWs in the intervention sites in various forms, including facility level workshops. Participants generally agreed with the findings.** |
| Reporting | | | |
| 29. | Quotations presented | Were participant quotations presented to illustrate the themes / findings? Was each quotation identified? e.g. *participant number* | **Yes** |
| 30. | Data and | Was there consistency between the data | **Yes** |

For peer review only

| | | | |
|---|---|---|---|
| | findings consistent | presented and the findings? | |
| 31. | Clarity of major themes | Were major themes clearly presented in the findings? | **Yes** |
| 32. | Clarity of minor themes | Is there a description of diverse cases or discussion of minor themes? | **Yes** |

For peer review only

BMJ Open

# Integrating community health workers into the formal health system to improve performance: a qualitative study on the role of on-site supervision in the South African programme

| | |
|---|---|
| Journal: | *BMJ Open* |
| Manuscript ID | bmjopen-2018-022186.R3 |
| Article Type: | Research |
| Date Submitted by the Author: | 17-Dec-2018 |
| Complete List of Authors: | Tseng, Yu-hwei; University of the Witwatersrand School of Public Health, Centre for Health Policy<br>Griffiths, Frances; University of Warwick Warwick Medical School<br>de Kadt, Julia; University of the Witwatersrand School of Public Health, Centre for Health Policy Johannesburg, Gauteng, ZA<br>Nxumalo, Nonhlanhla; University of the Witwatersrand School of Public Health, Centre for Health Policy<br>Rwafa, Teurai; University of the Witwatersrand School of Public Health, Centre for Health Policy<br>Malatji, Hlologelo; University of the Witwatersrand School of Public Health, Centre for Health Policy<br>Goudge, Jane; University of the Witwatersrand School of Public Health, Centre for Health Policy |
| <b>Primary Subject Heading</b>: | Health services research |
| Secondary Subject Heading: | Qualitative research, Public health, Health policy |
| Keywords: | Human resource management < HEALTH SERVICES ADMINISTRATION & MANAGEMENT, Organisation of health services < HEALTH SERVICES ADMINISTRATION & MANAGEMENT, QUALITATIVE RESEARCH, PUBLIC HEALTH, Health policy < HEALTH SERVICES ADMINISTRATION & MANAGEMENT |
| | |

SCHOLARONE™
Manuscripts

# Integrating community health workers into the formal health system to improve performance: a qualitative study on the role of on-site supervision in the South African programme

Yu-hwei Tseng,[1] Frances E Griffiths,[1,2] Julia De Kadt,[1] Nonhlanhla Nxumalo,[1] Teurai Rwafa,[1] Hlologelo Malatji,[1] Jane Goudge[1],

**Affiliations**

[1] Centre for Health Policy, School of Public Health, University of the Witwatersrand, Johannesburg, South Africa;

[2]Division of Health Sciences, Warwick Medical School, University of Warwick, Coventry, United Kingdom

**Address correspondence to:**

Jane Goudge

Centre for Health Policy (CHP)

School of Public Health, Faculty of Health Sciences, University of the Witwatersrand

Private Bag X3 Wits 2050, Johannesburg, South Africa

Tel: +27 11 717 3425; Email: jane.goudge@gmail.com

**Keywords:** community health worker, supervision, South Africa, health system integration, health post

**Abstract word count: 270**

**Text word count: 7,104**

**Table: 5**

**Box: 1**

**Number of references: 30**



For peer review only - http://bmjopen.bmj.com/site/about/guidelines.xhtml

## ABSTRACT

### Objectives

To explore the role of on-site supervision in community health worker (CHW) programmes and CHW integration into the health system. We compared the functioning of CHW teams reporting to a clinic-based nurse, with teams supervised by a community-based nurse. We also consider whether a junior nurse can provide adequate supervision, given the shortage of senior nurses.

### Design

A case study approach to study six CHW teams with different configurations of supervision and location. We used a range of qualitative methods: observation of CHW and their supervisors (126 days), focus group discussions (12) and interviews (117).

### Setting

South Africa where a national CHW programme is being implemented with on-site supervision.

### Participants

CHWs, their supervisors, clinic managers and staff, district managers, key informants from the community, and CHW clients.

### Results

Effective supervisors supported CHWs through household visits, on-the-job training, debriefing, reviewing CHWs' daily logs, and assistance with compiling reports. CHWs led by senior nurses were motivated and performed a greater range of tasks; junior nurses in these teams could better fulfil their role. Clinic-based teams with senior supervisors were better integrated and more able to ensure continuity of care. In contrast, teams with only junior supervisors, or based in the community, had less engagement with clinic staff, and were less able to ensure necessary care for patients, resulting in lower levels of trust from clients.

### Conclusion

Senior supervisors raised CHW skills, and successfully negotiated a place for CHWs in the health system. Collaboration with clinic staff reduced CHWs' marginalisation, and increased motivation. Despite being clinic-based, teams without senior supervisors had lower skill levels and were less integrated into the health system.




For peer review only - http://bmjopen.bmj.com/site/about/guidelines.xhtml

**Strengths and limitations of this study**

- To the best of our knowledge, this is the first study to examine the role of different levels of supervision and location of the CHW team on the team's motivation, performance and integration into the health system.
- The case study approach allowed us to purposively select teams based on their supervision/ location configurations, and to compare and contrast their functioning to understand what worked, and what did not.
- We employed multiple qualitative methods, including participant observation, interview and focus group discussion, to collect a substantial amount of data, enabling triangulation across data sources and sites.
- Limitations to the study include the observer effect (Hawthorne effect) during data collection, subjective rather than objective evaluations of CHW performance, and data collection limited to a single health district.

For peer review only



For peer review only - http://bmjopen.bmj.com/site/about/guidelines.xhtml

## INTRODUCTION

Community health workers (CHWs) play an important role in improving health care coverage and access in low and middle-income countries (LMICs).[1-3] However, optimizing the contribution of CHWs continues to be a global challenge. CHWs, with no professional qualification, perform primary health care (PHC) related functions in the community.[1, 4] They work on the periphery of the health system and often in under-served communities where needs are immense and multifaceted. For this reason, CHWs require frequent and supportive supervision that supplements their training and connects them with the health system.[3, 5, 6] Recent reviews suggest that adequate supervision, and integration into the health system, are essential components to programme effectiveness.[5, 7-9]

Effective integration of CHW programmes into the health system requires government financing, national level planning, training and CHW scope of work, supervision, referral networks, and supply chains to be connected to and incorporated into similar processes provided for other cadres.[9] Without integration, there is unlikely to be a smooth patient pathway from CHW to clinic, limiting the effectiveness of CHWs. There is a growing literature on supervision, with some evidence that supportive supervision improves motivation and performance.[10-14] However, much of this evidence is from



For peer review only - http://bmjopen.bmj.com/site/about/guidelines.xhtml

NGO programmes (which are less integrated into the health system), or from programmes in which the CHWs' work is focused on a specific disease (e.g. HIV) or population (e.g. maternal). With the goal of universal access to comprehensive, integrated care, it is important to understand the type and level of supervision that will enable CHWs to provide the full range of required tasks, as well as facilitate integration into the health system.[12-15]

Given the double burden of non-communicable and infectious diseases in LMICs, governments such as South Africa's are seeking to shift CHW initiatives from focusing on a single disease or population group, to a more comprehensive approach.[16] South Africa is implementing a national CHW programme (WBOTs; ward-based outreach teams) as part of efforts to strengthen primary health care.[17] The intention is to provide full-time on-site supervisors whose responsibility is to manage, train, mentor, and monitor CHWs and to facilitate links with the health system and the community. However, given the shortage of health care workers, as in other LMIC settings, there is a limited number of nurses available to supervise CHWs. In this study, we ask whether CHW teams should be linked to a clinic, and report to a clinic-based nurse who may facilitate referrals, access to supplies, and CHWs' integration into the health system. Or should CHWs be based in the community in which they are working, with a dedicated

For peer review only



For peer review only - http://bmjopen.bmj.com/site/about/guidelines.xhtml

nurse who doesn't have the additional demands of clinic duties? We also ask whether a junior (enrolled) nurse is able to provide adequate supervision, given the shortage of senior (professional) nurses. By asking these questions, we examine supervision practices, the team's integration into the health system, and their effect on CHW motivation and performance.

## METHODS

In the initial observation phase (Sept 2016- Feb 2017) of a 3-year intervention study in Sedibeng Health District, Gauteng Province we used a case study approach to examine the operation of CHW teams with different configurations of supervisors and locations: (1) clinic-based teams supervised by a senior nurse (professional nurse; PN) and a junior nurse (enrolled nurse; EN); (2) community (health post)-based teams supervised by a PN and an EN; and, (3) clinic-based teams supervised by an EN only. We studied six teams (two of each of the three configurations); each team and their supervisors being a single case study.

### Study site

In some South African provinces, CHWs, previously employed by non-governmental organizations (NGOs), have been absorbed into a government WBOT programme in an



For peer review only - http://bmjopen.bmj.com/site/about/guidelines.xhtml

attempt to shift from a patchwork of condition-specific services to a comprehensive programme with a national scope.[15, 18] The CHWs' role is predominately health promotion, prevention, and screening. Standardized CHW training covers identification of the need for ante-natal and post-natal care, monitoring immunisation of under 5s, adherence among patients with chronic diseases, screening for malnutrition, TB, gender based violence, and making referrals to health, social and other services. WBOT teams are meant to be comprised of a professional nurse, six or more CHWs, one health promoter and one environmental officer,[17] although realities on the ground often do not allow this.

We selected Sedibeng Health District as our study site due to the variation in supervision structures and location of its CHW teams. The district is relatively affluent by South African standards, although over 20% of its residents fall below the food poverty line. Outside the urban areas, disadvantaged communities with inadequate shelter, food insecurity and high disease burdens have limited access to services such as clinics, transport, water and electricity.

Sedibeng District has established 16 health posts to provide community-orientated care, different from the disease focused care provided by 28 primary health care clinics.[19]



For peer review only - http://bmjopen.bmj.com/site/about/guidelines.xhtml

A health post consists of one or two temporary structures (providing 3-6 rooms), often operating without electricity and with irregular water supply. It is managed by one or two PNs, who obtain medication and other resources via a 'mother' clinic. The nurses supervise the CHW team, and provide basic services such as chronic medication, immunisation and treating minor ailments. Some health posts are placed at some distance from a clinic, in order to improve access to basic services.

At the time of the study there were 39 CHW teams (each with between 6-20 CHWs) in 37 of the district's 72 wards (the smallest geopolitical area). Sixteen of the teams were based at health posts and the remaining 23 were clinic-based. In addition to the services outlined in the national training programme (see above), the CHWs delivered medication to elderly or disabled chronically ill patients.

**Selection of CHW teams for study**

The research team consulted with district officials, categorizing the CHW teams into three types. We selected two teams of each type with the requirement that each pair of teams needed to be as similar as possible (important characteristics included urban or rural location, the size of the teams, and the type of community they served). (We needed to pool the data from the two teams to generate a sufficient sample for the



For peer review only - http://bmjopen.bmj.com/site/about/guidelines.xhtml

analysis of the coverage data, reported elsewhere). The key characteristics and description of catchment areas of each team are shown in Table 1. The two health post teams were closest to the community, while the rural teams had the largest geographical area to cover.

**Table 1. Supervision configurations of CHW teams and their catchment areas**

| Type | Supervisor | Based in | Site Code | Description of urban/rural location and type of community served | Max. distance CHW base to furthest household |
|---|---|---|---|---|---|
| 1 | Professional & Enrolled nurse | Clinic | 1A | Township[1] with relatively well-off households and apartheid-era housing | 2 km |
| | | | 1B | Township with RDP[2] housing and informal settlement[3] | 3 km |
| 2 | Professional & Enrolled nurse | Health post | 2A | Township | 1 km |
| | | | 2B | Large informal settlement, with many migrants | 1 km |
| 3 | Enrolled nurse only | Clinic | 3A | Rural, informal settlement, also with RDP housing and farm plots | 6 km |
| | | | 3B | Rural, township and farm plots | 25 km |

[1] Township: densely populated urban area built on the peripheries of towns and cities; [2]RDP housing: low-cost government built housing; [3] informal settlements: where impoverished population build shacks on vacant land.

## Data collection

Eight fieldworkers received two weeks of training, covering research methods and ethics, study tools, extensive role-play and observation practice, as well as an overview of training that CHWs receive. Between Sept 2016 and Feb 2017, the field team collected interview and observation data on each team's activities, resources, engagement with the clinic and community, successes and challenges, as well as client and key informant views of the performance of the teams. (Further details of CHW



For peer review only - http://bmjopen.bmj.com/site/about/guidelines.xhtml

performance using more objective indicators, such as coverage and the quality of care, will be reported elsewhere.) Data collection method and types of data are summarised in Table 2

**Table 2: Data collection method, participants and data collected**

| Method | Participants | Total number in 6 sites | Data collected |
|---|---|---|---|
| Observation | CHWs and supervisors while conducting their daily work | 126 days of observation | Descriptions of activities, interactions and clients; |
| FGD[1] | CHW teams | 12 FGDs (76 participants) | FGD: descriptions of activities and weekly & daily routines; resources available and needed; support from supervisors, peers, clinic and community; employment conditions; challenges of the programme<br><br>Self-administered questionnaire: age, years of training and service, |
| Interviews | Supervisors and facility managers | 43 key informant interviews | Background, training responsibilities, weekly & daily pattern of CHWs; resources; successes and challenges |
| | Community representatives | | Perceptions of the programme; acceptability to the community needs; |
| | Follow-up interviews with CHWs' clients who were referred to the clinic during observations of household visits | 74 household interviews | Client's perception of the service, and events subsequent to the referral |

[1] FGD: focus group discussion.

Fieldworkers observed CHWs and their supervisors throughout the workday, and wrote detailed daily field notes guided by a template. After each day spent in the field, the fieldworkers spent a day in the office typing up field notes in full. Each CHW or supervisor was observed for 3-5 consecutive days (interrupted by the office days) to allow for reduction in the Hawthorne effect. Participants were randomly selected, although changes were sometimes required due to CHWs taking leave or being absent. Participants responded well to observation, and relaxed substantially during the course

For peer review only

of the first day of observation, although there is some evidence the CHWs conducted more household visits early on in the observations.

All available CHWs participated in the FGDs, and all supervisors and facility managers (except one) were interviewed. Community representatives were purposively sampled. Key informant interviews were recorded and transcribed. Finally, 74 household members, who received referral advice from CHWs during observed home visits, were interviewed a month later, to understand their experience of the CHW service, and whether referral advice was acted upon. Interviews were conducted in participant homes, in the participant's choice of language(s), and recorded with a digital recorder. Field workers used the recording to draft a summary of the interview in English.

**Data analysis**

Taking a case study approach, we drew data from the various sources to develop an explanatory description of each team, and then drew comparisons across teams. This process involved several steps. Firstly, we extracted data from each interview or day's observation into a template, either by summarising descriptions of events, or extracting raw data such as useful quotations. This increased our familiarity with the data and allowed us to reduce its volume significantly. Multiple team members extracted data



For peer review only - http://bmjopen.bmj.com/site/about/guidelines.xhtml

from the same sources, compared extracted data and modified extraction strategies until we were confident about inter-extractor reliability.

Secondly, the authors presented a brief summary of each site in a one-day workshop. Through this collective process we identified themes that revealed the similarities and differences across the sites, such as weekly and daily pattern of activities, resources available, record keeping, managing patient referrals to the clinic, engagement with clinic staff, relationship between CHWs and supervisors, relationship with patients, local NGOs, and key community stakeholders. We then generated a template into which we collated site-specific data under these themes, producing a summary for each site (see example in appendix).

In preparing the manuscript, we continued to revisit the raw and summarised data to confirm descriptions of the context, enrich the content, provide clarity, and check emergent ideas. The multiple data sources, as well as events documented by multiple field workers, allowed triangulation that increased the validity of our findings.

**Ethics**



For peer review only - http://bmjopen.bmj.com/site/about/guidelines.xhtml

The study protocol and instruments were approved by the University of the Witwatersrand's Human Research Ethics Committee (Medical) (M160354), the Gauteng Provincial Health Research Committee in South Africa, and the Biomedical & Scientific Research Ethics Committee (BSREC) at Warwick University (REGO-2016-1825), United Kingdom. All participants gave informed consent. When accompanying CHWs into the community, fieldworkers obtained verbal consent from household members, prior to entering any household. There were few refusals.

**Patient and Public Involvement**

Patients were not involved other than as respondents. Feedback has been given to provincial, district and facility management, as well as CHW supervisors and CHWs in the intervention sites in various forms, including facility level workshops.

For peer review only



For peer review only - http://bmjopen.bmj.com/site/about/guidelines.xhtml

# RESULTS

## CHW conditions of work across districts

The lack of integration led to work conditions that were demotivating for CHWs. They were not formally employed by the government, but were an outsourced workforce, managed by a private payroll company, and paid a minimal stipend for 6 hours work a day. They struggled to contact the private payroll company responsible for monitoring attendance and paying stipends. An electronic system requiring CHWs to clock in/out at the clinic limited their ability to reach outlying areas as they needed to travel between the clinic and these areas on foot. Two months prior to the start of our fieldwork CHWs had been on strike over their conditions of employment. Moreover, opportunities for career development into nursing were limited, particularly for those without a high school diploma.

CHW work was hampered by insufficient provision of logistical support due to the lack of integration. They often worked without necessary resources, including equipment, stationery, uniform, name badges, or funds for transport or communication. For example, a team of 20 CHWs shared 1 or 2 blood pressure machines borrowed from the facility. When equipment, such as a glucometer, was provided, the associated consumables such as glucose strips were often not replenished. Notebooks used by

For peer review only



For peer review only - http://bmjopen.bmj.com/site/about/guidelines.xhtml

CHWs to record daily activities were purchased out of their own stipend. The general lack of resources compromised CHWs' work and generated resentment.

While teams based in health posts had some dedicated space, clinic-based teams did not have sufficient space to meet, complete paper work or store their files; often files were kept at home. Supervisors did try to address these deficiencies, and some of the endeavours appeared to be morale boosting. CHWs reported that when one supervisor negotiated with the facility manager to use a meeting room for CHW meetings, it immediately increased their job satisfaction.

*"Before she (PN) came, we were like orphans, we had no space inside the clinic. We were doing everything outside. She came with a lot of changes around here…PN had played an important role in our lives…I even enjoy my work now." (CHW-FGD, 1A)*

**The supervisors**

The supervisors in our study sites were either senior (ie. professional) or junior (ie. enrolled) nurses. A professional nurse (PN), with a 4-year degree in nursing, is able to diagnose patients, prescribe treatment and dispense medication. Importantly, the PN supervisors in the study site were trained in primary health care and community nursing,



For peer review only - http://bmjopen.bmj.com/site/about/guidelines.xhtml

and had attended various other courses on TB, HIV, diabetes and hypertension, integrated management of childhood illnesses (IMCI), nursing management and leadership. Many were rehired retirees[1], who had more than 30 years of experience as a nurse before they joined the programme (Table 3). Enrolled nurses (EN) have completed a 2 year course and can provide nursing care under supervision of a PN. All ENs except one (site 3B) received 1-week induction training on the WBOT programme before joining. Some of the EN supervisors in the site had also attended courses on early childhood development (ECD), TB, prevention of mother to child transmission (PMCT), and the expanded immunisation programme (EPI). Most of the ENs were younger and less experienced in community work than the CHWs they supervised. The PN supervisors reported to the facility manager, as did the EN supervisors, if there was no PN supervisor in the team.

**Table 3. Supervision of CHW – who, what and when**

| SUPERVISION AND LOCATION OF TEAMS | PN / EN clinic-based | | PN / EN health post-based | | EN clinic-based | |
|---|---|---|---|---|---|---|
| | 1A | 1B | 2A | 2B | 3A | 3B |
| **Who supervises and is supervised** | | | | | | |
| **PN supervisor** | | | | | | |
| Age (years) | 65 | 72 | 66 | 59 | n.a. | n.a. |
| Years as nurse | 36 | >30 | 38 | 39 | n.a. | n.a. |
| Years in programme | 4 | 4 | 4 | 4 | n.a. | n.a. |
| **EN supervisor** | | | | | | |
| Number of JN in team | 2 | 2 | 1 | 1 | 1 | 1 |
| Mean age (years) | 43 | 29 | 28 | 25 | 36 | 31 |
| Mean years as nurse | 14 | 2.5 | 0.5 | 3 | 5 | 2 |
| Years in programme | 0.6 | 0.6 | 0.6 | 0.6 | 0.3 | 0.3 |
| **CHW** (supervisee) | | | | | | |
| Number of CHW in team | 16 | 17 | 9 | 12 | 14 | 20 |
| Mean age in years (range) | 38 (26-53) | 39 (25-47) | 34 (27-45) | 37 (26-51) | 42 (23-58) | 33 (23-54) |
| Mean years (range) as CHW | 7 (4-12) | 7 (3-16) | 7 (6-9) | 6 (3-12) | 10 (3-9) | 6 (5-17) |
| **Formal training of CHWs** | | | | | | |



For peer review only - http://bmjopen.bmj.com/site/about/guidelines.xhtml

| SUPERVISION AND LOCATION OF TEAMS | PN / EN clinic-based | | PN / EN health post-based | | EN clinic-based | |
|---|---|---|---|---|---|---|
| | 1A | 1B | 2A | 2B | 3A | 3B |
| % CHW received phase 1 training | 91% | 93% | 100% | 90% | 5.6% | 16.7% |
| % CHW received phase 2 training | 91% | 21% | 100% | 46% | 0% | 0% |
| **<u>Supervision activities</u>** | | | | | | |
| **Supervised home visits (day/week)** | | | | | | |
| By professional nurse | n.o. | n.o. | 1 | Occasional | n.a. | n.a. |
| By enrolled nurse | 3 | 4-5 | 4-5 | s.s. | 4 | On request |
| **On-the-job training** | Yes | Yes | Yes | Yes | n.o. | s.s. |
| **Regular debriefing** | Daily | n.o. | Daily | Weekly | s.s. | s.s. |
| **Examining daily logs and registers** | Yes | Yes | n.o. | Yes | n.o. | n.o. |
| **Assisting in reporting** | Yes | Yes | n.o. | Yes | n.o. | n.o. |
| Frequency of preparing reports | Weekly | Monthly | Monthly | Weekly | Weekly | Monthly |
| **Resolving administrative matters** | Yes | Yes | Yes | n.o. | n.o. | n.o. |

Notes: n.a.: not applicable; n.o.: not observed & not indicated in other information sources; s.s.: the activity was mentioned by a single source other than CHWs, but was not indicated in other data sources.

## Supervisor activities

*<u>Formal and informal (on-the-job) training</u>*

In four of the six sites, 90~100% of the CHWs had completed the first phase of the national training programme, but only 3 sites (all with PN supervisors) had a significant number of CHWs who had passed the second phase of training (Table 3). Both phases require a written test, followed by a practical assessment conducted by the supervisor during household visits; success in this assessment requires a competent supervisor, and comprehensive and on-going training. One PN placed considerable emphasis on training. *"When PN came, she checked who had done what course and organized training for those who needed it."* (*CHW-FGD, 1A*). This PN also used daily morning meetings as training sessions, *"our PN is teaching us ... every day in the morning."* *(CHW-FGD, 1A)*. Given the volume of information that CHWs were required to retain, these regular sessions were important. This PN involved the two ENs in the training:

For peer review only

*"Sister teaches us like nobody's business" (EN, 1A)*, enabling the ENs to take up the supervisory role.

In contrast, in the EN-only sites, supervisors were unable to arrange formal training for the CHWs, in part due to their rural location at some distance from the district training centre, and because the ENs were not sufficiently skilled to carry out the practical assessment. With little experience themselves, they were also unable to provide on-the-job training.

*Supervised home visits*

ENs routinely accompanied different pairs of CHWs on home visits several days a week. Some ENs demonstrated sensitivity in correcting CHWs' practice. *"When I can see this one didn't do it right, I keep quiet in the house, but immediately after we step outside as we are walking I do on-the-spot training" (EN, 1B)*. Other ENs working without a PN supervisor, seemed uninvolved during the home visits. Despite one EN spending 4 days a week out in the community, the CHWs did not acknowledge her as a supervisor, *"When we get there she's doing the same job that I normally do when I am alone." (CHW-FGD, 3A)* Consequently, her CHWs didn't report problems they



For peer review only - http://bmjopen.bmj.com/site/about/guidelines.xhtml

encountered to her, such as suspected TB patients, as they had little trust in her problem solving capability.

*Regular debriefing*

One health post-based PN/EN team (2A) held daily debriefing sessions, at which CHWs described the households visited, problems encountered and actions taken. The sessions strengthened CHWs' knowledge base and problem-solving abilities, and provided a platform for collective supervision, enabling the CHWs to learn from each other's experience.

One team (1A) held debriefings in conjunction with other activities, such as in-service training or medication preparation, which diluted the effects of debriefing. In other teams, feedback was said to happen once a month, but we were unable to validate this, and difficulties with recall of events for a whole month would likely limit the utility of this approach. Those teams that had no place to meet inside the facility tended not to discuss serious matters for reasons of confidentiality. *"Sometimes we stand outside to hold meetings… patients and the security personnel will be listening to what we are discussing." (CHW-FGD, 3A)*



For peer review only - http://bmjopen.bmj.com/site/about/guidelines.xhtml

The CHWs used notebooks to document their daily activities. Through reviewing these notes, experienced supervisors could discuss the visits with the CHWs, ask questions and provide support, as observed in teams 1A, 1B and 2B. In every team there were CHWs that took poor notes, but this was better contained by supervisors willing to spend time reviewing records, and discussing problems with the relevant CHWs. It sent a clear message that documentation of activities was central to the work.

*Assistance in compiling reports*

PNs of two teams (1A & 2B) required CHWs to compile their statistics every Friday, as doing it on a weekly basis (rather than monthly) reduced recall error and thus enhanced quality. One PN verified the weekly report of each CHW against their notebooks, correcting mistakes and making sure that the CHWs understood the meaning of each data category and how notes were translated into figures. This practice strengthened the ability of CHWs to record their work, and trained them to be accountable for what they did. To make sure that CHWs reported faithfully, another supervisor would make unsolicited household visits, *"Sometimes I would just go out, and when they talk about households, I would say, oh that one who lives in that shack? And they would say, 'Sister you went there?' I would say, Yes, it is necessary."(SN, 2B)*

In contrast, EN supervisors without senior support, tended to simply accept whatever



For peer review only - http://bmjopen.bmj.com/site/about/guidelines.xhtml

reports the CHWs handed over, with no discussion or interrogation of the statistics produced.

<u>*Clinic duties*</u>

There was an understanding between district officials and facility staff that clinic-based supervisors were to spend 70% of their time on CHW duties, and assist clinic staff in the remaining 30% of their time, particularly if the clinic was short staffed. District officials regularly restated this 'rule', as they were concerned about supervisors neglecting their CHW duties. We did observe EN supervisors, who didn't have PN supervisors, spending substantial amount of time in the clinic, but this was because they felt unable to contribute to CHW activities. However, one EN with PN supervisor said: *"if I am not busy I help with vital signs stations… it is not part of my job but I do it anyway … I refer my patients and the nurses see them. So there is teamwork. The support system is great so I never felt pressure to leave my WBOT duties."* (EN interviews, *1B*)

*"Remember, (patients) in this family planning (unit of the clinic), are also the children of the mothers and the fathers that we deliver the medication at home. So, we're working hand in hand.…even if they (clinic staff) ask, are you finished*



For peer review only - http://bmjopen.bmj.com/site/about/guidelines.xhtml

*what you were doing? If you don't mind, can you please help me to take a blood pressure on this patient? Then I will do.*" (*EN interview, 1A*)

**The consequences of supervision**

*Motivation, job satisfaction and engagement*

Job satisfaction, professional confidence and motivation were more evident in teams with effective supervision. One CHW commented that since the PN arrived: *"we started to feel that we are now CHWs and human beings." (CHW-FGD, 1A)* Two ENs supervised and mentored by PNs, explained: *"At first it was challenging for me leaving the hospital and working in the community… maybe I'm not a nurse anymore, but now I feel more useful. The community is aware of us … they are coming to us. People are taking treatment now. I think there is lesser tracing on ARV patients, most defaulters you will find are TB. Our stats are going up" (EN, 1B) "I'm a street nurse…but you know what, when I reach the households, this is very enjoyable." (EN, 1A)* When CHWs were on strike over issues with their contract in 2016, some CHWs at the clinic-based PN/EN site (1B) continued working, not wanting to let their patients down or damage their relationship with supervisors and the facility. Their supervisor interpreted their actions: *"they see that you, as their leader, are supporting them and trying to*



For peer review only - http://bmjopen.bmj.com/site/about/guidelines.xhtml

*understand where they come from, especially with this employer thing being mixed up. That is why I am saying, hence, they refused to go on strike today." (EN, 1B)*

However, in the absence of supportive supervision, insufficient training constrained CHWs ability to assist patients, and as a result, demotivated them. Their frustrations over their working conditions were unresolved, and sometimes unacknowledged by their immediate supervisors. Some CHWs decided to work fewer hours a day as a passive protest, in addition to participating in the collectively organised strikes.

<u>*Performance*</u>

CHWs with engaged and skilled supervisors carried out a fuller range of activities, and used their discretion to make decisions. For example: *"A patient had an elevated blood glucose level. However, it was too late for the patient to go to the clinic, so rather than writing a referral letter, she visited the patient again with glucometer after work, by which time the glucose level had dropped by 50%. The next morning, she went for a third time and found that sugar level had returned to normal." (Observation notes, 1A*)
This CHW, from a clinic-based PN/EN team, was able to interpret the glucometer readings, and re-checked the patient to avoid making an unnecessary referral.



For peer review only - http://bmjopen.bmj.com/site/about/guidelines.xhtml

The dedication of some CHWs to their work is evident in the following client interview: *"If you send a message to a CHW, it does not matter what time it is, or what the problem is, they will come and help you. Whatever problem you have, you can talk to them, and you are not worried that they will be talking about your problem around the area. I like the work they do very much." (Patient-BT, 2A)* In the EN-only model where supervision was the weakest and support from facility staff was negligible, CHW activities were minimal, with CHWs often only delivering medication to the elderly. One CHW highlighted how a colleague's poor performance impacted on the collective reputation of the team, *"It's not good if the patient comes here and when one of the clinic staff checks the card finds out the medication delivery is long overdue. It affects all of us in the eyes of the clinic staff and the PN." (CHW-FGD, 1A)*

<u>*Collaboration between CHWs and facility staff*</u>

Located at a distance from the main clinics, health post teams received little supervisory support from clinic staff. Other than supplying medication, there was little managerial oversight by the clinic. *"I am not directly supervising them except for sometimes when they are there. You can only supervise something that you can see. With the CHW team it is just via the phone and then it ends there." (Facility Manager, 2A)* In the health post located in a community without a clinic, the PN spent a considerable amount of time



For peer review only - http://bmjopen.bmj.com/site/about/guidelines.xhtml

seeing patients, and so neglected her CHW supervision responsibilities. Furthermore, the distance between the health post and the clinic hampered efforts to ensure CHWs' patients accessed to care at the clinic.

*"The saddest part is when the patient comes to the clinic and the file is not there, the doctor does not see the patient, and the patient is getting upset with us. Every time when we go to the clinic, we have to look for the files, the clerks do not assist us and we don't even find those files."(Observation notes from conversation with CHW, 2A)*

In clinic-based team, without a senior supervisor to assist, management of files was also a problem:

*" Sometimes you find the admin clerk is in a bad mood, she speaks to us in a bad way in front of the patients, "I am not going to give you the file. You have to go back to the patient to tell the patient to come to the clinic to collect it." You report to the sister, she will tell you that she has been warning [the admin clerk] for several times but she does not listen." (CHW-FGD, 3A)*

CHWs reported that other facility staff expressed a dismissive attitude towards them:



For peer review only - http://bmjopen.bmj.com/site/about/guidelines.xhtml

*"the peer educators, HIV/AIDS counselor we all go together to sign the same contract but they are treated as if they are more educated than us, they call us street maids" (CHW-FGD, 3B)*

When asked why they were not allowed to work inside the facility, the CHWs responded: *"the manager tells us that we are not part of the clinic, so there's nothing she can do for us."* (field notes, 3A) The denial of sharing space exacerbated the CHWs' sense of marginalisation and exclusion.

However, in clinic-based teams, with the support of senior supervisors, and the resulting growth in skills and confidence, CHWs had been able to establish better relationships with clinic staff. In turn, clinic staff, seeing the CHWs' greater motivation and effort, were more likely to treat the CHWs with respect. There were several benefits to this resulting collaboration. Firstly, communication and coordination was better. Working together, the facility manager and PN were able to confirm whether, once defaulters had been traced by CHWs, they returned to the clinic. *"We engage with facility manager, she helps with problems that we have... we include her in everything" (EN, 1B)*. Clinic staff willingly accepted referrals from CHWs, and included the CHWs in training sessions: *"Sister X is not (in the CHW team), but she is very helpful. She*



For peer review only - http://bmjopen.bmj.com/site/about/guidelines.xhtml

*attended severe malnutrition training and she called all the CHWs and did a presentation for everybody*." *(EN, 1B).*

Secondly, in one clinic, the contribution of CHWs had become indispensable: *"If they (CHWs) were not there, it means you will be over worked, the clinic will always be full, to be honest. There is good communication between us." (Facility Manager, 1A)*

The third benefit was the empowerment of CHWs, who learned to negotiate with other less willing staff: *"I have a patient who doesn't want to come to the clinic and I don't want my patient to default on her HIV treatment. I said to the sister 'my patient is working at the farms so please pack medication for her. I'll ask her to come next month.' The sister scolded me because the patient did not come on her last appointment. So I begged the sister and promised to bring the patient myself. Then it was sorted, the patient got her medication and she came to the clinic with me." (CHW-FGD, 1B)*

*Negotiating acceptance and building the community's trust*

All CHW teams depended on the supervisor to liaise with key community members:



For peer review only - http://bmjopen.bmj.com/site/about/guidelines.xhtml

432 *"We established good relations with the ward councillor so whenever there was a problem I would call her." (PN, 2B)* The presence of the senior supervisor increased CHWs' credibility in the community: *"If CHWs found that I am not well, they report to the PN, and the PN would come and see me. I have noticed that when I follow the PN's advice, my health improves. We are alive today because of them." (Patient-BT, 2A)*

Box 1 describes visits to two different households conducted on the same day by the same CHW, for which she received very different support from the facility.

**Box 1: Support from the clinic facilitates CHW performance and client trust**

<u>*Case 1 : Patient defaults as nurse insists on waiting for test results*</u>

The patient, a woman on ARV with a spinal cord problem, had moved recently. She was no longer able to collect ARVs and go for physio appointment at the clinic near her previous home as she had no money for transport. She asked for a referral to a closer clinic. The nurse refused as ARV patients tended to move clinics, so patient stole the file.

The CHW took her file to the closer clinic to ask if she could collect medication for the patient. The CHW was told that a new file had to be opened and the patient had to wait for CD4 results to come back. As a result, when we interviewed her, the woman had been defaulting for the past 3 weeks.

In the interview the patient said the CHW had not followed up on her requests for food parcels as they had no food, for a physio appointment, or for her husband to be tested for HIV. *"The CHW doesn't follow up on me to see how I am doing; the only thing that she is good at is making empty promises." (Patient-AA, 3A)*

<u>*Case 2: Social worker responds to CHW request*</u>



For peer review only - http://bmjopen.bmj.com/site/about/guidelines.xhtml

> The patient was a woman whose child was knocked down and killed by a car in front of her and her children. The family were traumatised. CHW wrote a referral letter to a social worker. The patient went to the social worker and then started attending a support group. CHW also arranged for the social worker to see her husband and children at home.
>
> Patient said she trusted CHW because *"she is kind, open and she knows how to speak to people even if you are in a difficult situation. She does not gossip about her patients on the street." (Patient-Z, 3A)*

Inadequate and unpredictable support from the clinic profoundly influenced the CHW's ability to provide care for her clients, and in turn, the degree of trust between client and CHW.

Table 4 summarises the key effects of location and the level of supervision on CHW performance.

**Table 4: Impact of supervision and location on the determinants of CHW performance**

| | Clinic-based with PN and EN | Health post with PN and EN | Clinic-based with EN only |
|---|---|---|---|
| **Mentorship of EN and CHW** | Good | Depending on how busy the PN was seeing patients | • Junior ENs were not able to provide adequate training or supervision<br>• Little mentoring as facility manager was not engaged; |
| **Relationship with clinic** | Good | Poor | Poor |
| **Files** | Well managed by PN | Files at clinic were not managed by PN & EN; often lost and CHWs had to go and find them | Part of clinic filing system |
| **Effect on quality of service** | • More able to ensure that patients get care needs;<br>• Better relationship with community | • Limited service at health post; patient had to travel to clinic for anything other than basic service;<br>• Referrals were more difficult because of distance between health post from clinic; | • CHWs shied away from difficult cases, knowing that they did not have the support;<br>• Poorer relationship with community |



For peer review only - http://bmjopen.bmj.com/site/about/guidelines.xhtml

**DISCUSSION**

In this paper, we explored the effect of three different supervision and location configurations on CHW motivation and performance. We have demonstrated that experienced supervisors employed a range of strategies to train, motivate, and monitor CHWs, to improve the quality of their work. In turn CHWs were treated with greater respect by the clinic staff, making space for collaboration and support that benefited both parties. Being a near-equal of the facility manager, a PN supervisor was in a position to bridge the divide between the CHWs and clinic staff. When one of the PNs obtained a place for the CHWs inside the facility, she secured a position – physical and symbolic – for them in the health system. She drew in other staff to participate in the supervision of CHWs, further strengthening their legitimacy. Clinic staff provided advice and ensured that CHWs' patients accessed the care that they needed. CHWs traced the defaulting patients for the clinics and so improved the clinic's performance. The senior supervisor, a shared resource to both the CHWs and the clinic, was able to effectively integrate the CHWs into the health system.

In contrast, the teams in health posts found it difficult to build relationships with the clinic, particularly if the health post was at some distance from the clinic and the PN was busy with patient consultations. The EN-headed teams lacked adequate training



For peer review only - http://bmjopen.bmj.com/site/about/guidelines.xhtml

and supervision, and consequently the CHWs' performance was minimal. With little evidence of the CHWs' contribution, the social distance between the CHWs and clinic staff remained substantial. Given her junior position, the EN was not able to facilitate collaboration with the clinic to reduce this distance. Hence, despite being based in the clinic, the EN-led CHW teams were not effectively integrated into the health system.

Our study has several limitations. Firstly, despite fieldworkers asking CHWs to carry out their duties as normal, during observation many CHWs appeared to make extra effort to reach a greater number of households. We followed the same CHWs for 3-5 days to mitigate this, and the number of household visits did fall overtime, as the CHWs became used to the presence of an observer. Secondly, our data on CHW performance is based on what we learned from our respondents rather than on an objective measure of performance. Thirdly, while we selected study sites to ensure that the different supervisory configurations were included, we do not know to what extent the included CHW teams are representative of others in the same district. The study demonstrates noteworthy strengths. As far as we know, this is the first study to examine the effect of different levels of on-site supervisors as well as the location on CHW performance in a government programme. The case study approach allowed us to purposively select teams based on their different supervision/location configurations,



For peer review only - http://bmjopen.bmj.com/site/about/guidelines.xhtml

and to compare and contrast their functioning in order to understand what worked, and what did not.

CHW supervision is often no more than periodic interactions by facility staff or officials who may not understand the context and role of CHWs. This can result in bureaucratic inspection and fault-finding, rather than problem solving and support.[6, 7, 20] For example, in the Ethiopian national programme, supervisors make irregular visits from either the local health centre or district office.[21] In our study, the presence of an appropriately skilled and qualified on-site supervisor enabled on-going supportive engagement, rather than a distant supervisor who had fit supervisory visits into a busy schedule. Evidence from a rapid assessment of the South African WBOT programme in five provinces suggested that the existing facility-based staff were unable to provide effective supervision as they were already overstretched, working in under-resourced and under-performing clinics. [15] The authors concluded that CHW teams should be based in stand-alone health posts with a nurse supervisor, rather than within a clinic. Our data suggests it would be more advantageous for the nurse supervisor and the CHWs to be based at a clinic, with clear rules about how much time the supervisor can spend on clinic activities.

For peer review only



For peer review only - http://bmjopen.bmj.com/site/about/guidelines.xhtml

In the Ethiopian programme, CHWs based in health posts also struggled to make referrals and experienced dismissive attitudes from clinical staff; while where there was more regular contact, the CHWs felt valued and part of the team.[21] Studies in Uganda, Bangladesh and Bolivia have reported that contact with a higher level of the health system gave CHWs credibility with their clients.[22-24] Similar to our findings, in Mozambique supervision increased the participation of CHWs in the intervention facilities.[13]

Training and supervision of supervisors is a more neglected subject than that of CHWs.[9, 25, 26] This issue is beyond the scope of this paper, but in the second phase of our 3-year study, we are evaluating the effect on performance of a PN supervisor in building the capacity of the EN supervisors.

Globally, there are on-going efforts to scale-up CHW programmes as a means to strengthen health systems and accelerate universal health coverage. These include the call for one million CHWs in sub-Saharan Africa,[27] making CHWs available everywhere on the African continent, [28] new funding commitments from the private sector,[29] as well as the recent WHO guideline on health policy and system support to optimize CHW programmes.[30]



For peer review only - http://bmjopen.bmj.com/site/about/guidelines.xhtml

The new WHO guideline states that *"planners should adopt 'whole-of-system' approaches, taking into account health system capacities and framing the CHW role vis-à-vis other health workers in order to appropriately integrate the CHW into the health system"*.[30] The guideline also indicates that while there is benefit of supportive supervision, there is a lack of granular evidence. Our study provides rich evidence on the range of supervision strategies, how the effectiveness of these varies by the level of supervisor and whether the team has its base in a facility or not. Taking a whole-system perspective has enabled us to draw conclusions about the barriers and facilitators to integrating the CHW into the system. Below we offer recommendations based on the experience of well-functioning teams to complement current global efforts (See Table 5).

For peer review only



For peer review only - http://bmjopen.bmj.com/site/about/guidelines.xhtml

**Table 5: Programme design features and supervisory strategies that worked**

| | Recommendation | Rationale |
|---|---|---|
| Programme design | • Attachment to PHC clinic | Facilitate physical & operational integration into the health system |
| | • Team up senior and junior supervisors | Build relations, guide CHWs to navigate through the community and system, pass down know-how to junior supervisors |
| | • Setting guidelines as to how much time supervisors can spend assisting in the clinic | Help to acknowledge that the engagement is a two way collaboration, in which there are benefits for everybody |
| | • Strengthen HR management practices | Build trust, improve dialogue in the workplace, problem solving, supervision, and culturally appropriate communication |
| Supervisory strategies | • Supervise home visits | Provide opportunities to strengthen CHWs' knowledge and skills, demonstrate a strong backup for CHWs in the community, keep updated of community's status |
| | • Formal and on-the-job training | Impart knowledge and skills |
| | • Regular debriefing/feedback | Individual and collective supervision, track performance, build up teamwork spirit |
| | • Examine daily logs and registers | Ensure accurate documentation and subsequent reporting |
| | • Direct CHWs to tasks, such as tracing defaulting patients, that explicitly assist the clinic | Ensure the clinic staff are able to see the benefits of having CHWs as part of the team |
| | • Draw in clinic staff to work with and train CHWs | Improve the extent and quality of working relationships between clinic staff and CHWs, and allow CHWs to benefit from the clinic staff's expertise |
| | • Assist data collating & reporting | Ensure that CHW activities are accurately reported so that health system managers can see the benefits of the programme |
| | • Administration and logistics | Resolve administrative matters, negotiate for better work conditions, ensure CHWs are adequately equipped to deliver service |

For peer review only



For peer review only - http://bmjopen.bmj.com/site/about/guidelines.xhtml

## CONCLUSION

With high expectations of CHW programmes in improving access to care in South Africa and other similar settings, full-time, on-site supervision can make an important contribution in enabling CHWs to provide comprehensive, promotive and preventative care. In the context of an overstretched health system, senior supervision located within a clinic can enable the development of a collaboration between the clinic and CHWs, reducing the CHWs' marginalisation, and enabling their potential contribution to improving health outcomes to be realised.

## Contributorship

YHT interpreted data, wrote and revised the manuscript with JG, and approved the final manuscript as submitted.

FG and JG conceptualized and designed the study, analysed data, critically revised the manuscript and approved the final manuscript as submitted.

JDK carried out data collection process, managed data, analysed data, critically revised the manuscript, and approved the final manuscript as submitted.

NN analysed data, revised the manuscript and approved the final manuscript as submitted.

TR and HM analysed data and approved the final manuscript as submitted.

## Acknowledgements

The authors would like to acknowledge the following individuals for their invaluable contribution: the field workers, the respondents, the CHW teams, Mrs Salamina Hlahane, district manager at Sedibeng, and Mrs Bridget Lefhoedi, community-based services coordinator. Input from Professors Margaret Thorogood, David Sanders, and



For peer review only - http://bmjopen.bmj.com/site/about/guidelines.xhtml

Emmanuelle Daviaud throughout the study has been highly appreciated. We also thank these organizations for their generous support: Department of Health Sedibeng District, Gauteng Department of Health, the UK Medical Research Council (MRC) and Taiwan Ministry of Science and Technology.

**Endnotes:**

Re-hired retirees were used for three reasons. Firstly, due to the shortage of professional nurses in the system, drawing in those who had retired increased the supply. Secondly, they had a wealth of experience in community nursing, which younger nurses often did not. Thirdly, they were given an increment on top of their pension, rather than a full salary, and so cost the district less than a formally employed professional nurse.

**Competing Interest**

None.

**Data sharing**

A sample of site-specific summary is available in appendix.

**Funding**

MRC-UK under Health Systems Research Initiative (MR/N015908/1)
Taiwan Ministry of Science and Technology (105-2917-I-564-006)



For peer review only - http://bmjopen.bmj.com/site/about/guidelines.xhtml

## REFERENCES

1. Perry HB, Zulliger R, Rogers MM. Community health workers in low-, middle-, and high-income countries: an overview of their history, recent evolution, and current effectiveness. *Annu Rev Public Health* 2014;35:399-421.
2. Datiko DG, Yassin MA, Theobald SJ, et al. Health extension workers improve tuberculosis case finding and treatment outcome in Ethiopia: a large-scale implementation study. *BMJ Glob Health* 2007;2**:**(4)e000390.
3. Lehmann U, Sanders D. Community health workers: what do we know about them. The state of the evidence on programmes, activities, costs and impact on health outcomes of using community health workers. Geneva: World Health Organization 2007:1–42.
4. van Ginneken N, Lewin S, Berridge V. The emergence of community health worker programmes in the late apartheid era in South Africa: An historical analysis. *Soc Sci Med* 2010;71:1110-8.
5. Barnett ML, Gonzalez A, Miranda J, Chavira DA, Lau AS. Mobilizing Community Health Workers to Address Mental Health Disparities for Underserved Populations: A Systematic Review. *Adm Policy Ment Health* 2017;1-17.
6. Hill Z, Dumbaugh M, Benton L, et al. Supervising community health workers in low-income countries–a review of impact and implementation issues. *Glob Health Action* 2014;7:24085.
7. Glenton C, Colvin CJ, Carlsen B, et al. Barriers and facilitators to the implementation of lay health worker programmes to improve access to maternal and child health: qualitative evidence synthesis. *Cochrane Database Syst Rev* 2013;10.
8. Kok MC, Kane SS, Tulloch O, et al. How does context influence performance of community health workers in low- and middle-income countries? Evidence from the literature. *Health Res Policy Syst* 2015;13:1-13.
9. Pallas SW, Minhas D, Pérez-Escamilla R, et al. Community health workers in low-and middle-income countries: what do we know about scaling up and sustainability? *Am J Public Health* 2013;103:e74-e82.
10. Kok MC, Dieleman M, Taegtmeyer M, et al. Which intervention design factors influence performance of community health workers in low-and middle-income countries? A systematic review. *Health Policy Plan* 2014;30:1207-27.
11. Bosch-Capblanch X, Liaqat S, Garner P. Managerial supervision to improve primary health care in low- and middle-income countries. Cochrane Database Syst Rev 2011;9.



For peer review only - http://bmjopen.bmj.com/site/about/guidelines.xhtml

12. Rabbani F, Shipton L, Aftab W, et al. Inspiring health worker motivation with supportive supervision: a survey of lady health supervisor motivating factors in rural Pakistan. *BMC Health Serv Res* 2016;16:397.
13. Madede T, Sidat M, McAuliffe E, et al. The impact of a supportive supervision intervention on health workers in Niassa, Mozambique: a cluster-controlled trial. *Hum Resour Health* 2017;15:58.
14. Ameha A, Karim AM, Erbo A, et al. Effectiveness of supportive supervision on the consistency of inter-grated comunity cases management skills of the health extension workers in 113 districts of Ethiopia. *Ethiop Med J* 2013;52:65-71.
15. Marcus TS, Hugo J, Jinabhai CC. Which primary care model? A qualitative analysis of ward-based outreach teams in South Africa. *Afr J Prim Health Care Fam Med* 2017;9:1-8.
16. Schneider H, Schaay N, et al. The challenges of reshaping disease specific and care oriented community based services towards comprehensive goals: a situation appraisal in the Western Cape Province, South Africa. *BMC Health Serv Res* 2015;15:436.
17. Department of Health, South Africa. Provincial guidelines for the implementation of the three streams of PHC Re-engineering. http://www.jphcf.co.za/wp-content/uploads/2014/06/GUIDELINES-FOR-THE-IMPLEMENTATION-OF-THE-THREE-STREAMS-OF-PHC-4-Sept-2.pdf (accessed 23 Jan 2017).
18. Trafford Z, Swartz A, Colvin CJ. "Contract to Volunteer": South African Community Health Worker Mobilization for Better Labor Protection. *New Solut* 2018; 27:648-66.
19. Nxumalo N, Choonara S. *A rapid assessment of ward-based PHC outreach teams in Gauteng Sedibeng district–emfuleni sub-district.* Johannesburg: Centre for Health Policy, University of the Witwatersrand, 2014.
20. Roberton T, Applegate J, Lefevre AE, et al. Initial experiences and innovations in supervising community health workers for maternal, newborn, and child health in Morogoro region, Tanzania. *Hum Resour Health* 2015;13:19.
21. Kok MC, Kea AZ, Datiko DG, et al. A qualitative assessment of health extension workers' relationships with the community and health sector in Ethiopia: opportunities for enhancing maternal health performance. *Hum Resour Health* 2015:13:80.
22. Bartos A, Tenorio A, Saunero R, et al., *Extending the duration of exclusive breastfeeding in El Alto, Bolivia through a community-based approach and the provision of health services.* Geneva, Switserland: Child Health and Nutrition Research Initiative, 2009.



For peer review only - http://bmjopen.bmj.com/site/about/guidelines.xhtml

23. Nankunda J, Tumwine JK, Soltvedt A, et al. Community based peer counsellors for support of exclusive breastfeeding: experiences from rural Uganda. *Int Breastfeed J* 2006;1:19.

24. Haider R, Ashworth A, Kabir I, et al. Effect of community-based peer counsellors on exclusive breastfeeding practices in Dhaka, Bangladesh: a randomised controlled trial. *Lancet* 2000;356:1643-7.

25. Daniels, K., et al., Supervision of community peer counsellors for infant feeding in South Africa: an exploratory qualitative study. *Hum Resour Health* 2010;8:6.

26. Rowe AK, Onikpo F, Lama M, Deming MS. The rise and fall of supervision in a project designed to strengthen supervision of Integrated Management of Childhood Illness in Benin. *Health Policy Plan* 2009;25:125-34.

27. Singh P, Sachs JD. 1 million community health workers in sub-Saharan Africa by 2015. *Lancet* 2013;382:363-5.

28. The Lancet Global Health. Community health workers: emerging from the shadows? Lancet Glob health 2017;5:e467.

29. Winters N, O'Donovan J, Geniets A. A new era for community health in countries of low and middle income? *Lancet Glob Health* 2018:6:e489-90.

30. World Health Organization. WHO guideline on health policy and system support to optimize community health worker programmes. http://apps.who.int/iris/bitstream/handle/10665/275474/9789241550369-eng.pdf?ua=1 (accessed 13 Dec 2018).

For peer review only

Case site summary
Site: A Author(s): JG Date:22 02 2017

| | |
|---|---|
| **Model** | Clinic based team, although doesn't perform all standard clinic services (e.g. TB)<br>One PN, two ENs |
| **PN** – background/ experience, age, role, actual responsibilities | Professional nurse, midwifery, community health worker, at X for 6 years then Y hospital, retired at X, then went to the UK for X years .. started to work in Y at 62 .. now 72 years<br><br>**PNO:** once CHW have gone into the field, and have explained several things to the FW, spends an hour seeing patients.. and then leaves (so no meeting after CHW return)<br><br>**PNO_NM:** interview with FW, patient consultations for 2 hours; after 11.20 she reads patient files / notebooks; She also see family planning patients; (FW-PN doesn't seem to have a lot of work to do) |
| **EN**– background/ experience, age, role, actual responsibilities | <u>**EN01**</u> **- responsible for ward X**<br>**X** years old, became a EN in X, worked in private clinic before here, not from the area<br>FW comment: the EN seems passive.. maybe due to helplessness around CHW.. she struggles to maintain basic level of supervison by checking and doing CHW stats. Not clear how much time she spends in the clinic<br>*JG: from first meeting we know this EN doesn't like going out into the community .. it may be that she prefers to work in the clinic. When it comes to observations, she didn't actually do the stats because EN02 had given it to the data capturer to do because EN02 was going on training .. so sounds as if EN02 does most of the data reporting etc.*<br>**Observation day 1:**<br>Private meeting between PN and EN<br>Check on crèche that children have been immunized – no really confirmation of this<br>HH1: EN manages interaction with hh – follow up<br>Check on a pregnant woman at the crèche: registration done by CHW<br>HH2: patient there, but says that gate is locked;<br>HH3: patient not there<br>HH4: EN leads the interaction – follow up<br>FW: thinks EN went into the field because FW was there to observe .. but not real need for her to be there.<br>**Observation day2:**<br>Works in clinic as they are short staffed – so can't be observed; lunch and then knocks off as ½ day;<br>Next week she is on training so will ask receptionist to help with the stats. She will sign books to show she has checked them<br><br><u>**EN02**</u> used to work in critical care unit in private hospital<br>Not sure if I am a nurse any more .. but I feel more useful<br>Much more active, tries to solve problems, has set up whats app group, able to provide supervision<br>**Observation day 1:**<br>Creche campaign; EN is leading activities<br>**Observation day 2:**<br>Works in clinic as short-staffed; sorting patients and then doing child immunisations;<br>Comes out to check on CHW to allocate them to FW<br>HH1 CHW leads<br>HH2 CHW checks patient, but EN worried so asks questions about stomach<br>HH3: EN leads<br>HH4: EN asks about granny; in hospital; EN is shocked because here only 2 weeks ago, checks whether she has enough medication etc *(suggests that EN02 does go regularly to the field)*<br>HH5: jointly doing activities<br>HH6: CHW leads, didn't ask about child<br><u>HH7</u>: Jointly leading<br>Return to clinic at about 12 noon<br>*FW comment: EN is very supportive of CHW*<br>**Observation day 3:**<br><u>HH1</u>: on way to support group visited a lady who had given birth to twins- just checked on babies<br><u>Support group</u>: EN sits at a distance, CHW know how to do their work; she only intervened when a BP was too high, and when difficult TB case arrives (HH interview: CHW bring sputum bottle, but patient fails to provide) ; not 12 noon so there is time to see a few households |

For peer review only

| | |
|---|---|
| | HH2: not allocated to a CHW, but she checks on him because he is sick with TB; EN leads (as she hasn't been before); EN says they will come and do registration even though not in demarked area<br>HH3: car accident victim and HIV<br>HH4: EN quiet, CHW leads<br>Back on counsellors place by 1pm after 30 mins walk<br>**JG:** *EN seems works hard, happy to go and find extra households, seems to know some households – evidence that goes into the field regularly*<br>**Observation day 4**<br>Discussion about working on Fridays they do stats – cHW said we don't; EN said that is only the last Friday of the month .. so today they are going into the field; Later EN says later .. eish we are working on a Friday .. this is slavery<br>HH1; came to give referral letter for NGO.. which is now closed for the year;<br>CHw says I will take you to the patient you didn't manage to see on Monday<br>HH2: patient discharged from hospital in pain; EN tried to help – offered to call an ambulance, but woman said no because nobody to go with her; suggested clinic on Monday – ok, daughter will take me; asked for daugther's number; can't get it out of the phone<br>HH3: checking on patient that they referred two days ago; she hadn't gone ; doesn't take her treatment;child without immunization; EN quite upset<br>HH3: healthy baby; EN happy ☺ |
| **CHWs** – average details across team (Number, age, gender, years' experience, education) | X CHWs in FGD, (X reported to part of team) , all female, (another X waiting to be registered. PN wants them back)<br>Average age 39 (range25-49)<br>64% of have some secondary education;<br>29% have matric<br>7 years on average as a CHW (range 3-16)<br>Average number of households=262 (248-317) (only Sicelo and Biopatong gave specific numbers per CHW)<br>25 mins walk from home to clinic (range 2-50)<br>18 mins walk from clinic to households (2-45)<br>93% have phase 1; 21% have phase 2 |
| Community profile (eventually draw in survey data) | **PN:** Ward X contains a large informal settlement **(FDG SM?)**<br>Ward 10 predominately a township **(FDG AS?)**<br><br>**PN:** We started going from house to house, counting people during the registration.. according to where the CHWstayed.. initial plan was 250 households, but didn't work .. either 190 or 200 (if medical aid we skip it)<br><br>People report to us if somebody is sick, they will even break down the door<br>**ENO2** (ward X) – area is huge - shortage of CHW<br>3 areas within ward X<br>Ext X and X informal settlements / shacks – 5 is too big<br>Ext X – RDP house<br>People are moving to Rdp houses, go back 3 days later and people have moved..<br>Lots of Zim patients, but language not a barrier<br>Informal settlement, lots of crime, didn't feel safe at beginning,, but not easy for CHW to be robbed because most of them are well known in community |
| Challenges? (Not general) | **FM:** Storage is a problem – for files,<br>Safety .. too many break ins in the clinic<br>CHW need more training, and then more CHW will hopefully come later<br>**FDG-SM**<br>Equipment – one CHW has take the BP machines that side .. so you call her to ask her to bring this side .. call from counsellor .. and we don't have machines<br>Behaviour of nurses – some patients refuse to come to the clinic because of the bad treatment. However we encourage them for the sake of their health to ignore the nurses attitude P11<br>Queuing – it irritates the patients .. so it gives us a problem when we try to encourage the patients to go to the clinic ..<br>Clocking in WBOT promised we would work around where we stay.. they promised a clock in Ward 8 .. we have found a place for it .. it is tiring to have to come to ward 10 to clock in .. go back to ward 8 and then come back to the clinic .. it is a distrance and it is hot or raining!<br>Stipend, job security, career development, smart purse<br>**FDG_AS**: as above plus job safety, training, recognition and appreciation<br>We can't even go to the bank with this card, only to shoprite .. otherwise they charge us |

For peer review only

| | |
|---|---|
| | **EN02 Obs:** currently leave is pause .. because smart purse says 13 days, contract says 21 days – hey have sent a letter to smart purse – EN really interested in solving problems .. but sometimes above her |
| Planning (of the day) | **PN:** I have paired them, an intelligent one with a less intelligent one |
| Weekly pattern of activity (what do they do when?)<br>- overtime | **FM:** one of their strengths is tracing .. they will find the person p18<br>CHW say that they do overtime<br>**FM:** says they don't<br>**FM:** may be ENs help out in the clinic 1 day out of 5<br>Did two campaign days during FW<br>**EN01:**<br>Run support group for elderly patients on Wednesday<br><br>**FDG-SM:**<br>We follow up on defaulters<br>Household registrations each day, - it is not possible to do registrations at one time of the year<br>Home-based care – we don't do much of this ..<br>we refer to social services<br>*JG: not convinced there is a day for this, and a day for that .. rather that they do all of the activities when needed.*<br><br>**FDG-AS:**<br>With Kgatelopele the first week I deliver medication, the second I will check if they are taking it properly by counting.. if it doesn't balance .. then I will ask her why .. then I will report to the clinic to the cinic that I am following up on this person . |
| Daily pattern of activity | 6 hh per day (for two CHW) (average is 5)<br>13 mins average per household (average is 12)<br>10 mins average travelling between households (average is 14)<br>5 hours work per day (average is 4.40)<br><br>5% of activities are meeting with supervisors (not time)<br>20% of activities are HIV as condition; (TB 5.5%)<br>52.75% of activities are other chronic<br>5.49% as mother and child<br>53% of recipients are elderly<br><br>Between 8-9 am do training<br>**EN02**: training and gym until 9am.. e.g. with new machines I take two CHW and show them so that they know what to do in the field<br>You can see a big difference between those who have attended the trainings..<br><br>On a Tuesday they are taking medication to patients, and from there to check on what ever<br>So if we have gone to 2 households, taking treatment, then I pick one house, any house that we haven't been to before ..and do a registration ..<br>Cos you find that their focus is on the patients that they know, the patients that they know<br>But with me I am always squeezing in just one house that has never been registered .. so I don't totally change their routine .. just one HH and you will find problems<br><br>**EN01:** 8am clock in and wait outside until 9am when go to households (no mention of training)<br>CHW work in pairs must see 6 households 3, 3, lunch break is between 12-1<br>They don't just to go their patients, they also visit other households after a longer break when no new patient is identified. People in the area also inform the CHW when there is somebody who is sick;<br><br>**FGD-SM:** 6 households per day each and households registrations is too much .. and we have to be back here by 13.30 .. our lunch is at 12 noon. We discuss issues we have with the nurse and pack medication to deliver (next day or on way home)?)<br>We write everything in our note book .. and then we note in our diary when we have to return to a household<br><br>**FDG-AS:** planning seems to be more about which of your houses and which of mine<br>• Last time they told us we had to see 6 patients a day per CHW .. now they have changed it to 4<br>• If you are doing household registrations .. you can't do 6 per day p13 |

| | |
|---|---|
| | • Sometimes we do home-based care, if the patient is living on her own and she is on treatment . .if ask if she has eaten .. if she has't I cook for her;<br>• We refer them .. the provider needs 8rand<br>• Sometimes we teach the family how to bath them |
| Record keeping & Stats – how & when<br>- How notes/info are kept<br>- How they are consolidated<br>- Patient files<br>- Follow ups | **FM:** Has improved since two ENs arrived<br>WE don't have email and our transport is irregular;<br>Now doing it once a week .. we used to do it once a month .. but then it was late<br>Two ENs are taking responsibility and they forward to the data capturer and then we come back and chat about it (Sister Tenqui and FM?)<br>**PN:** they have notebooks that they write in .. not more than 5/6 households per notebook .. (*so does that mean that they have 40 notebooks?)* I read those books every time I get a chance<br>**EN02:** she does monthly stats, she checks individual stats, talks to CHW saying why do you say this, or why haven't you write x, gives training to CHW.<br>In training we were told it would be easier if we did it weekly so I said to CHW lets do it on Fridays .. lets not go ot households but do stats instead .. now we are doing it monthly because we are faster now<br>From CHW to EN to facility manager<br>*JG: this is why they don't work on Fridays.*<br>**FDG-SM** we do stats daily .. at lunchtime we record evertying (some confusion about recording number of children)<br>**FDG-AS:** the OTL takes time to check them<br>I do them in my lunch hour, or at home, or when we are seeing my partner's patients |
| Referrals<br>- Forms/documentation<br>- Queueing<br>- Files | **FM** :Patient meant to show form at door and then goes to see Sister X, who then completes the form and gives back to the carer (if another nurses see patient they also complete the form)<br>**EN02:** Report number of referrals in stats as well<br>**FDG-SM**<br>"The referral form is causing trouble with the patients. The patients are supposed not to follow the queue, they should go straight to the PN but the patients are been shouted, told to go and follow the queue. Now the patients are fighting with us because we told them that with the referral they don't have to queue."p 15 |
| Medication delivery | **FM:** ENs can't come issue medication; so tengiwe does it . and if she is not here, they come to me with the vital signs data and the pill count, the condition of the patient, and the next clinic data. If the ENs are there the CHW will do this<br>**FDG-AS:** when you count their medication, sometimes they start shouting at you . you try to tell them that I am tyring to help you .. |
| Relationships Clinic – WBOT team | Other sisters will give medication out if necessary<br>CHW refer patients to specific sisters eg. For TB<br>**EN02** We do in-service training .. Sister X is doing EPS .. she is very helpful .. she is not WBOTS but she is very helpful - she is a staff nurse, but she has got more xperience … she attended severe malnutrition training .. she called all the CHW and patients .. shw as doing a presentation for everybody .. so it is not only us doing that .. She is also helping when we need something There is support from the clinic.<br>We engage with the FM for everything, problems we have, reporting .. she is part of the community, we include her in everything<br>District coordinator – I have only seen her once, when she came with those two ladies from wits !<br>Training person at X– if I have aproblem a message the OTL group, or even phone her .. she is giving support.<br>**FDG-SM**<br>"At the clinic we are treated as if we are not part of the clinic, they talk to us as if we are children. They shout at us in front of the patient that you brought for help. We don't have support at all. Another thing, they don't want us to eat breakfast but they eat when they come to the clinic. We consult TB patients on empty stomach. I did raise that to the PN and she said we should eat at home. Then I asked why she is not also eating at home in the morning and see if she can do it. In the morning we prepare our children to go to school, others have husbands to take care of before we leave at 7:00am, and we have to walk. We don't have time to have breakfast at our houses in the morning. We only have breakfast and lunch together at 12:00pm. There is no support at all." P15<br>**FDG-AS:**<br>"the sister in charge and our OTLs are treating us like.. I don't know .. as you can see we have no place to stay, no chairs, no table, nothing .. we came with the idea of starting the day with a prayer like in hospitasl.. but the sister in charge says we won't do such a thing here .. so we stopped because we are powerless' p14<br><br>**Any conflict with OTL or clinic staff** |

For peer review only

| | |
|---|---|
| | “We are all going to talk neh .. add to what I say .. we have our sister in charge, but we have another one .. the CHWs know who I am talking abut .. this sisteris controlling us more than our OTL” .. P21<br>“she even shouts at us infront of the patients. We are adults, mothers and we have our own houses and when we are here she treats us like children. She does not respect us.” P21<br>“And sometimes we walk in, she shouts and says ‘hey you is that a uniform, that is not the way we dress here.’ Where are we going to get the new navy pant? That pant costs R190-R200.00. All that shouting she does it in front of everyone at the clinic. That is not okay and when you talk back to her, she will say that she is not our OTL and does report to us.”<br>**Participant**: “We want her to be out of the WBOT team. We want to report to our OTL only. She is responsible for other nurses not us.”<br><br>“My challenge as a CHW is that when I do home visit, some households do not have food to eat and the patient is on treatment. So I have to empathise with the patient, contribute something so that the patient can eat. When I come to the clinic and ask for this porridge, the clinic staffs tells me that I have to come with the patient so that they can see the patient. That is a big problem because the patient is sick and has no transport money to go to the clinic. So yah it hurts.” P20 |
| Relationship EN & PN | **EN02:** if I need clarity I will phone here |
| Supervision of CHWs (including training) | **PN:** I read their books whenever I can; if they have difficult cases they bring them straight to me .. and I take the car and drive to see the issue ..<br>**EN01:** Most of the CHW are older than her.. they don’t always listen to the EN. When they only have one household to visit they have all sorts of stories to get away. She reported this to the FM who said she would sort it out.<br>Two CHW communicate on behalf of the rest ..<br>They report to the EN when they have found a defaulter, they then revisit and then report<br>Weekly or bi-weekly she checks the activities of the CHW<br>FM told CHW not to come to work during strike<br><br>**EN02:** supervises 8 CHW, 5 have not been registered so they are using the book. If she knows CHW have much work to do, she leaves with them at 8 rather than 9<br>Different types of CHW – counsellor, peer educators, rest are CHW; one CHW is the leader whose job it is to forward complaints to her<br>If I have been on training I will ask how did it go with that patient, we have an whatspp group so we can communicate that weay .. if I am not around, they will send me a whatsapp and I will say do this or that.<br>I spend the whole day with them, .. and then I give them a lift home<br><br>Since the strike they have not been the same .. some they understand, some they are aggressive … so I said if you need something, or clarity, sit as CHW, write a memo /list and let your head CHW delivery it .. according to the hierarchy – this really helps, otherwise we have too many complaining and nothing will be sorted out<br>e.g. bags – why only 10 bags; so they wrote it down, I took it to my PN, and the facility manager who was able to sort it out; we discussed how they are going to use the equipment<br>*JG – example of sorting things out*<br><br>It goes back to the in-service training … when I am compiling the stats I am able to see this one is struggling .. when I do supervision I can see this one didn’t do it right … I keep quiet in the house,, but immediately we step outside .. as we are walking I do spot training<br><br>Both FM and EN01 had stories of truancy (going home and coming back to clock out);<br>EN02 uses her car to drive them back to the facility if they need to clock out, or allows them to sign out tomorrow if they are working late<br>**EN 02** Sometimes they come back early because of the distance.. because it can be an hours walk … you can waste 2 hours a day walking backwards and forwards<br>**FDG-SM** do you work overtime? If we do household registration, and we don’t find anybody who can has the information, we go back in the afternoon or the weekend .. .. Sometimes we deliver medication in the afternoon.. sometimes we are do vital signs at 7.45 |

For peer review only

| | |
|---|---|
| | **FDG-AS:** if some people are very sick we monitor them over the weekend<br>Our team leader is supportive .. if the patient wants to know something .. I ask her |
| Supervision of ENs | **FM:** do induction and appraisals of EN; provide training – HIV (going on Monday), TB (one has gone the other will go in 2017), EVP<br>**EN01:** if sister X is not around she goes to the FM and she provides a solution<br>They help out between 7.30 and 12.00 at the clinic when the clinic is full. When the clinic needs them they pick two CHW to stay and help the EN .. but they are free to say no<br>**EN02:** The facility manager makes sure we attend regular training to uplift ourselves She organizes workshops with CHW to train them on what she has learnt<br>Now I can lead campaigns – cos I understand everything about the campaign – so you see there really is development here..<br>My job is with WBOTs. 70% in the field, 30% in WBOT admin .. if I am not busy I help with vital signs stations.. it is not part of my job but I do it anyway .. I refer my patients and the nurses see them .. So there is teamwork .. The support system is great so I never felt pressure to leave my wBOT duties<br>**FDG-SM**<br>"And sometimes when you go with the OTL, the patients are feeling uncomfortable because she shouts at them. And we are unable to tell her that what you are doing is wrong, you are not supposed to talk like that with patients. Now the patients are telling us that we are not welcomed in their houses if we come with the OTL."<br>*JG- not clear which OTL is this. .. although know it is ward X* |
| Level of knowledge of CHWs | **FM:** Depends on education of the individual - some will do as you ask, others you need to dig deeper ..<br>**PN**: they are done some of the training (phase 1 and 2)<br>Are they able to implement it ? some are .. others are dragging, but they are trying p7<br>**FDG-SM – what cases aren't you able to deal with?**<br>Sick patient with no food >refer<br>Elderly patients who needs to be in old age home |
| Travel (time, mode, particular features/challenges) | |
| Resources Available | |
| Resources Needed | Have to buy batteries, transport, airtime (although do call-backs), forms, uniform out of own money (although towards the end, the CHW were wearing pink shirts)<br>Don't have office space, or masks for working with TB patients<br>**PN:** forms .. yes, one day a teacher was here from the primary school.. and I asked him to make copies<br>*No photocopier*<br>They need a permanent structure so that they can do maternity<br>We are using our own transport - that is what is irritating<br>**EN02:** Shortage of bags<br>**EN02:** I ma suing my own petrol, my own cellphone, chw use their own airtime.. sometimes they even call patients.<br>We need a place to meet as CHW /OTLS; patient files are stored in PN's room<br>We need our own social worker<br>**FDG_SM – description of the bag**<br>We are meant to screen everybody over 25 years for diabetes.. but we don't because the strips are not enough<br>The machine is already broken .. it is not strong<br>We don't have an office, airtime, batteries (we have to buy out of our money)<br>**FDG-SA:** we were given 10 bags for 17 of us; my machine isn't working.. I reported it .. it is embarrassing when it doesn't work; the strips are finished; we report to the sister who says that the patient must come to the clinic<br>You use your own airtime to call an ambulance;<br>If you are tracing, if you don't find that person at the address, you call them using your own airtime .. otherwise how are going to find them them<br>What makes us not to achieve more is that when we come to the clinic and ask for some more equipment, we are told that that they don't have' |
| Community relationships | **FM:** Initially not accepted, were chased away .. now they are accepted. ..have built rapport<br>community unhappy when CHW on strike (they were saying nurses were on strike p6 |

For peer review only

| | |
|---|---|
| | can do dressings which patients appreciate<br>**PN**<br>• The relationship between the community and the CHW .. 'one day we were out walking with Dr Y and Dr X.. and somebody asked the CHW how come you are walking with so many men' .. she answered they are doctors we are working together' Dr X asked theperson .. how do you know my nurse? .. she brings me medication every month P6 .. Dr X was very excited that they were well known in the community p6<br>• That is why we allocate houses to them in their area.. because then people can call them over the weekend . and then they give us a report and say .. a patient was giving birth .. and so we called an ambulance etc .. it is better if the CHW is in the area<br>• We don't have a clinic committee .. but we have a very good relationship with the ward councilor (ward X?) (EN02 as well – if there is somebody who is sick he calls me, so we work toghether)<br>• I want to start a support group for young people with HIV<br>**EN02**<br>The community is aware of us .. they are coming to us. Even the referral forms its good<br>People are taking treatment now. I think there is lesser tracing on ARV patients, most defaulters you will find are TB. Our stats are going up<br>**Reason for success**<br>Employing us as staff nurses .. cos if the CHW see you as supporting them, trying to understand where they are coming from, especially with this employer thing is mixed up … That is why they refused to go on strike today<br>**EN02**<br>When they were on strike for 3 months .. the patients were complaining .. where have you been, we have been waiting for you .. they decided not to go to the clinic but wait until the sisters were back .. so they really rely on them<br>**FGD-SM**<br>We get the list of defaulters .. but now there are few because of our work .. people have now accepted their illness .. people do appreciate us.. just come and disclose to us .. and later that person will come and thank you for the work you have done p5<br><br>Iyhoo, I'm so proud about my patients, they get better. Others I found them bedridden but because of us they have recovered. Even if they don't give me any material thing, but when I pass them on the street and she/he tells others that she/he is walking because of me, I feel satisfied because at least I have made other people better." P15<br><br>"In ward 8 we don't have any conflict with the community because the ward councilor had introduced us during the public meeting. They know us and they trust us in a way that when they need help even if it is during the night, they wake us. People got that belief that when the CHWs call the ambulance, the ambulance arrives quickly. So we are don't mind because we know the community rely on us."<br><br>We already said, they are able to come to us. Like no the nurse at the clinic is unapproachable, she talks to the patients the way she likes and the patient keep quiet but when they see us, they tell us what happened with the nurse at the clinic. So it is easy for them to talk to us than to the nurses. Other patients said if the nurse comes to their houses and speaks the way she speaks when she is at the clinic, we are going to hit her. Then we try to calm them down." P15<br><br>If my neighbour is sick I ask my partner to attend to her.. cos I don't want the neighbour to say that I talk about her ..<br><br>"The community things we are their helper, they love us. In everything that we do, they stand with us." P25<br><br>"To honest the patients trust us and they appreciate us. I have a patient who is HIV and she doesn't want to come to the clinic and I don't want my patient to default. So I promised her that I will assist. Then I came to the clinic and talk to the sister. I said sister my patient is working at the farms so please pack medication for her, here is her clinic card. I'll ask her to come next month. The sister scolded me because the patient did not come to the clinic on her last appointment. So I begged the sister and I promised the sister that I will bring the patient myself. Then it was sorted, the patient came to the clinic with me and she got her medication." P18 |
| Relationships with other NGOs, | **EN02**<br>We work with policing forum, ward counsellor, we have their numbers .. we do campaigns together .. community and health campaigns (e.g.creches) |

For peer review only

| | |
|---|---|
| networking, knowledge of context (draw on what exists & links clients) | Household registration for municipality .. although you ask these questions about income.. they think you are bringing food .. so they make you feel like you are making empty promises)<br>NGO A – I have referred too many people there .. but there results are not good.. if people don't have an ID they come back with nothing. We need our own social worker |
| Overall impression<br>- Incl do CHWs feel valued?<br>- Other notes | During the strike some of the CHW tried to work .. but the EN and the FM told them not to<br>**EN02:** strike started again today but CHW refuse to be part of strike (strike is for better employment issues, high bank card deductions (20R), lack of communication from smart purse, uniform); eventually they had to accepted it .. so not joining strike today<br><br>**Can the programme be led by staff nurses ?**<br>**EN02** I am saying this because with staff nurses in the community … I can deal with almost 90% of the problems .. but as a staff nurse I am not allowed to give out treatment.. if I pre-pack medication .. it has to be checked by the professional nurse .. also I can't consult |
| Interesting points | • FM is involved, and knows how the WBOT programme runs, clinic was set up and the same time as the WBOT<br>• EN02 know there is a reciprocal relationship between WBOT and clinic and need to maintain that ..(helps when clinic is busy and they see her patients)<br>• If clinic is not welcoming to patients .. difficulty in persuading patients to go to the clinic .. So success of WBOT is dependent on quality of care in clinics<br>• In EN only model – a PN has to be available to dispense the medication (or check it?)<br>• It seems that EN02 who goes out into the community has a better relationship with the CHW, the trust each other more .. a lot of the problems are about lack of trust<br>• EN 01 is a lot more passive and prefers to work in the clinic;<br>• PN sees patients but doesn't seem to meet with CHW when they return .. EN02 knows households and really works with CHW |

For peer review only

Consolidated criteria for reporting qualitative studies (COREQ): 32-item checklist

| Item | | **Guide questions/description** | |
|---|---|---|---|
| **Domain 1: Research team and reflexivity** | | | |
| **Personal Characteristics** | | | |
| 1. | Interviewer/facilitator | Which author/s conducted the interview or focus group? | **Field workers trained by researchers collected data** |
| 2. | Credentials | What were the researcher's credentials? *E.g. PhD, MD* | **PhD, MD, MS and MA** |
| 3. | Occupation | What was their occupation at the time of the study? | **Professors, researchers, postdocs, and PhD fellows** |
| 4. | Gender | Was the researcher male or female? | **6 females + 1male** |
| 5. | Experience and training | What experience or training did the researcher have? | **Health policy, qualitative, health systems research, social work** |
| Relationship with participants | | | |
| 6. | Relationship established | Was a relationship established prior to study commencement? | **No** |
| 7. | Participant knowledge of the interviewer | What did the participants know about the researcher? e.g. *personal goals, reasons for doing the research* | **Researchers and fieldworkers explained to participants the reasons for doing the research** |
| 8. | Interviewer characteristics | What characteristics were reported about the interviewer/facilitator? e.g. *Bias, assumptions, reasons and interests in the research topic* | **Researchers explained the aims and objectives of the study to the participants.** |
| **Domain 2: study design** | | | |
| Theoretical framework | | | |
| 9. | Methodological orientation and Theory | What methodological orientation was stated to underpin the study? *e.g. grounded theory, discourse analysis, ethnography, phenomenology, content analysis* | **Case study** |
| Participant selection | | | |
| 10. | Sampling | How were participants selected? *e.g. purposive, convenience, consecutive, snowball* | **Purposive** |
| 11. | Method of approach | How were participants approached? e.g. *face-to-face, telephone, mail, email* | **Either telephonically, or face-to-face.** |
| 12. | Sample size | How many participants were in the study? | **201 individuals were being observed or/and interviewed.** |
| 13. | Non-participation | How many people refused to participate or dropped out? Reasons? | **Only one senior nurse supervisor. However, she didn't really refuse, but only rescheduled a number of times and we eventually took it as a reluctance to participate.** |
| Setting | | | |
| 14. | Setting of data collection | Where was the data collected? e.g. *home, clinic, workplace* | **Home for household members; workplace (incl. community and facility) for CHWs and supervisors** |

For peer review only

| | | | |
|---|---|---|---|
| 15. | Presence of non-participants | Was anyone else present besides the participants and researchers? | **Yes, during observations, where there were community members and clinic staff.**<br>**No, during focus group & interview.** |
| 16. | Description of sample | What are the important characteristics of the sample? *e.g. demographic data, date* | **Characteristics of the sample related to research are provided in Results, on pages 15-16, lines 204-224.** |
| Data collection | | | |
| 17. | Interview guide | Were questions, prompts, guides provided by the authors? Was it pilot tested? | **Yes.**<br>**Pilot tested between researchers and fieldworkers (facilitators).** |
| 18. | Repeat interviews | Were repeat interviews carried out? If yes, how many? | **Follow-up focus group discussions at 4 of the clinic-based CHWs** |
| 19. | Audio/visual recording | Did the research use audio or visual recording to collect the data? | **Interviews and focus discussions were audio recorded for transcription, as they were done in local language.** |
| 20. | Field notes | Were field notes made during and/or after the interview or focus group? | **Yes, notes were made for every piece of data, both during data collection and afterwards.** |
| 21. | Duration | What was the duration of the interviews or focus group? | **5 months** |
| 22. | Data saturation | Was data saturation discussed? | **Yes** |
| 23. | Transcripts returned | Were transcripts returned to participants for comment and/or correction? | **No** |
| **Domain 3: analysis and findings** | | | |
| Data analysis | | | |
| 24. | Number of data coders | How many data coders coded the data? | **All 7 authors** |
| 25. | Description of the coding tree | Did authors provide a description of the coding tree? | **No** |
| 26. | Derivation of themes | Were themes identified in advance or derived from the data? | **Both** |
| 27. | Software | What software, if applicable, was used to manage the data? | **Done manually** |
| 28. | Participant checking | Did participants provide feedback on the findings? | **Feedback has been given to provincial, district and facility management, as well as CHW supervisors and CHWs in the intervention sites in various forms, including facility level workshops. Participants generally agreed with the findings.** |
| Reporting | | | |
| 29. | Quotations presented | Were participant quotations presented to illustrate the themes / findings? Was each quotation identified? e.g. *participant number* | **Yes** |
| 30. | Data and | Was there consistency between the data | **Yes** |

For peer review only

| | | | |
|---|---|---|---|
| | findings consistent | presented and the findings? | |
| 31. | Clarity of major themes | Were major themes clearly presented in the findings? | **Yes** |
| 32. | Clarity of minor themes | Is there a description of diverse cases or discussion of minor themes? | **Yes** |

For peer review only
